# Class III hybrid cluster protein homodimeric architecture shows evolutionary relationship with Ni, Fe-carbon monoxide dehydrogenases

**DOI:** 10.1038/s41467-023-41289-4

**Published:** 2023-09-14

**Authors:** Takashi Fujishiro, Kyosei Takaoka

**Affiliations:** Department of Biochemistry and Molecular Biology, Graduate School of Science and Engineering, Saitama University, Shimo-Okubo 255, Sakura-ku, Saitama, 338-8570 Japan

**Keywords:** X-ray crystallography, Metalloproteins, Enzymes

## Abstract

Hybrid cluster proteins (HCPs) are Fe-S-O cluster-containing metalloenzymes in three distinct classes (class I and II: monomer, III: homodimer), all of which structurally related to homodimeric Ni, Fe-carbon monoxide dehydrogenases (CODHs). Here we show X-ray crystal structure of class III HCP from *Methanothermobacter marburgensis* (*Mm* HCP), demonstrating its homodimeric architecture structurally resembles those of CODHs. Also, despite the different architectures of class III and I/II HCPs, [4Fe-4S] and hybrid clusters are found in equivalent positions in all HCPs. Structural comparison of *Mm* HCP and CODHs unveils some distinct features such as the environments of their homodimeric interfaces and the active site metalloclusters. Furthermore, structural analysis of *Mm* HCP C67Y and characterization of several *Mm* HCP variants with a Cys67 mutation reveal the significance of Cys67 in protein structure, metallocluster binding and hydroxylamine reductase activity. Structure-based bioinformatics analysis of HCPs and CODHs provides insights into the structural evolution of the HCP/CODH superfamily.

## Abstract (web summary)

Here, the authors present an X-ray crystal structure of a class III hybrid cluster protein (HCP), structurally similar to Ni, Fe-carbon monoxide dehydrogenases (CODHs), enabling conclusions to be drawn regarding the structural evolution of HCP/CODH superfamily.

## Introduction

Hybrid cluster proteins (HCPs) are unique Fe-S-O-type metallocluster (hybrid cluster)-containing enzymes^1–4^. They play key roles in nitrogen metabolism, stress responses and protein S-nitrosylation in microorganisms under anaerobic conditions^5–15^. HCPs are divided into three classes: I, II and III (Supplementary Fig. 1). Class I HCPs are found in various microorganisms, including bacteria, archaea and unicellular eukaryotes^3–5,10^. Early structural studies on class I HCPs such as *Desulfovibrio vulgaris* HCP (*Dv* HCP) and *Desulfovibrio desulfuricans* HCP (*Dd* HCP) have revealed two metalloclusters: an N-terminal [4Fe-4S] and a central hybrid cluster; the latter is a unique Fe-S-O-type cluster in their as-isolated states^16–21^. A recent structural study of *Methanothermococcus thermolithotrophicus* HCP, an archaeal class I HCP^22^, has also provided information of the common structural features of class I HCPs. Class II HCPs are found only in facultative anaerobes such as *Escherichia coli*^6,7,9,11^, and also have N-terminal [4Fe-4S] and hybrid clusters^23,24^. A critical structural difference between class I and II HCPs is the N-terminal Cys-rich motif. Class II *Ec* HCP shows an N-terminal protrusion important for the function of *Ec* HCP with *E. coli* HCP reductase (HCR), a class II HCP-associating reductase;^7,23^ however, class I *Dv* HCP and *Dd* HCP do not.

Class III HCPs differ from class I/II HCPs in the middle section of their primary amino acid structures (Supplementary Fig. 2)^25^. Approximately 100 amino acids, corresponding to the middle region of class I/II HCPs, are absent, although N- and C-terminal regions and residues for metallocluster ligands are conserved among all HCP classes. Lack of this region in class III HCPs may help understand the structural evolution of HCPs. Notably, class III *Pyrococcus furiosus* HCP (*Pf* HCP) is a homodimer with [4Fe-4S] and hybrid clusters; however, its crystal structure is unavailable^25^.

HCPs exhibit structural homology (with 15–20% amino acid sequence identity) with Ni, Fe-carbon monoxide dehydrogenases (CODHs), which catalyse reversible conversion of CO_2_ to CO (Supplementary Fig. 3)^26,27^. However, their functions, metallocluster active sites and metallocluster ligand sets are different. Instead of the hybrid cluster found in HCPs, CODHs have a unique Ni-Fe-S-type metallocluster ([Ni-4Fe-4S] cluster), called C-cluster^28–30^. Interestingly, all CODHs are homodimers^30–50^, whereas class I/II HCPs are monomers and class III HCPs are homodimers^16–21,23^. The different oligomerization states of HCPs and CODHs are critical for discussing the structural diversity and evolution of the HCP/CODH superfamily (Supplementary Fig. 4). The three-dimensional structures of CODHs and HCPs suggest that these enzymes are more similar than expected based on their amino acid sequences. The overall structural folds of the CODH catalytic domains (monomeric subunits harbouring the C-cluster) and class I/II HCPs can be well-superimposed^21^. Furthermore, the metallocluster-binding sites of CODHs and HCPs are located at equivalent positions, i.e. the Ni-Fe-S-type cluster (C-cluster) for CODHs and Fe-S-O-type cluster (hybrid cluster) for HCPs, despite differences in their cluster types. This indicates that CODHs and HCPs originate from the same ancestor and belong to the same protein superfamily: the HCP/CODH superfamily. However, the structure-based evolutionary relationship between CODHs and HCPs remains unverified.

We herein studied X-ray crystal structure of class III *Methanothermobacter marburgensis* HCP (*Mm* HCP) for structural comparison of it with class I/II HCPs and CODHs to gain critical insights into the structural diversity and evolution of the CODH/HCP superfamily. Furthermore, we studied several *Mm* HCP variants with a Cys67 mutation showing the N-terminal denaturation with keeping this homodimeric architecture, and performed phylogenetic tree analysis.

## Results and discussion

### Structure of class III *Mm* HCP

**Fig. 1:**
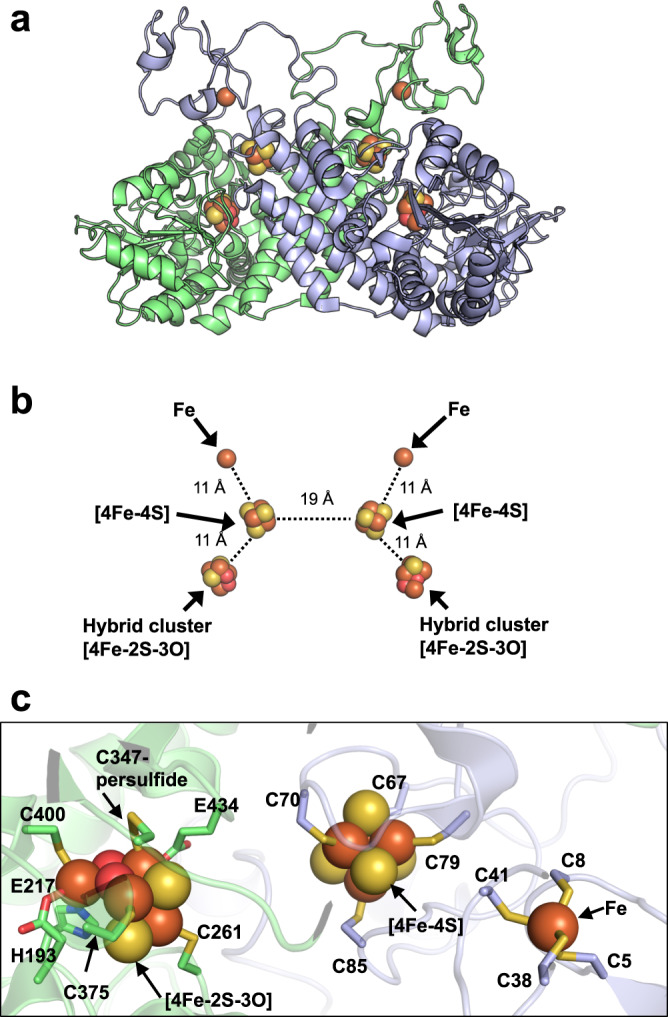
Structure of class III *Methanothermobacter marburgensis* HCP (*Mm* HCP). **a** Overall structure. Each polypeptide of *Mm* HCP is shown in green or light blue. The Fe ions, [4Fe-4S] cluster and hybrid cluster ([4Fe-2S-3O] cluster) are represented as spheres. The Fe, S and O atoms of the metallocentres are coloured in brown, yellow and red, respectively. **b** View of the alignments of the metal cofactors of *Mm* HCP in the same orientation as the overall structure in Fig. 1a. Dashed lines indicate the distances between metallocentres. **c** Close-up view of the metal-binding sites of *Mm* HCP.

We determined the X-ray crystal structure of *Mm* HCP at 2.8 Å resolution (Fig. 1). *Mm* HCP exhibited a homodimeric architecture (Fig. 1a), unlike other HCP classes (Supplementary Fig. 1). In this architecture, each *Mm* HCP protomer was considered as three domains: rubredoxin, [4Fe-4S] cluster-binding domain and HCP domain. The rubredoxin domain is connected to the [4Fe-4S] cluster-binding region via a flexible polypeptidyl linker, as indicated by its weak electron density (Supplementary Fig. 5). Sequence comparison of class III HCPs revealed that the rubredoxin domain is not present in all class III HCPs but is only observed in class III HCPs from *Methanobacteriales* such as *M. marburgensis* and *M. thermautotrophicu*s (Supplementary Fig. 2). In contrast, the HCP domain containing the hybrid cluster is commonly observed in all class I/II/III HCPs.

These metallocentres were symmetrically aligned from the dimer interface of *Mm* HCP (Fig. 1b). In one side of the aligned symmetric clusters, the distances between the Fe site and [4Fe-4S] and [4Fe-4S] and hybrid clusters were reasonable for electron transfer between the metallocentres in one side. However, the distance between two [4Fe-4S] clusters was longer than that of the metalloclusters (i.e. Fe of the rubredoxin domain, [4Fe-4S] and hybrid cluster of the HCP domain) in one side, suggesting that electron transfer in one side is more favourable than that between two HCP domains via the interface.

The hybrid cluster and ligands of *Mm* HCP were carefully considered by comparing the electron density maps (Supplementary Fig. 6). For structural modelling, the electron density map around the Cys-persulfide ligands provides clues to help assign the hybrid cluster to an oxidised form ([4Fe-2S-3O])^18,21^ rather than a reduced form ([4Fe-3S])^20^, because only Cys-persulfide can be sufficient for the appropriate coordination distance to Fe. Therefore, Cys347-persulfide, rather than Cy347-thiolate, was well-modelled to the hybrid cluster based on the distance and geometry between Cys-persulfide and Fe in the hybrid cluster, similar to the structural refinement of class II *Ec* HCP at low resolution (Supplementary Fig. 7)^23^. Indeed, modelling [4Fe-2S-3O] with Cys347-persulfide in *Mm* HCP minimised electron density error and decreased *R*/*R*_free_ values, confirming that the refined structure of *Mm* HCP is the as-isolated form with a [4Fe-2S-3O] cluster^23^. Thus, the ligands for the [4Fe-2S-3O]-type hybrid cluster of *Mm* HCP were confirmed as Cys-persulfide, Cys, His and Glu ligands, equivalent to those of the other HCP classes in the [4Fe-2S-3O] cluster-bound state (Fig. 1c and Supplementary Fig. 7).

**Fig. 2:**
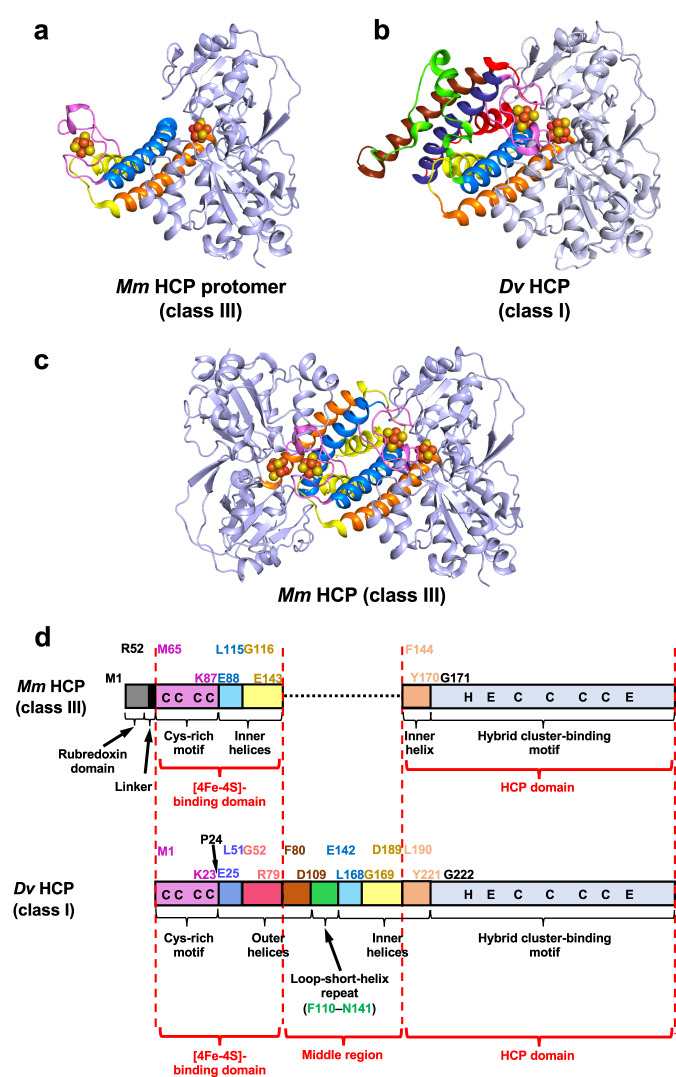
Comparison of class III *Mm* HCP and class I *Dv* HCP. **a** Structure of one protomer of class III *Mm* HCP **b** Overall structure of *Dv* HCP showing its monomeric architecture. **c** Overall structure of *Mm* HCP showing its homodimeric architecture. **d** Schematic representation of the primary structures of class III *Mm* HCP and class I *Dv* HCP for comparing domain structures. The regions in the same colours are corresponding regions at equivalent positions in both class III *Mm* HCP and class I *Dv* HCP, i.e. Cys-rich regions (Met65–Lys87 of *Mm* HCP and Met1–Lys23 of *Dv* HCP) in magenta; inner helices (Glu88–Leu115 of *Mm* HCP and its corresponding region, Glu142–Leu168 of *Dv* HCP, in cyan; Gly116–Glu143 of *Mm* HCP and its corresponding region, Gly169–Asp189, of *Dv* HCP in yellow and Phe144–Tyr170 of *Mm* HCP and its corresponding region, Leu190–Tyr221, of *Dv* HCP in light orange). Three outer helices of *Dv* HCP show amino acid sequence similarity to three inner helices, i.e. the cyan inner helix is similar to the blue outer helix, Glu25–Lys51 of *Dv* HCP; the yellow inner helix is similar to the red outer helix, Gly52–Arg79 of *Dv* HCP and the light orange inner helix is similar to the brown outer helix, Phe80–Asp109 of *Dv* HCP in terms of the amino acid sequences. The green helix–loop–helix region (loop-short-helix repeat), Phe110–Asn141, linking one outer helix (brown) and one inner helix (light orange) in *Dv* HCP is not similar to any regions of *Mm* HCP, implying that this region was additionally fused during the molecular evolution of class III HCP to class I/II HCPs, although the origin of this region is unknown at present. The black dashed line from Glu143 to Phe144 of *Mm* HCP in Fig. 2d represents the gap region when the amino acid sequences of the different HCP classes are aligned in Supplementary Fig. 2. The letters, C, H, and E, in the schematic primary structures indicate conserved Cys, His and Glu, respectively.

By focusing on one side of the symmetric axis at the homodimer centre, we identified a unique structural feature of the *Mm* HCP homodimeric architecture: the rubredoxin and [4Fe-4S] cluster-binding domains are derived from one protomer (i.e. Met1–Arg52 for the rubredoxin domain, Gly53–Asp64 for the linker and Met65–Tyr170 for the [4Fe-4S]-binding domain), whereas the HCP domain is from another protomer (i.e. Gly171–C-terminal end) (Fig. 1a). These two regions, the [4Fe-4S]-binding and HCP domains from different protomers, exhibit monomeric HCP-like folding (Fig. 2 and Supplementary Fig. 1). This feature was observed in the superimposed structures of class III *Mm* HCP and class I/II HCP (Supplementary Fig. 8). In this superimposition, we observed that the [4Fe-4S] and hybrid clusters are superimposed and present at equivalent positions, suggesting that the manner of metallocluster alignment is important for HCP functions, despite the different oligomeric states between class I/II and III HCPs.

### Structural comparison of the three HCP classes

We compared class III *Mm* HCP protomer (Fig. 2a) with class I *Dv* HCP (Fig. 2b), a monomeric HCP, to understand why and how class III is a homodimer and class I/II HCPs are monomers. By comparing the primary and three-dimensional structures (Supplementary Figs. 2, 8) and topologies (Supplementary Fig. 9) of HCPs, we observed some unique features in their domain structures (Fig. 2), possibly providing clues regarding the structural evolution from class III to class I/II HCPs. In class III *Mm* HCP, three inner helices (indicated as cyan, yellow and orange-coloured regions in Fig. 2a, c, d) are involved in the dimer interface (Fig. 2c). However, the topologies of class I *Dv* HCP (Fig. 2b) and the protomer of class III *Mm* HCP (Fig. 2a) are different. The outer helices (indicated as blue, red and brown helices in Fig. 2b, d) are only present in class I *Dv* HCP. If these outer helices were present in class III HCPs, dimerisation was prevented. Moreover, by carefully comparing the regions of the outer helices ranging from Glu25 to Leu51 (in dark blue), Gly52 to Arg79 (in red) and Phe80 to Asp109 (in brown) (Fig. 2d), the amino acid sequences of these and the inner regions of *Mm* and *Dv* HCPs were found to be similar (Supplementary Fig. 10). Specifically, the blue-, red- and brown-coloured regions are similar to the cyan-, yellow- and orange-coloured regions, respectively (Fig. 2d). Therefore, the outer helices of class I HCPs may be generated via gene duplication of the inner helices of class III HCPs. Owing to similarities in class I and II HCPs, all monomeric HCPs (i.e. class I and II HCPs) may originate from class III HCPs via gene duplications. One inner helix (in orange) is a part that binds to the hybrid cluster-binding motif, resulting in the HCP domain. Nevertheless, amino acid sequence alignments of class I, II and III HCPs (Supplementary Fig. 2) did not reveal that gene duplication is related to HCP evolution because no three-dimensional structures had been available for class III HCPs. Therefore, the present study on the homodimeric architecture of *Mm* HCP allows us to focus on more precisely distinguishing the domain structures of HCPs, which is meaningful for understanding HCP evolution (Fig. 2d). Moreover, the HCP domain (one orange-coloured inner helix + hybrid cluster-binding domain) remains homologous among all HCP classes, exhibiting seven conserved ligands for [4Fe-2S-3O] cluster binding (Supplementary Figs. 7, 8).

As an exceptional region showing no clear sequence similarity to the other regions of class III *Mm* HCP, a green-coloured loop–short–helix region (loop-short-helix repeat) was identified in class I *Dv* HCP (Fig. 2b, d). This region links one outer helix (in brown) and one inner helix (in cyan), indicating that it is unique to monomeric HCPs.

To reliably predict the structures of other class III HCPs via homology modelling, homodimeric architecture-crashing regions (i.e. outer helices and the loop–short–helix region) should be removed from the template HCP model, although structural modelling of class III *Pf* HCP was previously performed using class I *Dv* HCP with such dimerisation-preventing regions^25^. Availability of the *Mm* HCP homodimeric structure suggests that we can reconsider the homology model of class III *Pf* HCP (Supplementary Fig. 11). In the class I *Dv* HCP-based model of *Pf* HCP, the residue Leu96-Met106 formed a long loop connecting two helices. However, this loop was not observed in class III *Mm* HCP. However, in the class III *Mm* HCP-based model of *Pf* HCP, the residue Leu96-Met106 formed an α-helix corresponding to one of the inner helices at the dimer interface. Therefore, the X-ray crystal structure of class III HCP is important for developing more reliable structural models for other class III HCPs.

### Rubredoxin domain

**Fig. 3:**
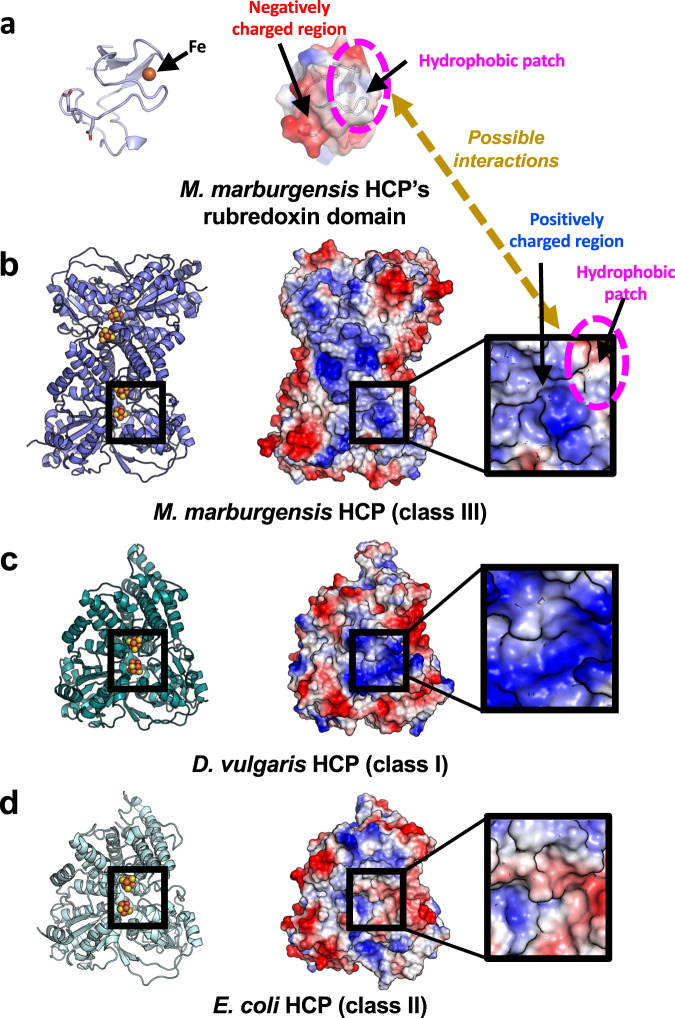
Surface comparison of the rubredoxin domain of *Mm* HCP and each HCP class. **a** The rubredoxin domain. **b** *Mm* HCP (without rubredoxin domain). **c** *Dv* HCP. **d** *Ec* HCP. The blue and red coloured surfaces indicate the positively and negatively charged regions, respectively. The white-coloured surface indicates the hydrophobic region. The box in the thick black line indicates the close-up views around the N-terminal [4Fe-4S] cluster, which could mainly serve as an electron-accepting cofactor from outside. Fe, [4Fe-4S] and hybrid clusters are shown in spheres. The Fe, S and O atoms are coloured in brown, yellow and red, respectively.

The N-terminal region, including the rubredoxin domain of *Mm* HCP, can provide insights regarding differences between class III HCPs and other classes. The rubredoxin domain of *Mm* HCP was purified and characterised via UV–visible and electron paramagnetic resonance (EPR) spectroscopy (Supplementary Fig. 12); the spectroscopic properties of this domain were similar to typical rubredoxin, which can be isolated as a small electron transfer protein^51–53^. The surface charge distribution and shape of the rubredoxin domain are suitable for its binding to and [4Fe-4S] cluster-binding region of class III *Mm* HCP (Fig. 3a). For example, the negatively charged area of the rubredoxin domain could bind to the positively charged area of the surface beside the [4Fe-4S] cluster via electrostatic interactions. The locations of hydrophobic patches of the surface of the rubredoxin domain and the [4Fe-4S] cluster-binding region were also favourable for binding. Indeed, the crystal structure of *Mm* HCP revealed that the rubredoxin domain is located close to the [4Fe-4S] cluster-binding domain, in a distance between the Fe site and [4Fe-4S] cluster, for possible electron transfer. Furthermore, the surface charge of the [4Fe-4S] cluster-binding region of class III *Mm* HCP is distinct from that of class I *Dv* HCP and class II *Ec* HCP. Class I *Dv* HCP exhibited a negatively charged surface, whereas class II *Ec* HCP exhibited a hydrophobic surface with protrusion of its Cys-rich region, as described previously^23^. Interestingly, the surface charges of class III and I HCPs were also different (Supplementary Fig. 2), suggesting that their electron transfer partner proteins are different, though the partners for class I and III HCPs remain unknown.

Surface charge differences in distinct HCP classes provide insights regarding partner proteins. For example, an interesting hypothesis is that rubredoxin is a partner for class III HCPs without the rubredoxin domain fusion. However, how does electron transfer occur in class III HCPs with rubredoxin (domain)? The redox potential of Fe in rubredoxin usually ranges from −50 to +50 mV, whereas that of a 4Cys-coordinating [4Fe-4S] cluster ranges from −400 to –300 mV^53^. The redox potentials of the hybrid cluster tend to be positive (−220 to +80 mV)^1^ compared with the usual potential (−400 to −300 mV) of a 4Cys-coordinating [4Fe-4S] cluster. Therefore, favourable electron flow may be from the [4Fe-4S] cluster to the hybrid cluster and/or rubredoxin if rubredoxin is bound or fused to class III HCPs. In such cases, a favourable electron flow for HCP function may be as follows: the [4Fe-4S] cluster directly accepts an electron from an unknown physiological partner (except for rubredoxin) and then transfers it to the hybrid cluster, similar to the class II HCP–HCR system with NADH^23^. If such an electron flow mainly occurs, a physiological meaning of rubredoxin is unclear; however it will be of interest to consider a physiological partner of class III HCPs.

### Comparison of the protein architectures of class III *Mm* HCP and CODHs

**Fig. 4:**
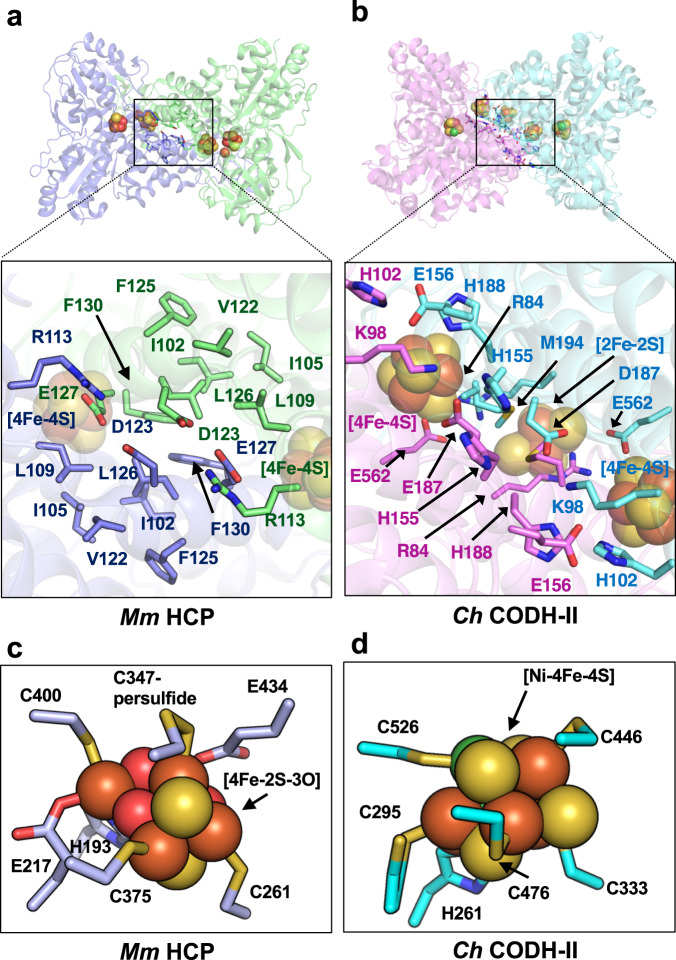
Comparison of *Mm* HCP with *Ch* CODH-II. Overall structure and homodimeric interface of **a** *Mm* HCP and **b** *Ch* CODH-II (PDB ID: 3B51 [https://www.rcsb.org/structure/3B51])^35^. **c** Hybrid cluster-binding site of *Mm* HCP. **d** C-cluster-binding site of *Ch* CODH-II. Fe, [4Fe-4S] cluster, hybrid cluster and C-cluster are shown as spheres. The Fe, Ni, S and O atoms are coloured in brown, green, yellow and red, respectively. Residues at the interfaces are represented as stick models.

To explore the structural relationships between class III HCPs and CODHs, the structures of class III *Mm* HCP and *Carboxydothermus hydrogenoformus* CODH-II (*Ch* CODH-II) were compared using structural superimposition (Fig. 4). The overall homodimeric architecture and many helices and sheets of *Mm* HCP and *Ch* CODH-II were observed at equivalent locations in the superimpositions, suggesting that these two proteins are structurally homologous. However, some CODH-specific loops or helices were primarily observed in the surface regions (Supplementary Fig. 13), resulting in the longer length of the CODH polypeptide than of class III *Mm* HCP polypeptide. Such loops and helices and dimer interfaces exhibited distinct properties. Moreover, *Mm* HCP exhibited many hydrophobic residues at the interface, whereas *Ch* CODH-II exhibited more hydrophilic residues. In *Mm* HCP, many hydrophobic residues were clustered for maintaining the homodimeric architecture via hydrophobic interactions. Furthermore, a salt bridge between Arg113 and Glu127 helped maintain the homodimeric architecture inside the hydrophobic core of the interface. In contrast, some polar residues rather than one salt bridge were observed beside hydrophobic residues in the dimer interface of *Ch* CODH-II, suggesting that the homodimeric architecture of CODH depends on more polar interactions compared to *Mm* HCP. Interface hydrophobicity/hydrophilicity, which contributes to the dimeric architecture, is also a good signature to distinguish class III HCP and CODHs.

Additionally, the absence/presence of an iron-sulphur (Fe-S) cluster at the dimer interfaces of *Mm* HCP and CODHs were different (Figs. 1, 4 and Supplementary Figs. 1, 3). The Fe-S cluster at the dimer interface was not observed in *Mm* HCP, whereas CODHs had an interfacial [2Fe-2S]^44^ or [4Fe-4S] cluster^30,32,35,37,43,44^ (Supplementary Figs. 2, 4), indicating that the interfacial Fe-S cluster is a distinct feature between class III HCPs and CODHs. By viewing both the dimer interfaces and absence/presence of the interfacial Fe-S cluster, we demonstrated that class III HCPs and CODHs may be evolutionarily more distinct than expected based on the overall structure.

### Comparing active sites of *Mm* HCP and CODHs

Comparing the active site architectures of *Mm* HCP and *Ch* CODH-II is of great interest, although metallocluster types are known to differ: [4Fe-2S-3O] in *Mm* HCP and [Ni-4Fe-4S] in *Ch* CODH-II (Fig. 4 and Supplementary Figs. 14, 15). Some residues of *Mm* HCP and *Ch* CODH-II were identical at equivalent positions. Particularly, His193, Cys261, Cys375 and Cys400 of class III *Mm* HCP were located at positions equivalent to His261, Cys333, Cys476 and Cys526 of *Ch* CODH-II. The positions of Cys347-persulfide of *Mm* HCP and Cys446 of *Ch* CODH-II were also superposed, although Cys347 of *Mm* HCP was persulfurated. In contrast, the position of Glu217 of *Mm* HCP was not Glu but Cys295 of *Ch* CODH-II. Moreover, the position of Glu434 was used as the seventh ligand for [4Fe-2S-3O], an as-isolated hybrid cluster, whereas the corresponding position in *Ch* CODH-II was His561, which is not a ligand for [Ni-4Fe-4S]. It should also be noted that His562 of *Ch* CODH-II is not used as a conserved His site among CODHs, e.g. Ser is present in this site instead of His in *D. vulgaris* CODH (*Dv* CODH)^44^ and *Clostridium autoethanogenum* CODH^30^. Considering the equivalent positions of the ligands of HCPs/CODHs, despite some differences in amino acid ligand types, a hypothetical scenario for differences in the ligand sets may have been caused by point mutations during HCP/CODH family evolution.

Interestingly, the [4Fe-2S-3O] cluster ligand set of HCPs is similar to that of *Carboxydothermus hydrogenoformus* CODH-V (*Ch* CODH-V)^48^, which harbours a Fe-S-O-type cluster ([4Fe-2S-2O] + 2H_2_O) rather than the C-cluster (Supplementary Fig. 15). Differences in the ligand sets of *Mm* HCP and *Ch* CODH-V are the positions of Cys-persulfide residues. Another difference is the presence/absence of the bindings of one Glu and one His to Fe: Glu217 and His193 of *Mm* HCP are ligated to Fe, whereas the equivalently positioned Glu295 and His259 of *Ch* CODH-V are not ligated to Fe. (Supplementary Fig. 15). Interestingly, amino acid sequence alignments (Supplementary Fig. 14) showed Glu295 of *Ch* CODH-V is unique only in *Ch* CODH-V. This Glu295 position of *Ch* CODH-V is substituted by the conserved Cys ligand in the other CODHs (e.g. Cys295 in *Ch* CODH-II) with C-cluster and not aligned to the conserved Glu ligand in HCPs (e.g. Glu217 in *Mm* HCP). Thus, *Ch* CODH-V is certainly different from the other types of CODHs and HCPs. Nevertheless, the structural features of *Ch* CODH-V may be more similar to other types of CODHs (e.g., *Ch* CODH-II) than to HCPs because *Ch* CODH-V has the interfacial Fe-S cluster and additional CODH-specific loop structures at its surface in a similar manner to *Ch* CODH-II (Supplementary Fig. 13).

### Structure of the *Mm* HCP C67Y variant

When cloning *Mm hcp*, a spontaneous point-mutated *Mm hcp* was unexpectedly cloned. DNA sequencing revealed that this mutation was a Cys67 to Tyr change, resulting in the codon conversion of TGC to TAC at residue 67. Although the exact reason for this mutation remains unclear; it may have been caused by directly cloning *Mm hcp* using *M. marburgensis* cells (not its pure genomic DNA) as a template in polymerase chain reaction (PCR).

**Fig. 5:**
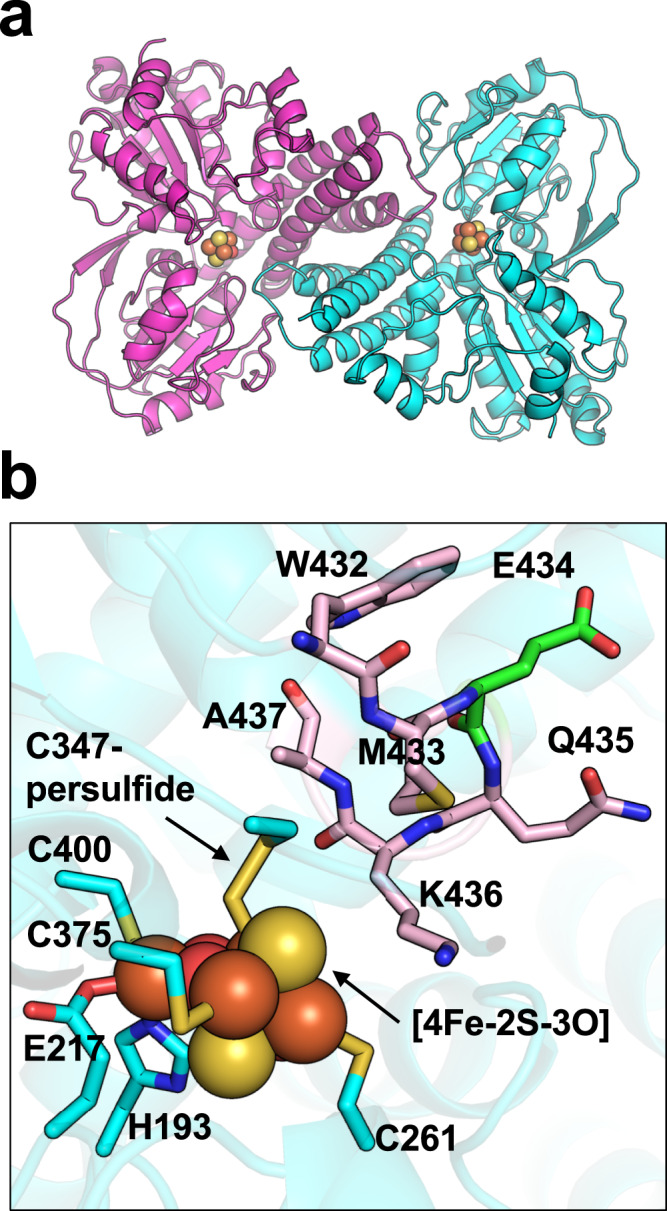
Structure of the *Mm* HCP C67Y variant. **a** Overall structure. The hybrid cluster, which was modelled as a [4Fe-2S-3O] cluster, is represented as spheres. The Fe, S and O atoms are coloured in brown, yellow and red, respectively. None of the [4Fe-4S] clusters or the rubredoxin domain were modelled because there were no observations in the corresponding electron density maps. **b** Hybrid cluster-binding site of the *Mm* HCP C67Y variant. Ligands of the hybrid cluster are coloured in cyan. The surrounding residues (W432–M433 and Q435–A437) are coloured in pink and Glu434 is coloured in green.

This C67Y mutation occurred in one of the Cys ligands of the [4Fe-4S] cluster; *Mm* HCP C67Y variant (*Mm* HCP C67Y) may be of interest when considering the structure–function relationship of *Mm* HCP. Therefore, we expressed, purified and characterised *Mm* HCP C67Y using X-ray crystallography, spectroscopic analysis and the hydroxylamine reductase activity assay. The crystal structure of *Mm* HCP C67Y was determined at 3.0 Å resolution (Fig. 5). This variant exhibited a homodimeric architecture identical to that of *Mm* HCP wild-type (WT) (Fig. 1), demonstrating the rigidity of the dimeric architecture. However, no electron density maps of rubredoxin and the [4Fe-4S] cluster-binding domains of *Mm* HCP C67Y were observed (Supplementary Fig. 16). This indicates that these regions were disordered, which could have been caused by the lack of the Cys ligand at position 67 in the [4Fe-4S] cluster. Despite the presence of the unfolded region, the homodimeric architecture was still maintained in *Mm* HCP C67Y. This indicates that the dimer interface of *Mm* HCP is rigid and not affected by N-terminal region denaturation.

We noted differences in the coordination structure of the hybrid cluster around the Glu434 position, which can be attributed to the C67Y mutation. In *Mm* HCP C67Y, Glu434 was positioned at the solvent-exposed area, far from the hybrid cluster (Fig. 5 and Supplementary Fig. 17), although Glu434 of WT is a ligand of the hybrid cluster (Fig. 1). Superimposing *Mm* HCP WT and C67Y showed that the regions around Glu434 and Trp461 were shifted by ~5.3–5.8 Å (Supplementary Fig. 18). Thus, the C67Y mutation may have induced the loss of the [4Fe-4S] cluster in *Mm* HCP C67Y, possibly causing N-terminal region denaturation. Owing to the denaturation, the loop region around Glu434 became unstabilized and shifted to the solvent area, which may have been favourable because the hydrophilic residues, including Glu434, may compensate for the positions where the [4Fe-4S] cluster-binding domain of WT was occupied, resulting in the stabilisation of this area via networks of polar interactions with solvent waters.

### Spectroscopic and functional characterisation of *Mm* HCP

Spectroscopic and functional characterisations were performed to assess *Mm* HCP WT and the C67Y variant. To determine the effects of Cys67 mutation on *Mm* HCP properties, the C67S and C67A variants were prepared and characterised.

**Fig. 6:**
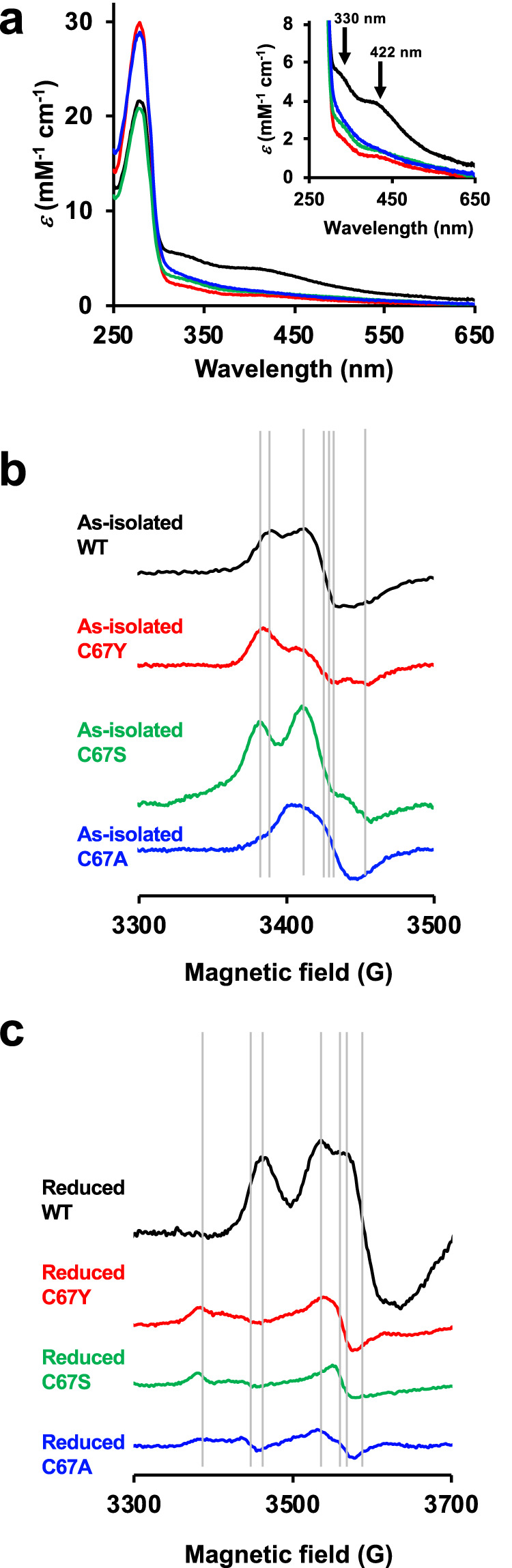
UV–visible and EPR spectra of *Mm* HCP WT and the C67Y, C67S and C67A variants. **a** UV–visible spectra. **b** EPR spectra of the as-isolated *Mm* HCP WT and variants in the magnetic fields 3300–3500G. **c** EPR spectra of the dithionite-reduced *Mm* HCP WT and variants in the magnetic fields 3300–3700G. The spectra of *Mm* HCP WT and the C67Y, C67S and C67A variants are indicated as black, red, green and blue lines, respectively. Inserted grey lines in the EPR spectra allow a comparison of the EPR signals and their *g*-values.

The UV–visible spectrum of the as-isolated forms showed that *Mm* HCP WT contained a high degree of Fe-S-type cofactors, which were found at ~320–450 nm, rather than the other variants (Fig. 6a). Although their Fe-S-type absorptions were of varying degrees, dithionite reduction resulted in the disappearance of Fe-S-type cofactor absorption in *Mm* HCP WT and variants, indicating that these Fe-S-type cofactors are redox-active (Supplementary Fig. 19).

The EPR spectra of the as-isolated forms of *Mm* HCP WT and variants provided information on the hybrid cluster and its environments (Fig. 6b and Supplementary Figs. 20, 21). The EPR spectrum of *Mm* HCP WT around the *g* = 2 region showed signals at *g* = 2.023, 2.009, 2.003, 1.996 and 1.984. When this spectrum was compared with a previously determined EPR spectrum of class III *Pf* HCP^25^, the rhombic signal features of *Mm* HCP WT and *Pf* HCP were similar. By comparing spectral features, the major signals at *g* = 2.023, 2.003 and 1.984 of *Mm* HCP WT could be assigned as the [4Fe-2S-3O]-type hybrid cluster, although these values were not exactly similar to the signals at *g* = 2.010, 1.967 and 1.939 of *Pf* HCP. *Mm* HCP C67Y and C67S also exhibited spectral features similar to those of *Mm* HCP WT, with similar *g*-values. Therefore, these two variants have the [4Fe-2S-3O]-type hybrid cluster or [4Fe-2S-3O]-like metallocluster, as the intensities of the signals were different between the variants and WT. In contrast, *Mm* HCP C67A exhibited different spectral features compared with *Mm* HCP WT, C67Y and C67S. The EPR spectrum of *Mm* HCP C67A was simple and had a broad signal at *g* = 1.999, indicating that *Mm* HCP C67A had no [4Fe-2S-3O]-type hybrid cluster. The hybrid cluster environment was investigated via EPR analysis of the as-isolated HCPs in a low magnetic field (Supplementary Fig. 21). *Mm* HCP WT showed two clear EPR signals at *g* = 9.424 and 6.374, which can be assigned to the [4Fe-2S-3O]-type hybrid cluster, as observed in previous data for the EPR of *Pf* HCP, with two strong signals. In contrast, *Mm* HCP C67Y and C67A exhibited weak EPR signals, but *g*-values were similar to those of WT. *Mm* HCP C67A did not exhibit such signals, particularly at *g* = 9.424, further indicating that this variant has no hybrid cluster.

The EPR spectra of the dithionite-reduced HCPs provided useful information regarding the [4Fe-4S] and hybrid clusters and their environments (Fig. 6c and Supplementary Fig. 22). The EPR spectra of dithionite-reduced *Mm* HCP WT and *Pf* HCP were similar. For example, the signals at *g* = 1.978, 1.940 and 1.910 of *Mm* HCP WT were relevant to those at *g* = 2.013, 1.890 and 1.820, which are attributed to the hybrid cluster. Furthermore, broad signals of *Mm* HCP WT resulted in features similar to *Pf* HCP, which can be interpreted as a spin-admixed *S* = 3/2 ground state of the [4Fe-4S]^+^ cluster, as reported previously^25^. However, the EPR spectra of dithionite-reduced *Mm* HCP C67Y, C67S and C67A and WT were different. First, a small signal at *g* = 2.026 was observed in the variants; however, this was not observed in WT. Second, the much broad EPR signal of the spin-admixed *S* = 3/2 ground state^54^ of [4Fe-4S]^+^, which was found in *Mm* HCP WT (Fig. 6c) and *Pf* HCP^25^ and was overlapping with the signal of the hybrid cluster, was not observed in the variants. Therefore, these variants are unlikely to have the [4Fe-4S] cluster.

To further characterise *Mm* HCP WT and the variants, the hydroxylamine reductase assay was performed at 65 °C (Supplementary Fig. 23)^7,25^, the optimal growth temperature for *M. marburgensis*, for gaining the Michaelis–Menten kinetic parameters (Table 1). *Mm* HCP C67S and C67Y were less active than WT, as they exhibited lower *k*_cat_ and higher *K*_M_ values than WT. Considering similar spectral features of *Mm* HCP C67S and C67Y variants and the structure of *Mm* HCP C67Y, the lower activity was caused by the fact that *Mm* HCP C67S and C67Y variants had neither [4Fe-4S] cluster nor Glu-coordination to the hybrid cluster. Interestingly, a previous study on class II *Ec* HCP E492D, E492A, E492V and E492G variants, with a mutation in Glu492 (equivalent to Glu434 of *Mm* HCP), also demonstrated that this conserved Glu plays an important role in *Ec* HCP functioning^6^, supporting the significance of the Glu ligand for HCPs function. In contrast, *Mm* HCP C67A showed lower *K*_M_ than WT, although *k*_cat_ was the lowest of all. The lowest *k*_cat_ can be understood by the fact that this variant had neither [4Fe-4S] nor [4Fe-2S-3O]-type clusters. Instead, unknown Fe-type cofactor may have been included in this variant as seen in EPR spectra, which may cause the structure of *Mm* HCP C67A was rather different from WT and the other variants and lower *K*_M_.

**Table 1:** Hydroxylamine reductase activity assay using the *Mm* HCP WT and variants

*Mm* HCP	*K*_M_ (mM^−1^)	*k*_cat_ (s^−1^)
Wild-type (WT)	0.89 ± 0.15	2.8 ± 0.2
C67Y variant	2.7 ± 0.4	0.52 ± 0.06
C67S variant	1.6 ± 0.7	0.34 ± 0.05
C67A variant	0.32 ± 0.14	0.073 ± 0.005

### Insights into the diversity and evolutionary relationship between HCPs and CODHs

**Fig. 7:**
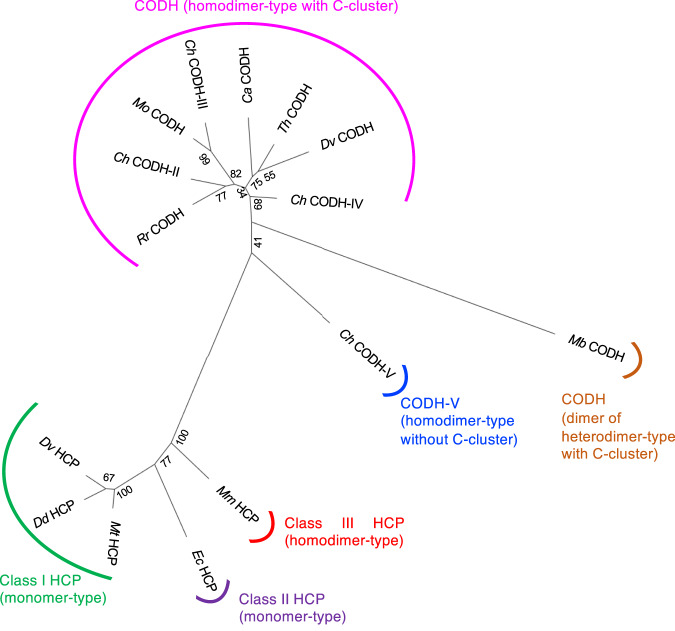
Phylogenetic analysis using three-dimensional structural information. A structure-guided unrooted phylogenetic tree of HCPs and CODHs whose three-dimensional structures are known was constructed. *Mm* HCP, *Methanothermobacter marburgensis* HCP (class III HCP); *Ec* HCP, *Escherichia coli* HCP (class II HCP); *Mt* HCP, *Methanothermococcus thermolithotrophicus* HCP (class I HCP); *Dd* HCP, *Desulfovibrio desulfurican*s HCP (class I HCP); *Dv* HCP, *Desulfovibrio vulgaris* HCP (class I HCP); *Rr* CODH, *Rhodospirillum rubrum* CODH; *Ch* CODH-II, *Carboxydothermus hydrogenoformans* CODH-II; *Mo* CODH, *Moorella thermoacetica* CODH*; Ch* CODH-III, *C. hydrogenoformans* CODH-III; *Ca* CODH, *Clostridium autoethanogenum* CODH; *Th* CODH, *Thermococcus* sp. AM4 CODH; *Dv* CODH, *Desulfovibrio vulgaris* CODH; *Ch* CODH-IV, *C. hydrogenoformans* CODH-IV; *Mb* CODH, *Methanosarcina barkeri* CODH; *Ch* CODH-V, *C. hydrogenoformans* CODH-V. The percentage of the branching trees are indicated at the corresponding branch points. It is noted that *Ch* CODH-V^48^ has neither the C-cluster nor CODH activity but exhibits CODH-type folds rather than HCP-type folds. It is noted that *Mb* CODH is a dimer of heterodimer (α- and ε-domains), but its catalytic core is composed of a homodimer of α-domains in the same manner as other homodimer-type CODHs.

Structure-based phylogenetic analysis of HCPs and CODHs provided meaningful information when considering their evolutionary history. A three-dimensional structure-based phylogenetic tree analysis^55–57^, which better reflects similarities in the positions/coordinates of the amino acids in the polypeptides than primary structures alone, was conducted (Fig. 7 and Supplementary Fig. 24). Phylogenetic analysis revealed that class III *Mm* HCP, not class I/II HCPs, is the most evolutionarily related to CODHs. Class I HCPs were further from class III *Mm* HCP compared with class II *Ec* HCP. This indicates that HCP evolution occurred from class III HCP to class II and then to class I. The direction of phylogeny-based HCP evolution was related to a possible gene duplication event from homodimeric class III to monomeric class I/II HCPs, as discussed in the section of the three-dimensional structural comparison above.

In the CODH clade, *Ch* CODH-V^48^, which has neither the C-cluster nor CODH activity, is the most evolutionarily related to class III *Mm* HCP. This feature can be understood because the ligands of the hybrid cluster of *Ch* CODH-V are rather similar to those of HCPs (Supplementary Fig. 15), although the overall architecture of *Ch* CODH-V is almost identical to that of other CODHs. Among CODHs having the C-cluster and CODH activity, *Dv* CODH is the distantly closest to class III *Mm* HCP. *Dv* CODH has an interfacial [2Fe-2S] cluster, similar to *Ch* CODH-V, but not other CODHs, supporting the evolutionary relationship between *Ch* CODH-V and *Dv* CODH. It is noted that *Methanosarcina barkeri* CODH (*Mb* CODH), which is branched between *Ch* CODH-V and *Dv* CODH in the phylogenetic tree, is a unique CODH among these CODHs in this analysis from the structural viewpoint. Actually, only *Mb* CODH exhibits heterodimer of dimer architecture (catalytic α-subunit + small ε-subunit)^37^ among the analysed CODHs (Supplementary Fig. 3). This feature is relevant to the fact that the branch of *Mb* CODH from the closest node is very long, implying that *Mb* CODH rather than *Dv* CODH may be a descendant. To discuss further details of HCPs/CODHs superfamily, three-dimensional structure-based bioinformatics with more structural data of HCPs/CODHs from various species as well as their amino acid sequences^28,29^ will be necessarily analysed for future.

**Fig. 8:**
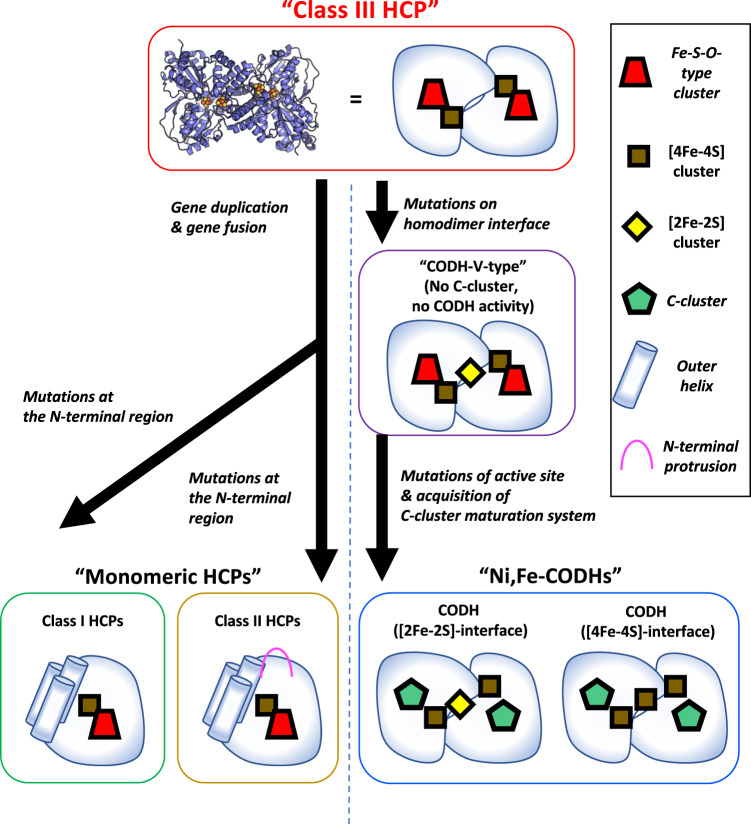
Proposed comprehensive scenario of the molecular evolution of the HCP/CODH superfamily. Monomeric HCPs and CODHs are separated from class III HCP in molecular evolution. In HCP evolution, gene duplication and gene fusion occurred for making outer helices and linker between the inner and outer helices, respectively. Separation of monomeric class I and II HCPs is mainly caused by mutations around the N-terminal domain, including the Cys-rich region, as indicated by the fact that class II HCPs have a unique protrusion of its longer Cys-rich compared with class I HCPs^23^. In CODH evolution, CODH-V first evolved from class III HCP via mutations on the interfaces showing CODH-type features, e.g. presence of the interfacial Fe-S cluster. Then, other classes of CODHs, which have the C-cluster, evolved via mutations on the active site and/or the homodimeric interface and acquisition of the components of the C-cluster maturation system, e.g. Ni-chaperon CooC^45^.

By summarising phylogenetic analysis results, structure and possible gene duplication events, we proposed a comprehensive molecular evolutionary scenario of the HCP/CODH superfamily (Fig. 8). As a common HCP/CODH ancestor protein, class III HCP emerged or was generated from an unknown class III HCP protomer-like protein. Then, in HCP evolution, monomeric HCPs evolved via gene duplication of the inner helices of class III HCPs, resulting in three outer helices. Thereafter, a gene fusion occurred to facilitate helix–loop–helix linking between the inner and outer helices, generating the monomeric HCP architecture. Then, class I/II HCPs were separated to differentiate the N-terminal Cys-rich region and its surroundings, e.g. surface charges in evolution, which are related to the use of different electron transfer partner proteins between classes I and II. Indeed, it is important to identify the unknown electron transfer partners for class I HCPs to assess the separation events of class I/II HCPs for future studies. Meanwhile, in CODH evolution, CODH-V, which has a CODH-type architecture without the C-cluster, evolved from class III HCP via mutations around the homodimeric interfaces such as changes in polar/hydrophobic residues at the interface and incorporation of Cys residues for binding to the interfacial [2Fe-2S] cluster. Then, the C-cluster maturation system could have been acquired by CODH-V with some mutations necessary for harbouring the C-cluster with CODH activity, resulting in CODHs with the C-cluster.

In conclusion, we identified the X-ray crystal structure of class III HCPs using *Mm* HCP and demonstrated that its unique homodimeric architecture is related to CODHs, providing structure-based clues for the hypothetical scenario of the molecular evolution of the HCP/CODH superfamily. Further, we analysed the *Mm* HCP variants with Cys67 mutation, a ligand of the N-terminal [4Fe-4S] cluster, and demonstrated the significance of the N-terminal region for the architecture around the hybrid cluster and catalytic function of HCPs. Considering that three-dimensional structures of all HCP classes—class I HCPs from *D. vulgaris*, *D. desulfuricans* and *M. thermolithotrophicus*, class II HCP from *E. coli* and class III from *M. marburgensis*—are available, they can be used in future structural comparison studies to provide insights regarding the diversity of HCP classes, evolutionary relationships between HCPs and CODHs and interactions between HCPs and their potential partners at the molecular level. The presented structure of class III HCP is a milestone in understanding the origin of the HCP/CODH superfamily, a key enzyme superfamily in the anaerobic biological world.

## Methods

### Materials

Isopropyl-β-D-thiogalactopyranoside (IPTG) was purchased from BLD Pharmatech Inc. (Shanghai, China). Imidazole and dithiothreitol (DTT) were purchased from Wako Pure Chemical Industries (Osaka, Japan). Other chemicals used in this study were purchased from Sigma-Aldrich (St. Louis, MO, USA), Nacalai Tesque (Kyoto, Japan) or Tokyo Chemical Industry Co., Ltd. (Tokyo, Japan). All oligo DNA primers were purchased from Eurofins Genomics Inc. Japan (Tokyo, Japan). *M. marburgensis* was provided by RIKEN BRC through the National BioResource Project of MEXT/AMED, Japan.

### Plasmid construction

The *M. marburgensis* gene encoding *Mm* HCP was amplified via PCR using KOD FX neo (Toyobo, Japan), *M. marburgensis* cells and NdeI-*Mm* HCP-F and SalI-*Mm* HCP-R primers (Supplementary Table 1). The amplified gene was then cloned into the pET21a plasmid (Novagen, Merck Millipore, Burlington, MA, USA) between the NdeI and XhoI sites. The cloned genes were verified via DNA sequencing, suggesting the cloning of the *M. marburgensis hcp* WT and C67Y mutant, which may have been caused by a spontaneous point mutation during the PCR cycle. In particular, it was hypothesised that this occurred because PCR amplification of *Mm hcp* was performed using a crude sample, i.e. *M. marburgensis* cells, rather than pure genomic DNA. The expression plasmids for *Mm* HCP WT and *Mm* HCP C67Y variants, each of which has a C-terminal His_6_-tag, were designated as pET21a-*mmhcp-his* and pET21a-*mmhcp-c67y-his*, respectively.

To construct the expression plasmids for the rubredoxin domain, the *Mm* HCP C67S variant and *Mm* HCP C67A variant, inverse PCR was performed using pET21a-*mmhcp-his* as a template and the mutagenic primers listed in Supplementary Table 1. The amplified PCR products were treated with DpnI at 37 °C for 2 h. The DpnI-treated DNA fragments were ligated and transformed into *E. coli* DH5α. The colonies of the transformants were cultivated and plasmids were extracted. The constructed plasmids (i.e. pET21a-*rubredoxin*, pET21a-*mmhcp-c67s-his* and pET21a-*mmhcp-c67a-his*) were verified via DNA sequencing.

### Expression and purification of *Mm* HCP WT and *Mm* HCP variants

The expression plasmids for *Mm* HCP WT, *Mm* HCP C67Y, *Mm* HCP C67S or *Mm* HCP C67A were used for the transformation of *E. coli* C41(DE3) harbouring the pBBRISC plasmid^58^, which was used for the hyperproduction of Fe-S clusters^59^. The transformed *E. coli* cells were then cultured at 37 °C for 4 h in Luria–Bertani (LB) medium supplemented with 100 μg/mL ammonium iron citrate, 50 μg/mL ampicillin and 20 μg/mL gentamycin. When the optical density at 600 nm (OD_600_) reached 0.8–1.0, ITPG was added to the culture at a final concentration of 1 mM. The culture was then sealed under anaerobic conditions, followed by further cultivation at 20 °C for 20 h. Thereafter, the cells were harvested by centrifuging the samples at 4 °C and 9000 × *g* for 20 min. The harvested cells were frozen in liquid nitrogen and stored at −80 °C until further use.

*Mm* HCP WT or each of the variants was anaerobically purified in a Coy Chamber (Coy Laboratory Products Inc., Grass Lake, MI, USA) under an atmosphere of 95% N_2_/5% H_2_ as in the same procedure for purification of *E. coli* HCP^23,24^. The harvested *E. coli* cells were resuspended in buffer A (50 mM Tris–HCl buffer, pH 7.8, 500 mM KCl and 1 mM DTT), disrupted by sonication on ice and then centrifuged at 4 °C and 20,000 × *g* for 40 min. The resulting supernatant was loaded onto a HisTrap FF crude column (Cytiva, Tokyo, Japan) and equilibrated with buffer A. After the column was washed with buffer A, proteins bound to the column were eluted with buffer B (50 mM Tris–HCl buffer, pH 7.8, 500 mM KCl, 1 mM DTT and 250 mM imidazole). The eluted fractions were concentrated using a 50-kDa cutoff Amicon Ultra-15 (Merck KGaA, Darmstadt, Germany). The concentrated fractions were then loaded onto a Sephacryl S-200 16/60 gel filtration column (Cytiva) equilibrated with buffer C (50 mM Tris–HCl, pH 7.8, 150 mM NaCl and 1 mM DTT). The *Mm* HCP fractions were then pooled and concentrated for further use.

### Expression and purification of rubredoxin domain of *Mm* HCP

*E. coli* C41(DE3) cells transformed with the pET21a-*rubredoxin* plasmid were cultured at 37 °C for 4 h in LB medium supplemented with 100 μg/mL ammonium iron citrate and 50 μg/mL ampicillin. IPTG was added to the culture at a final concentration of 1 mM once the optical density at 600 nm (OD_600_) reached 0.8–1.0. The cells were subsequently cultured at 20 °C for 20 h and harvested by centrifugation at 4 °C and 9000 × *g* for 20 min. The harvested cells were then frozen in liquid nitrogen and stored at −80 °C until further use.

The rubredoxin domain was aerobically purified at 4 °C or on ice. *E. coli* cells expressing the rubredoxin domain were disrupted by sonication on ice and then centrifuged at 4 °C and 20,000 × *g* for 40 min for preparation of the supernatant. Then, the supernatant was heated at 65 °C for 20 min and then centrifuged in the same conditions as described above. After heating, the resulting supernatant was concentrated using a 3-kDa cutoff Amicon Ultra-15 (Merck KGaA). The concentrated rubredoxin domain-containing solution was loaded onto a Sephacryl S-100 16/60 gel column (Cytiva) and equilibrated with buffer C. Red-brown fractions containing the rubredoxin domain were pooled and concentrated for further use.

### Measurement of the UV–visible spectra

UV–visible spectra of *Mm* HCP WT and the variants in as-isolated and dithionite-reduced forms were anaerobically recorded in the Coy chamber. The dithionite-reduced forms of *Mm* HCP WT and the variants were prepared by adding 1 mM sodium dithionite to the as-isolated *Mm* HCP WT and different variants. Protein concentration of each of *Mm* HCP WT and the variants for UV–visible spectroscopy was 40 μM, which was determined by Bradford method. The UV–visible spectrum of the rubredoxin domain was aerobically recorded. Protein concentration of the rubredoxin domain for UV–visible spectroscopy was 14 μM. For all the measurements, a NanoPhotometer C40 UV–visible spectrophotometer was used with a quartz cuvette with a 1 cm path.

### Measurement of the EPR spectra

The EPR spectra of *Mm* HCP WT, *Mm* HCP C67Y variant, *Mm* HCP C67S variant and *Mm* HCP C67A variant in the as-isolated forms were recorded on a Bruker E500 (Bruker) equipped with an Oxford ESR900 cryostat (Oxford Instruments, Abingdon-on-Thames, UK) at 20 K. The EPR spectra of the reduced states of *Mm* HCP WT, *Mm* HCP C67Y variant, *Mm* HCP C67S variant and *Mm* HCP C67A variant with 10 mM sodium dithionite were recorded at 12 K. Protein concentrations were 4 mM for WT and 2 mM for the variants in both as-isolated and dithionite-reduced states. The EPR spectrum of the rubredoxin domain in an as-isolated state was recorded at 20 K. The following parameters were used for EPR measurements: microwave frequency, 9.6 GHz; microwave power, 20 mW; modulation amplitude, 6.0 G and modulation frequency, 100 kHz. Protein concentration of the as-isolated rubredoxin domain for EPR measurement was 0.28 mM.

### Hydroxylamine reductase activity assay

The reaction mixture comprised 1.4 μM *Mm* HCP WT, 2.0 μM *Mm* HCP C67Y variant, 9.2 μM *Mm* HCP C67S variant or 7.1 μM *Mm* HCP C67Yvariant, which were determined with Bradford method, and 0.050 mM reduced methyl viologen (MV), which was prepared using 10 mM reduced MV stock solution and 0–5 mM NH_2_OH in 100 mM 2-(*N*-cyclohexylamino)ethanesulfonic acid–NaOH. The hydroxylamine reductase activity of *Mm* HCP was evaluated using data from a range of NH_2_OH concentrations (0–5 mM). The reaction was performed at pH 9.0 and 65 °C, which is the optimal growth temperature for *M. marburgensis*, in an anaerobic Coy chamber under an atmosphere of 95% N_2_/5% H_2_. The change in absorption at 600 nm derived from reduced MV was monitored on the NanoPhotometer C40 UV–visible spectrophotometer using a quartz cuvette with 1 cm path. Differences in the absorbance at 600 nm (ΔAbs_600_) were used for calculating the consumption of the reduced MV in a similar way to the previous study^25^. The molar extinction coefficient *ε*_600_ = 13 mM^−1^ cm^−1^ for reduced MV was used for the calculation of the consumption of the reduced MV^20^. The initial rate of this consumption was plotted against the concentrations of hydroxylamine as a substrate. The plots were used for non-linear least squares curve-fitting using the Michaelis–Menten equation, resulting in the calculation of *k*_cat_ and *K*_M_ values. Curve-fitting was performed using Igor Pro 8.0 software (WaveMetrics, Inc., Lake Oswego, OR. USA). Error bars represent standard deviation. The reactions at each time point were conducted in at least triplicate (*n* = 3) for each reaction composition.

### Crystallisation of *Mm* HCP WT and the C67Y variant

Crystallisation of *Mm* HCP WT and *Mm* HCP C67Y variant was anaerobically performed at 20 °C using the sitting-drop vapour diffusion method in the Coy chamber. For the crystallisation of *Mm* HCP WT, a 1 μL aliquot of 18 mg/mL *Mm* HCP WT was mixed with a 1 μL aliquot of the crystallisation reservoir solution composed of 30% (w/v) PEG4000, 0.1 M Tris-HCl, pH 8.5, and 200 mM sodium acetate. For the C67Y variant, a 1 μL aliquot of 27 mg/mL *Mm* HCP C67Y was mixed with a 1 μL aliquot of the crystallisation reservoir solution composed of 0.05 M HEPES–NaOH, pH 7.0, 0.1 M ammonium acetate, 0.02 M MgCl_2_ and 5% (w/v) PEG8000. The crystallisation conditions are summarised in Supplementary Table 2. Crystals of WT and the C67Y *Mm* HCP variant were obtained within 3 months.

### X-ray data collection and refinement

Crystals were flash-frozen with liquid nitrogen inside the anaerobic Coy chamber. In freezing crystals, no additional cryo-protectants were used. The way of the flash-freezing was as follows:^24^ a microscope and liquid nitrogen in a small dewar flask were placed inside the anaerobic Coy chamber. At this time, the inner pressure of the anaerobic Coy chamber was partially reduced via vacuuming to avoid blowout of the chamber due to vaporising liquid nitrogen. A crystal of interest was picked up with a cryo-loop (CrystalCap SPINE HT, Hampton Research, CA, USA) under the microscope, followed by immersing the crystal into the liquid nitrogen as soon as possible. Then, a plastic cap was attached to the crystal-mounted loop in liquid nitrogen. Finally, the crystal in liquid nitrogen in the dewar flask was taken out from the anaerobic chamber.

The frozen crystals were screened using X-ray diffraction at 100 K on beamlines PF-5A, PF-17A and AR-NW12A at the Photon Factory (Tsukuba, Japan) and the Osaka University beamline BL44XU at SPring-8 (Hyogo, Japan). Datasets were then collected at 100 K on the beamline AR-NW12A for WT with X-ray at a wavelength of 1.739 Å and beamline BL-17A for the C67Y variant with X-ray at a wavelength of 1.000 Å at Photon factory. The datasets were processed using XDS^60^. Resolution was considered by referring to the values of CC_1/2_ > 0.7 and *I*/σ_*I*_ > 2.0 in the highest resolution shell of WT and C67Y data. Molecular replacement was performed using Molrep^61^, with *D. vulgaris* HCP (PDB ID: 1W9M)^21^ and *P. furiosus* rubredoxin (PDB ID: 1BRF)^51^ as the search models. The models were further modified using Coot^62^ and refined using Refmac5^63^ in the CCP4 suite and phenix-refine^64^ in the Phenix suite using non-crystallographic symmetry (NCS) restraints^65^. For the intimal stage of the refinement, simulated annealing was conducted using Phenix to remove model bias. Translation-libration-screw (TLS) refinement was also performed at the final stage of the refinement in Phenix. The final structural models of *Mm* HCP WT and C67Y were validated using MolProbity^66^, showing Ramachandran statistics for WT (favoured regions = 93.4%, allowed regions = 6.12%, outlier regions = 0.47%) and for the C67Y variant (favoured regions = 94.7%, allowed regions = 4.51%, outlier regions = 0.75%). Rotamer outliers were 4.5% for WT and 1.0% for the C67Y variant. Crystal structure figures were created using open-source PyMOL (version 1.7, Schrödinger, LLC). Data collection, refinement statistics and the PDB ID of *Mm* HCP WT and the C67Y variant are given in Supplementary Table 3.

### Bioinformatics

Amino acid sequence alignments were performed using Clustal Omega^67^. The alignment figures were prepared using ESPript3^68^. Schematic diagrams of the protein topologies were created using PDBSum^69^. Three-dimensional structure-guided alignments and phylogenetic analysis of HCPs and CODHs were performed using PROMALS3D^55^ as in a similar way as the structure-guided phylogenetic analysis of metallo-β-lactamases^56^. The structurally characterised CODHs and HCPs used for this phylogenetic analysis were searched using Dali server^70^. Then, the searched 10 structures of CODHs and 5 structures of HCPs were used for superimposition of the structures (Dali’s Z-score: 38–45 for HCPs; 16–26 for CODHs). The amino acid sequence corresponding to the rubredoxin domain of *Mm* HCP was deleted for preparing the phylogenetic tree, because this domain was non-superimposed to the other CODHs and HCPs. The middle region only found in class I and II HCPs and not in class III *Mm* HCP was not deleted in amino acid sequence alignments to retain the structural features of class I/II HCPs in the phylogenetic analysis. Finally, non-rooted phylogenetic tree was generated using the maximum likelihood method implemented in MEGA 11^71^ using the JTT matrix-based model with 1000 bootstrap replicates. Each percentage of trees in which associated taxa clustered is shown at the branching point. The abbreviated taxa are as follows: *Mm* HCP, *M. marburgensis* HCP; *Ec* HCP, *E. coli* HCP; *Mt* HCP, *M. thermolithotrophicus* HCP; *Dd* HCP, *D. desulfuricans* HCP; *Dv* HCP, *D. vulgaris* HCP; *Rr* CODH, *Rhodospirillum rubrum* CODH; *Ch* CODH-II, *C. hydrogenoformus* CODH-II; *Mo* CODH, *Moorella thermoacetica* CODH; *Ch* CODH-III, *C. hydrogenoformus* CODH-III; *Ca* CODH, *Clostridium autoethanogenum* CODH; *Th* CODH, *Thermococcus* sp. AM4 CODH; *Dv* CODH, *D. vulgaris* CODH; *Ch* CODH-IV, *C. hydrogenoformus* CODH-IV; *Mb* CODH, *M. barkeri* CODH α-subunit; *Ch* CODH-V, *C. hydrogenoformus* CODH-V. Homology modelling of the homodimeric and monomeric models of class III *Pf* HCP was performed using the Swiss model^72^, with the structures of *Mm* HCP WT and *Dv* HCP as starting template models, respectively. All figures of the proteins were created using open-source PyMOL (version 1.7, Schrödinger, LLC). Surface charge distributions of HCPs were calculated using APBS^73^ implemented in PyMOL. Superimposition of protein structures was conducted in PyMOL.

### Reporting summary

Further information on research design is available in the Nature Portfolio Reporting Summary linked to this article.

### Supplementary information

Supplementary Information

Reporting Summary

## Data Availability

Structures of *Mm* HCP WT and the C67Y variant are available in the Protein Data Bank (PDB) under the accession codes 7E0L (*Mm* HCP WT) and 7WSX (*Mm* HCP C67Y variant). Reference structures used in this work are available in the Protein Data Bank (PDB) under the accession codes; 1W9M (*Dv* HCP), 1BRF (*Pf* rubredoxin) and 3B51 (*Ch* CODH-II). Source data are provided as a Source Data file.
