# Supplementary material for: Class III hybrid cluster protein homodimeric architecture shows evolutionary relationship with Ni, Fe-carbon monoxide dehydrogenases
Source: Nat Commun. 2023 Sep 14;14:5609. doi: 10.1038/s41467-023-41289-4 (PMC10502027; doi:10.1038/s41467-023-41289-4)
Supplement: Supplementary file 1 — Supplementary Information [file 41467_2023_41289_MOESM1_ESM.pdf]

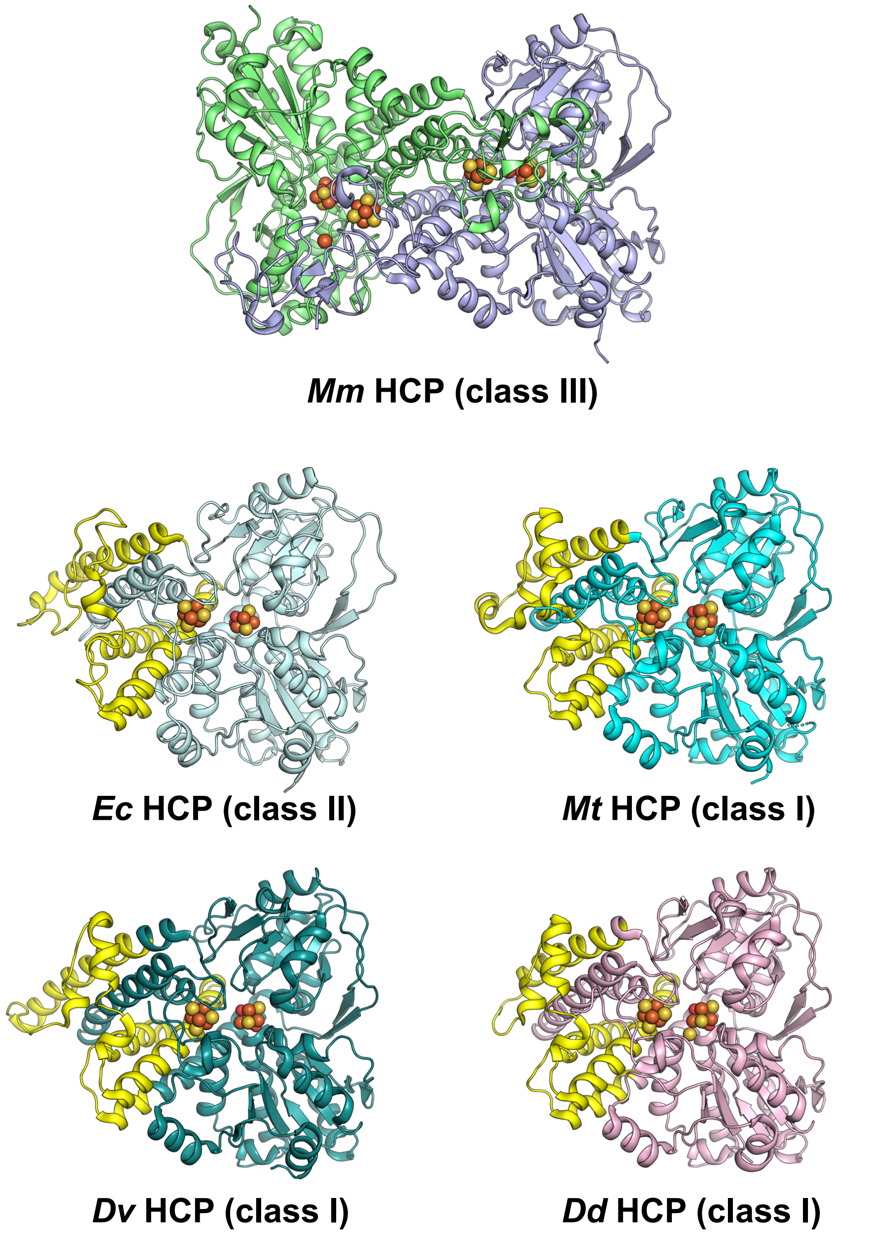

Supplementary Fig. 1. **Structural comparison of the three classes of hybrid cluster proteins (HCPs).** Overall structures of *Methanothermobacter marburgensis* HCP (*Mm* HCP) (class III), *Escherichia coli* HCP (*Ec* HCP) (class II) (PDB ID: 7DE4)[1], *Methanothermococcus thermolithotrophicus* HCP (*Mt* HCP) (class I) (PDB ID: 8CNS)[2], *Desulfovibrio vulgaris* HCP (class I) (PDB ID: 1W9M)[3], *Desulfovibrio desulfuricans* HCP (class I) (PDB ID: 1OA0)[4]. The Fe ion of the rubredoxin domain and [4Fe-4S] cluster and the hybrid cluster are represented as spheres (brown: Fe, yellow: S and red: O). The middle helical domains found only in class I and class II HCPs are coloured in yellow.

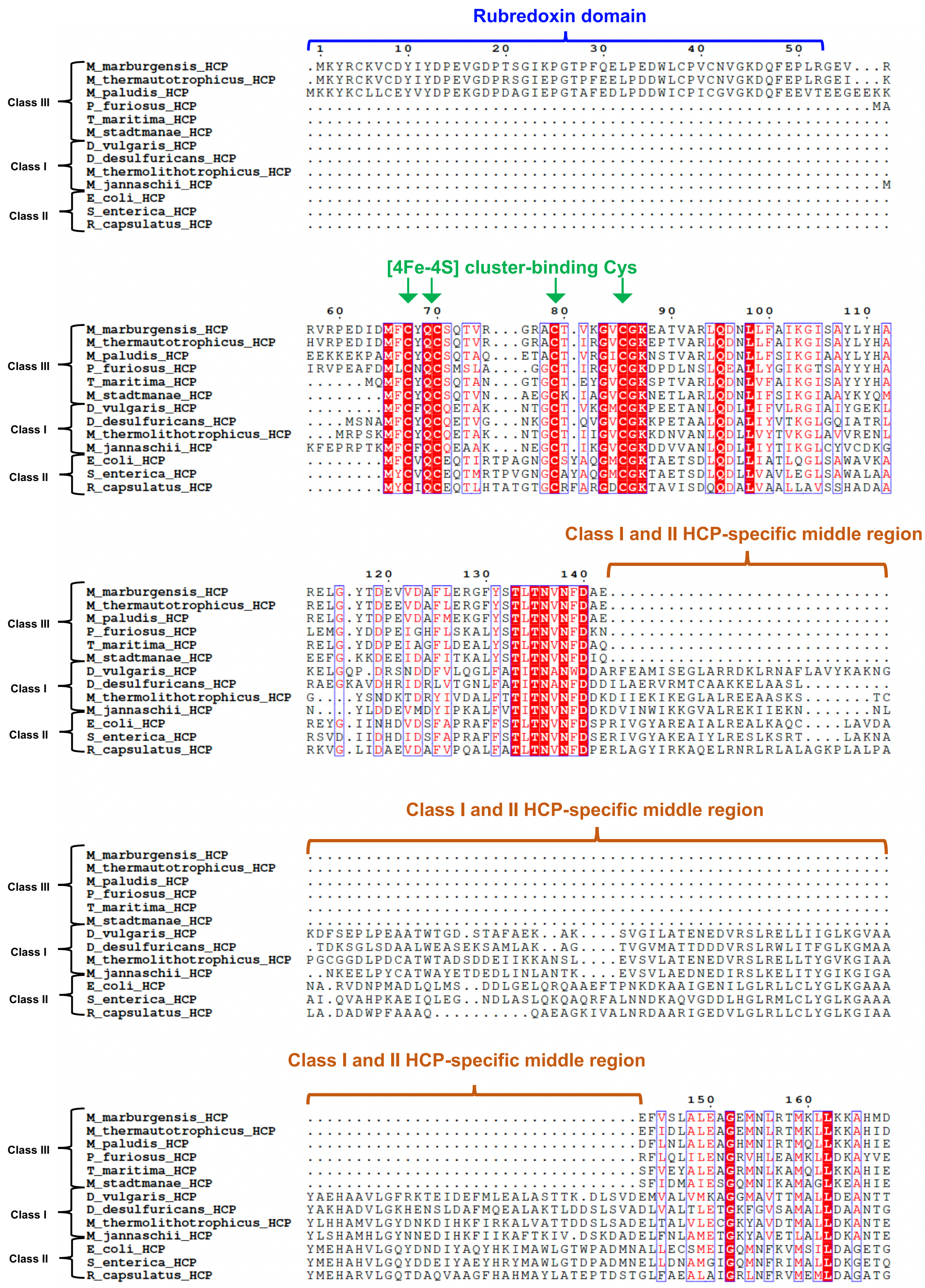
Rubredoxin domain
[4Fe-4S] cluster-binding Cys
Class I and II HCP-specific middle region
Class III
Class I
Class II
M_marburgensis_HCP
M_thermautotrophicus_HCP
M_paludis_HCP
P_furiosus_HCP
T_maritima_HCP
M_stadtmanae_HCP
D_vulgaris_HCP
D_desulfuricans_HCP
M_thermolithotrophicus_HCP
M_jannaschii_HCP
E_coli_HCP
S_enterica_HCP
R_capsulatus_HCP

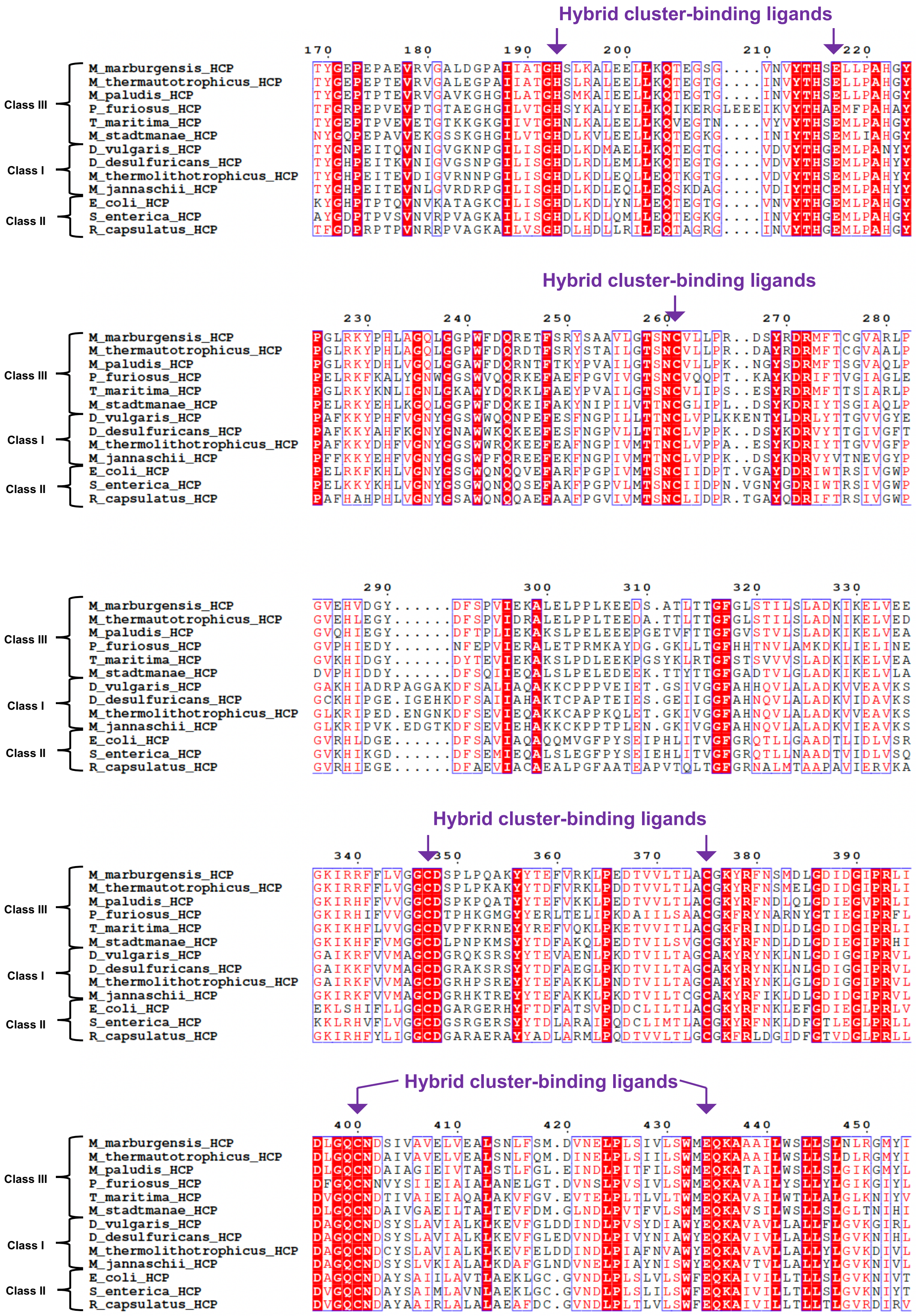

Hybrid cluster-binding ligands
Class III
Class I
Class II
M_marburgensis_HCP
M_thermautotrophicus_HCP
M_paludis_HCP
P_furiosus_HCP
T_maritima_HCP
M_stadtmanae_HCP
D_vulgaris_HCP
D_desulfuricans_HCP
M_thermolithotrophicus_HCP
M_jannaschii_HCP
E_coli_HCP
S_enterica_HCP
R_capsulatus_HCP

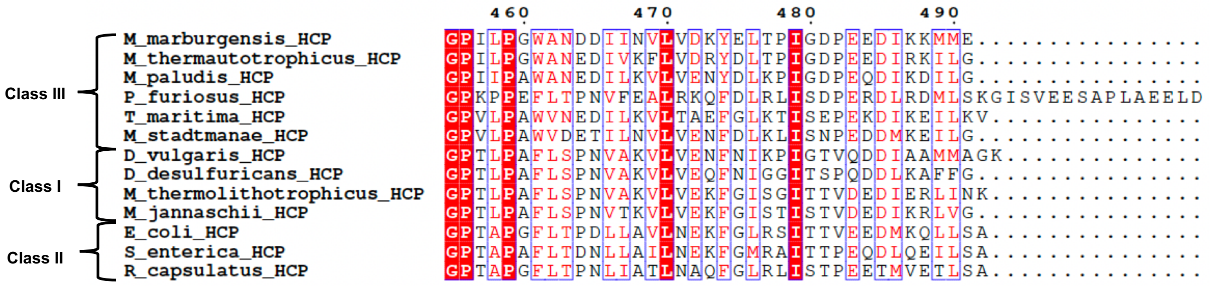

Supplementary Fig. 2. Alignments of the amino acid sequences of the HCPs in the three classes. White letters in red boxes indicate strictly conserved residues. Red letters indicate well-conserved or similar residues. M_marburgensis_HCP, *Methanothermobacter marburgensis* HCP; M_thermautotrophicus_HCP, *Methanothermobacter thermautotrophicus* HCP; M_paludis_HCP, *Methanobacterium paludis* HCP; P_furiosus_HCP, *Pyrococcus furiosus* HCP; T_maritima_HCP; *Thermotoga maritima* HCP; M_stadtmanae_HCP, *Methanosphaera stadtmanae* HCP; D_vularis_HCP, *Desulfovibrio vulgaris* HCP; D_desulfuricans_HCP, *Desulfovibrio desulfurican*s HCP; M_jannaschii_HCP, *Methanocaldococcus jannaschii* HCP; E_coli_HCP, *Escherichia coli* HCP; S_enterica_HCP, *Salmonella enterica* HCP and R_capsulatus_HCP, *Rhodobacter capsulatus* HCP. Alignments were prepared using Clustal Omega[5], and the figure was created using EsPript3[6].

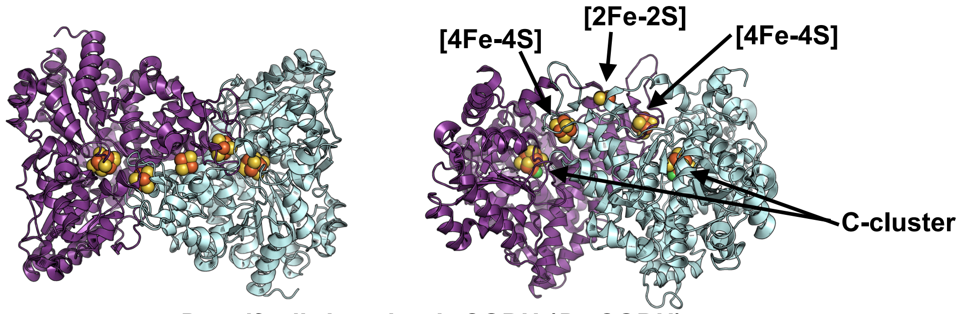

Desulfovibrio vulgaris **CODH (*Dv* CODH)**

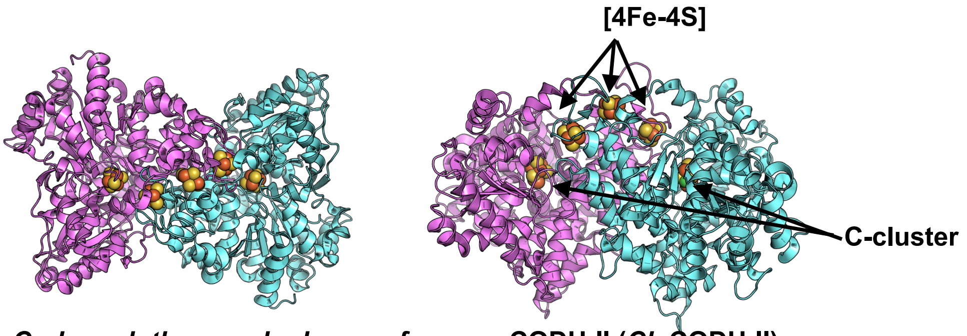

Carboxydothermus hydrogenoformans **CODH-II (*Ch* CODH-II)**

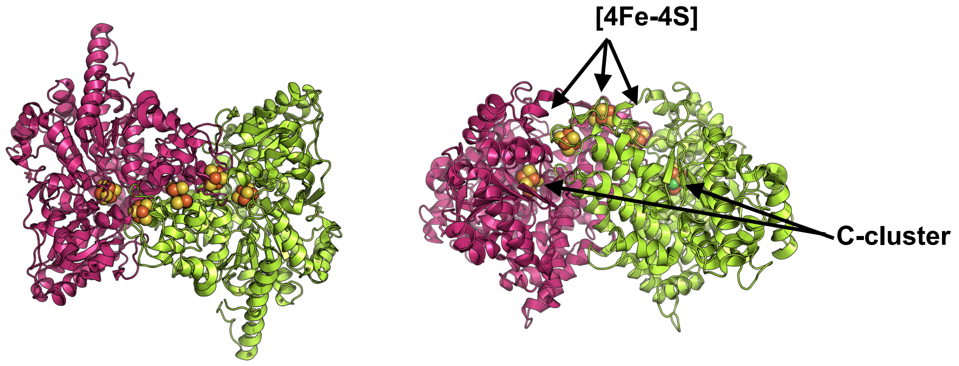

Carboxydothermus hydrogenoformans **CODH-III (*Ch* CODH-III)**

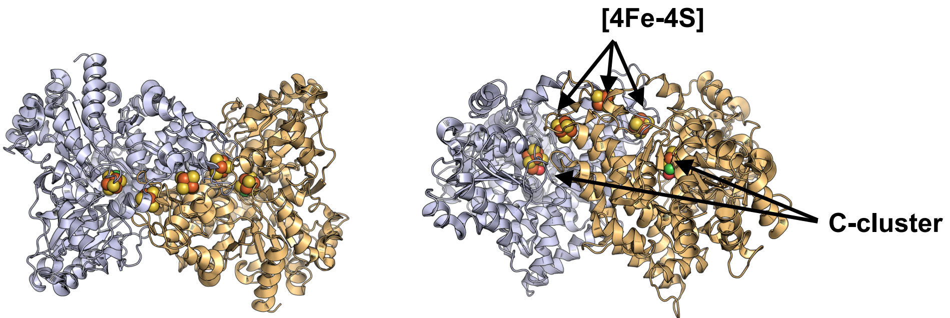

Carboxydothermus hydrogenoformans **CODH-IV (*Ch* CODH-IV)**

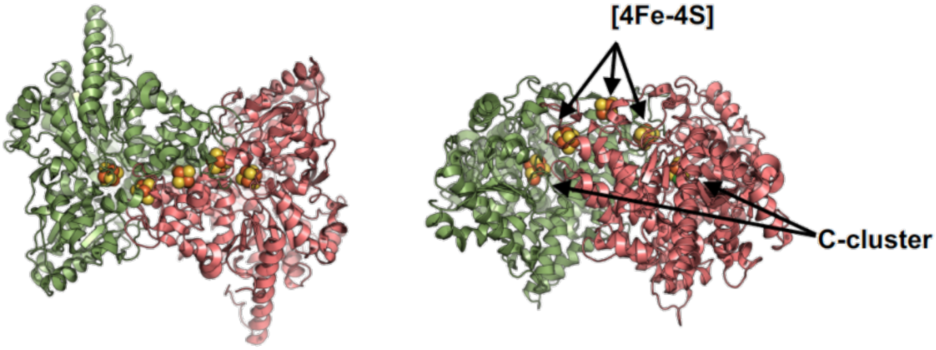

Moorella thermoacetica **CODH (*Mo* CODH)**

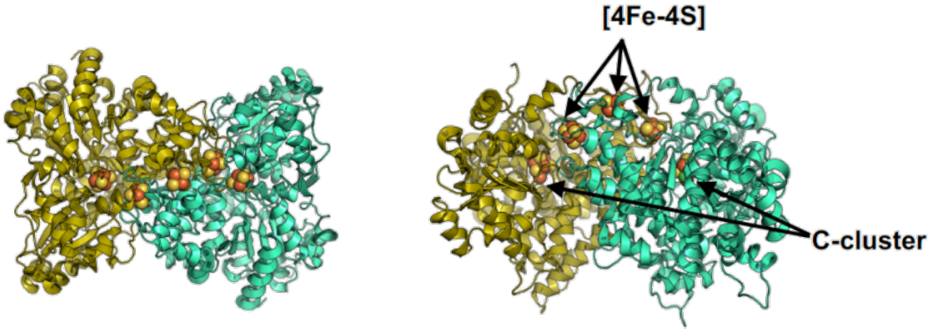

Clostridium autoethanogenum **CODH (*Ca* CODH)**

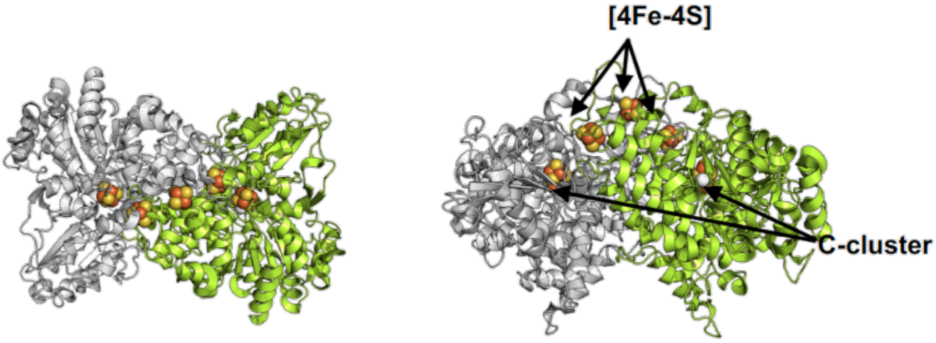

Rhodospirillum rubrum **CODH (*Rr* CODH)**

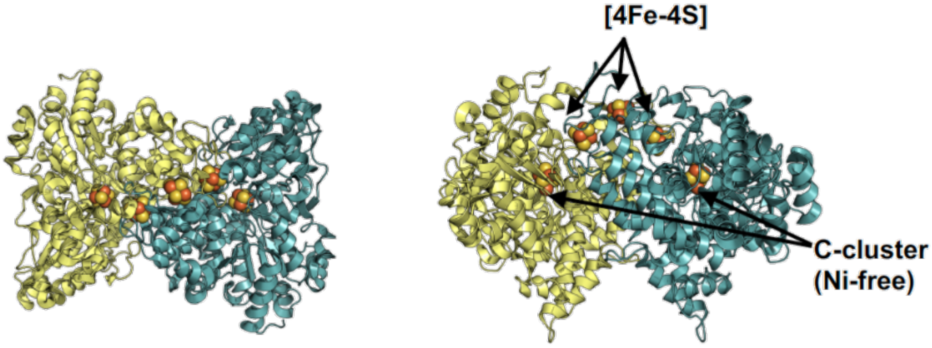

Thermococcus **sp. AM4 CODH (*Th* CODH)**

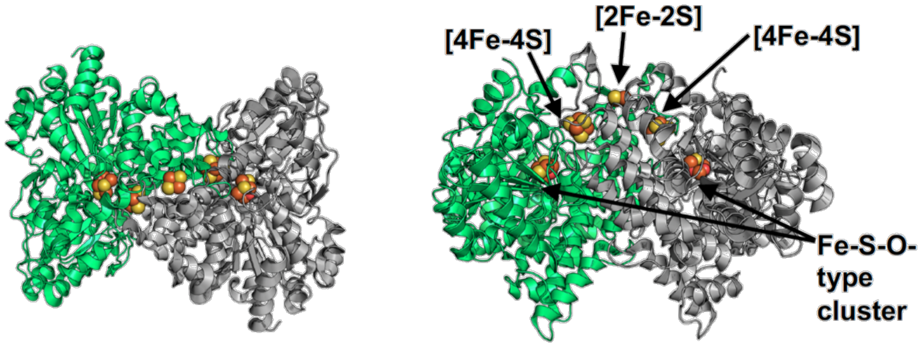

***Carboxydothermus hydrogenoformans* CODH-V (*Ch* CODH-V)**

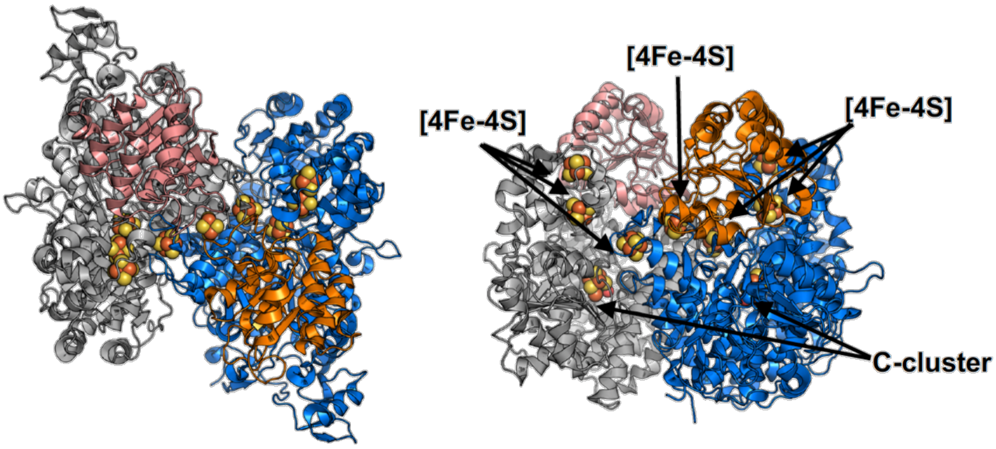

***Methanosarcina barkeri* CODH (*Mb* CODH)**

Supplementary Fig. 3. Structural comparison of CODHs and their paralogs. Top views are on the left side, whereas side views are on the right side. Metalloclusters are shown as sphere models. *Desulfovibrio vulgaris* CODH (*Dv* CODH) (PDB ID: 6B6W)[7], *Carboxydothermus hydrogenoformans* CODH-II (*Ch* CODH-II) (PDB ID: 3B51)[8], *C. hydrogenoformus* CODH-III (*Ch* CODH-III) (PDB ID: 7ZKJ)[9], *C. hydrogenoformus* CODH-IV (*Ch* CODH-IV) (PDB ID: 6ELQ)[10], *Moorella thermoacetica* CODH (*Mo* CODH) (PDB ID: 1MJG)[11], *Clostridium autoethanogenum* CODH (*Ca* CODH) (PDB ID: 6YU9)[12], *Rhodospirillum rubrum* CODH (*Rr* CODH) (PDB ID: 1JQK)[13], *Thermococcus* sp. AM4 CODH (*Th* CODH) in a Ni-free form (PDB ID: 6T7J)[14], *C. hydrogenoformus* CODH-V (*Ch* CODH-V) (PDB ID: 7B7Q)[15] and *M. barkeri* CODH (*Mb* CODH) (PDB ID: 3CF4)[16]. The structures of *Ch* CODH-III, *Mo* CODH and *Ca* CODH were extracted from those of CODH/acetyl-CoA synthase complexes[17]. *Th* CODH was obtained in the *Desulfovibrio fructosovorans*-based heterologous expression system in the absence of CooC, a C-cluster maturase from *Thermococcus* sp. AM4[14]. *Ch* CODH-V does not have a C-cluster, and thus, does not exhibit CO reductase activity, although it is structurally homologous to active CODHs[15]. *Mb* CODH is an archaeal CODH and comprises α and ε subunits, unlike other CODHs[16].

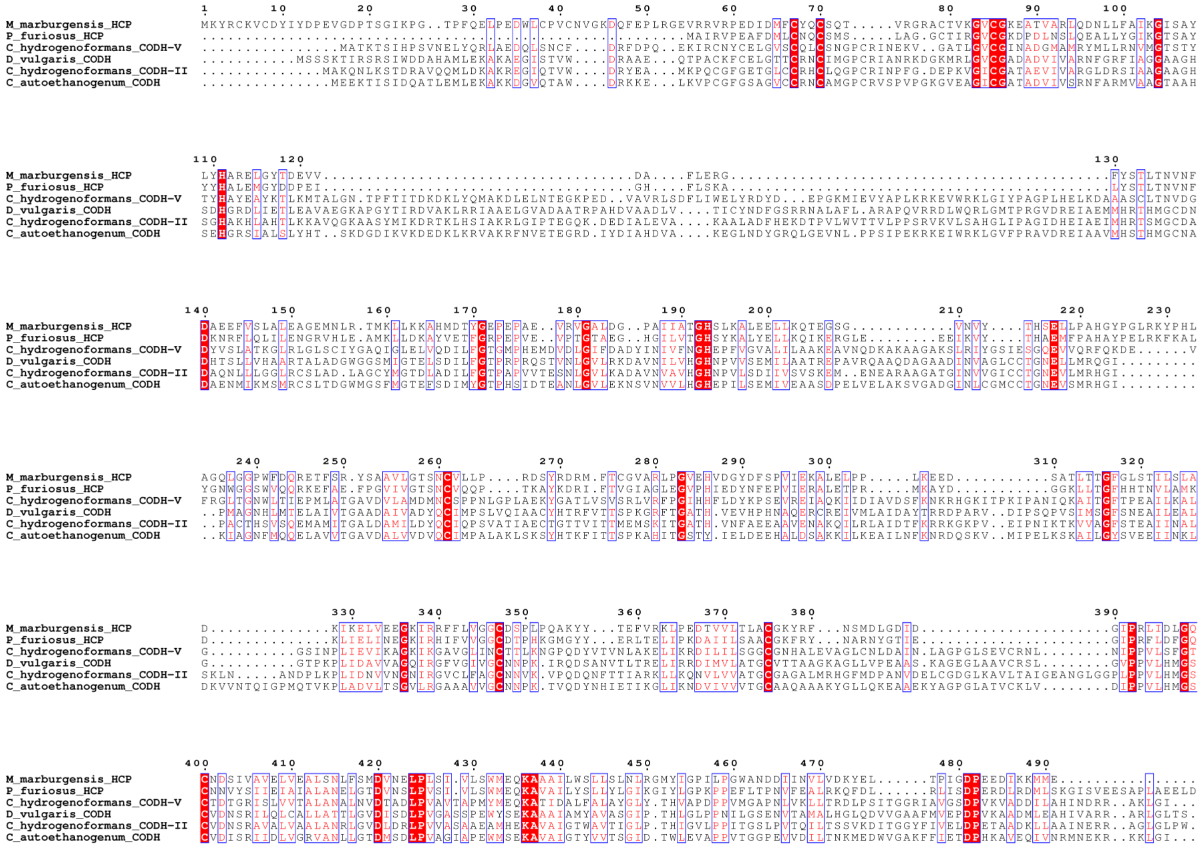

Supplementary Fig. 4. Amino acid sequence alignments of class III HCPs and CODHs. M_marburgensis_HCP (*Mm* HCP), *Methanothermobacter marburgensis* HCP; P_furiosus_HCP, *Pyrococcus furiosus* HCP; C_hydrogenoformas_CODH-V, *Carboxydothermus hydrogenoformus* CODH-V; D_vulgaris_CODH, *Desulfovibrio vulgaris* CODH; C_hydrogenoformas_CODH-II, *Carboxydothermus hydrogenoformus* CODH-II and C_autoethanogenum_CODH, *Clostridium autoetanogenum* CODH. The alignments was made using Clustal Omega[5]. The figure of the alignments was created using ESpript3[6]. White letters in the red box background indicate strictly conserved residues. The red bold letters in the blue box indicate well-conserved or similar residues. The residue number of *Mm* HCP are shown on the alignments.

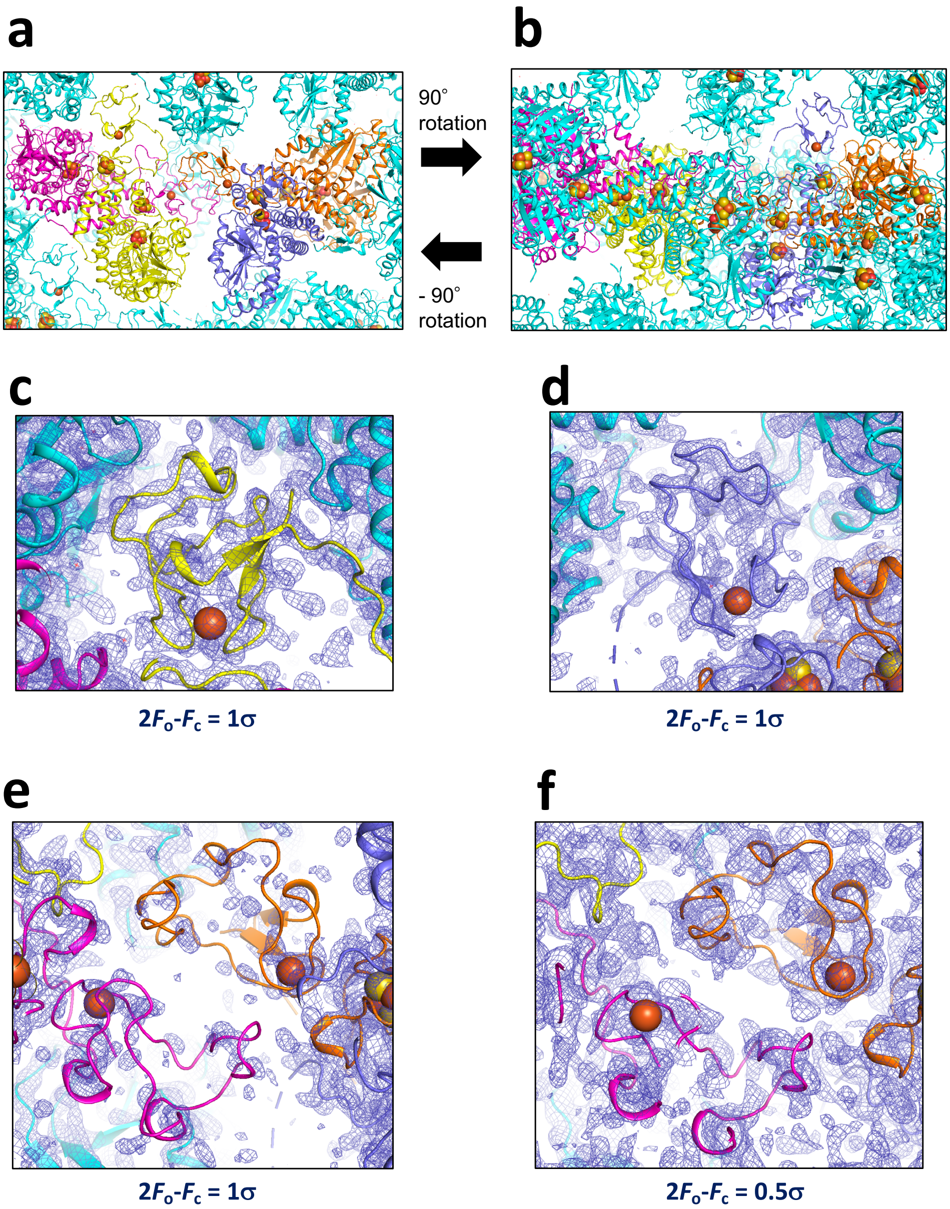

Supplementary Fig. 5. Molecular packing of *Mm* HCP WT crystal. a, b Packing of two homodimers (four monomers) of *Mm* HCP WT. Four molecules in one asymmetric unit, and one homodimer is coloured in magenta and yellow, and the other in slate-blue and orange. Cyan-coloured molecules are surrounding two homodimers of *Mm* HCP WT the in one asymmetric unit. Metallocentres are shown as spheres (Fe: brown, S: yellow, O: red). Linker regions of one homodimer in slate-blue and orange exhibit weak or no electron density, implying that these regions are flexible. **c–f** The $2F_o$-F_c electron density map showing the rubredoxin (Rd) domains of *Mm* HCP WT and the surrounding regions. The map (blue mesh) was contoured at 1σ or 0.5σ. Two rubredoxin domains in yellow and late-blue were clearly visible. This may be because these domains are well-packed among the surrounding *Mm* HCP molecules, which resulting in reducing possible fluctuating positions of the domains in the crystal. *Mm* HCP molecules. In contrast, the rubredoxin domains in magenta and orange show weaker density, because these molecules appear to be less well-packing.

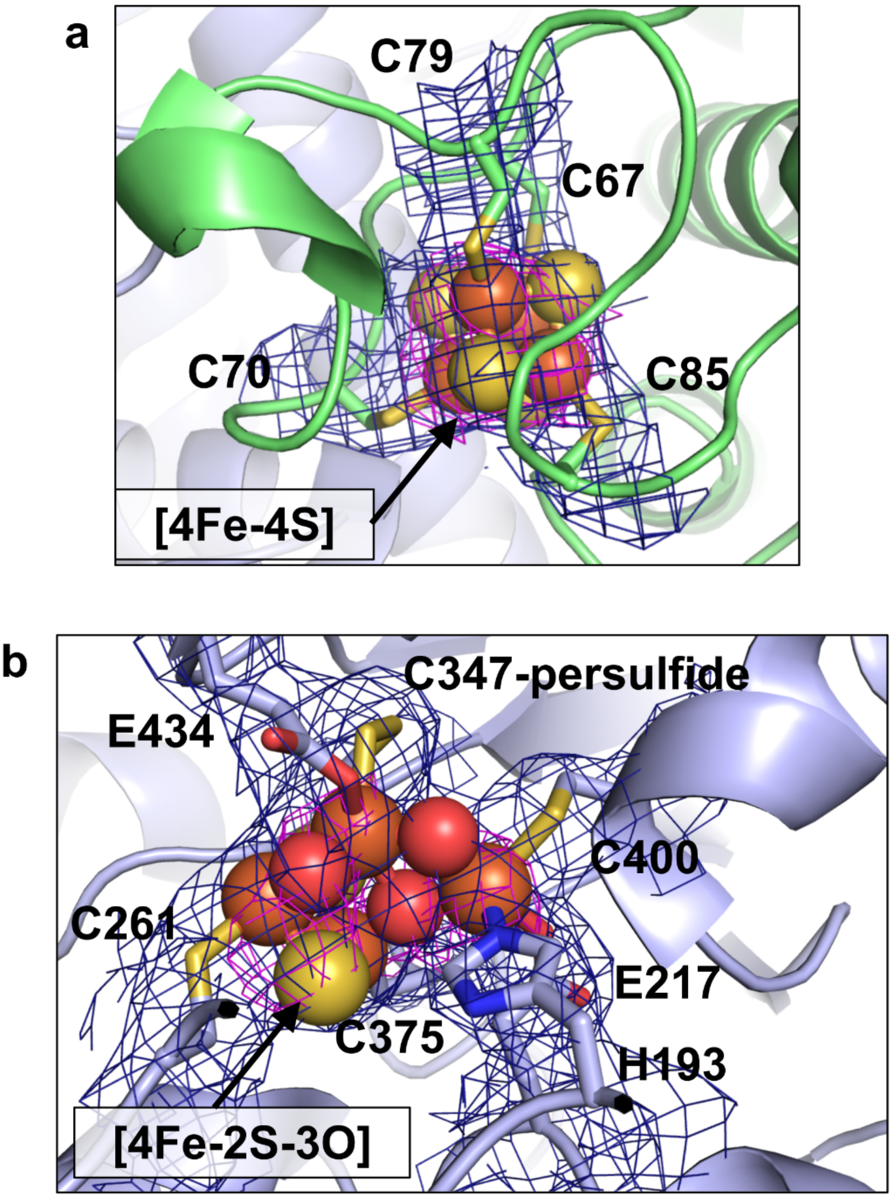

Supplementary Fig. 6. Structures of the [4Fe-4S] and hybrid cluster-binding sites of *M. marburgensis* HCP. **a** [4Fe-4S] cluster and **b** hybrid cluster, which was modelled as a [4Fe-2S-3O] cluster, in an as-isolated form. The $2F_o$-F_c electron density map (blue mesh) was contoured at 1σ. The Fe-anomalous density map (magenta mesh) was contoured at 3σ. Fe, S O atoms are shown as brown, yellow, and red spheres, respectively. The amino acid ligands of the clusters are represented using stick models.

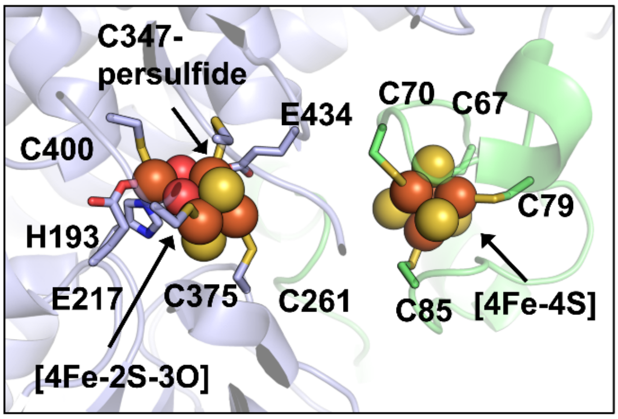

***Mm* HCP (class III)**

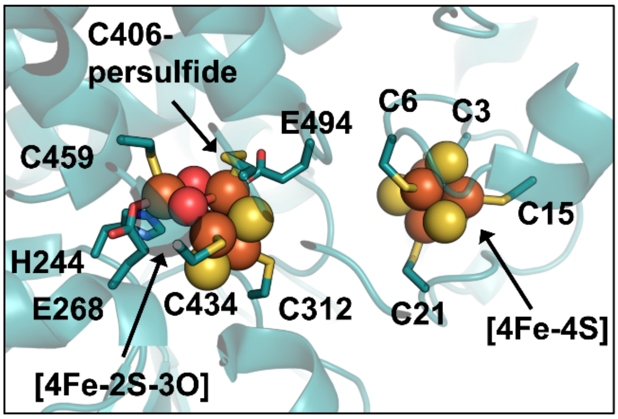

***Dv* HCP (class I)**

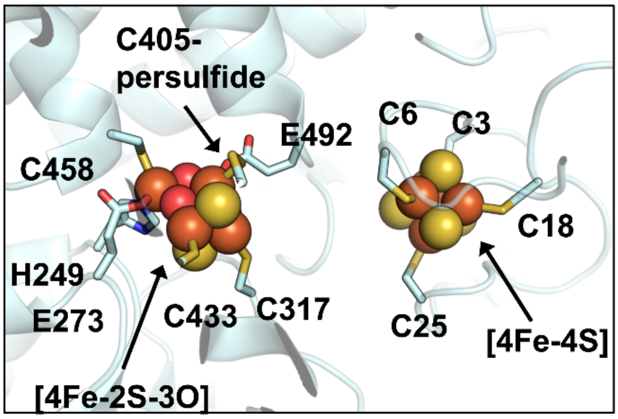

***Ec* HCP (class II)**

Supplementary Fig. 7. Comparison of the [4Fe-4S] cluster- and hybrid cluster-binding sites of the three distinct HCP classes. The binding site of the hybrid cluster, which was modelled as a [4Fe-2S-3O] cluster, in an as-isolated form. Fe, S and O atoms of the metalloclusters are shown as brown, yellow and red spheres, respectively. The amino acid ligands of the clusters are represented using stick models.

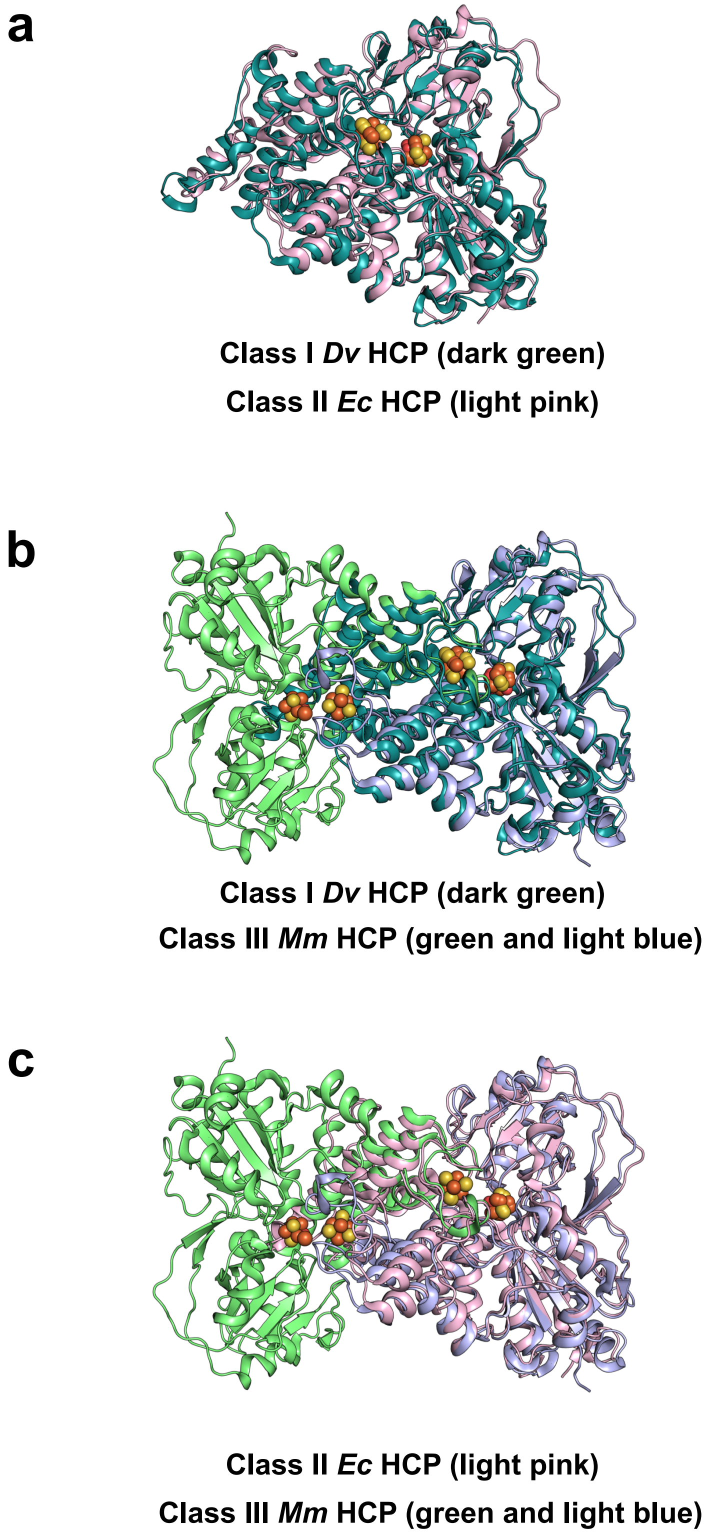

Supplementary Fig. 8. **Superimpositions of different classes of HCPs.** **a** Class I *Dv* HCP and class II *Ec* HCP. **b** Class I *Dv* HCP and class III *Mm* HCP. **c** Class II *Ec* HCP and class III *Mm* HCP. [4Fe-4S] and [4Fe-2S-3O] clusters are shown as sphere models. Rubredoxin domains were omitted for clarity of the figures.

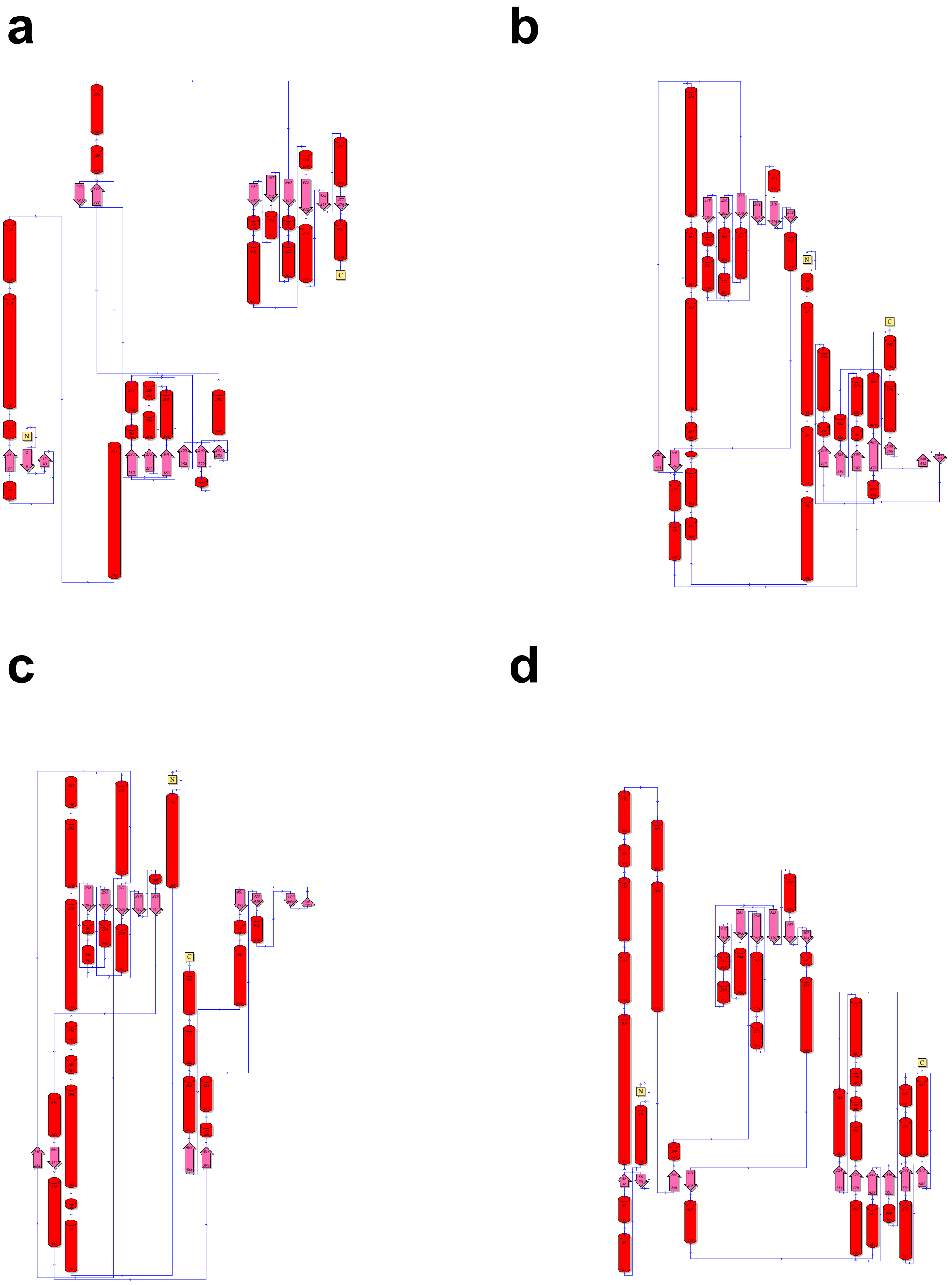

Supplementary Fig. 9. Topology diagrams for the HCPs and CODHs. Figures were created using PDBSum[18]. **a** Class III *Mm* HCP WT (PDB ID: 7E0L), **b** class I *Dd* HCP (PDB ID: 1OA0), **c** class II *Ec* HCP (PDB ID: 7DE4), and **d** *Ch* CODH-II (PDB ID: 3B51). The red cylinders and magenta arrows indicate α-helices and β-strands, respectively.

a

```
MmHCP_E88-L115        EATVARLQDNLLFAIKGISAYLYHAREL
DvHCP_E25-L51         -EETANLQDLLIFVLRGIAIYGEKLKEL
DvHCP_E142-L168       -EDVRSLRELLIIGLKGVAAYAEHAAVL
                         .  *:: *:: ::*:: *  :   *
```

b

```
MmHCP_G116-E143       GYTDEVVDAFLERGFYSTLTNVNFDAEE
DvHCP_G52-R79         GQPDRSNDDFVLQGLFATITNANWDDAR
DvHCP_G169-D189       GFRKTEIDEFMLEALASTTKD-------
                      *  .   * *: ..: :* .:
```

c

```
MmHCP_F144-Y170       -----FVSLALEAGEMNLRTMKLLKKAHMDTY-------
DvHCP_F80-D109        --FEAMIS-----EGLA--RRDKLRNAFLAVYKAKNGKD
DvHCP_L190-Y221       LSVDEMVALVMKAGGMAVTTMALLDEANTTTY-------
                           :::       :       * :*   .*
```

Supplementary Fig. 10. Amino acid sequence alignments of the regions of the inner helices of class III *Mm* HCP and inner and outer helices of class I *Dv* HCP. a Blue-coloured inner helix (E88–L115) of *Mm* HCP in Fig. 2 is aligned to outer (E25–L51) and inner (E142–L168) helices of *Dv* HCP. **b** Yellow-coloured inner helix (G116–R143) of *Mm* HCP in Fig. 2 is aligned to outer (G52–R79) and inner (G169–D189) helices of *Dv* HCP. **c** Orange-coloured inner helix (F144–Y170) of *Mm* HCP in Fig. 2 is aligned to outer (F80–D109) and inner (L190–Y221) helices of *Dv* HCP. Asterisks and colons in the bottoms of the alignments indicate conserved residues and residues having similar properties, respectively. The alignments were performed using Clustal Omega[5].

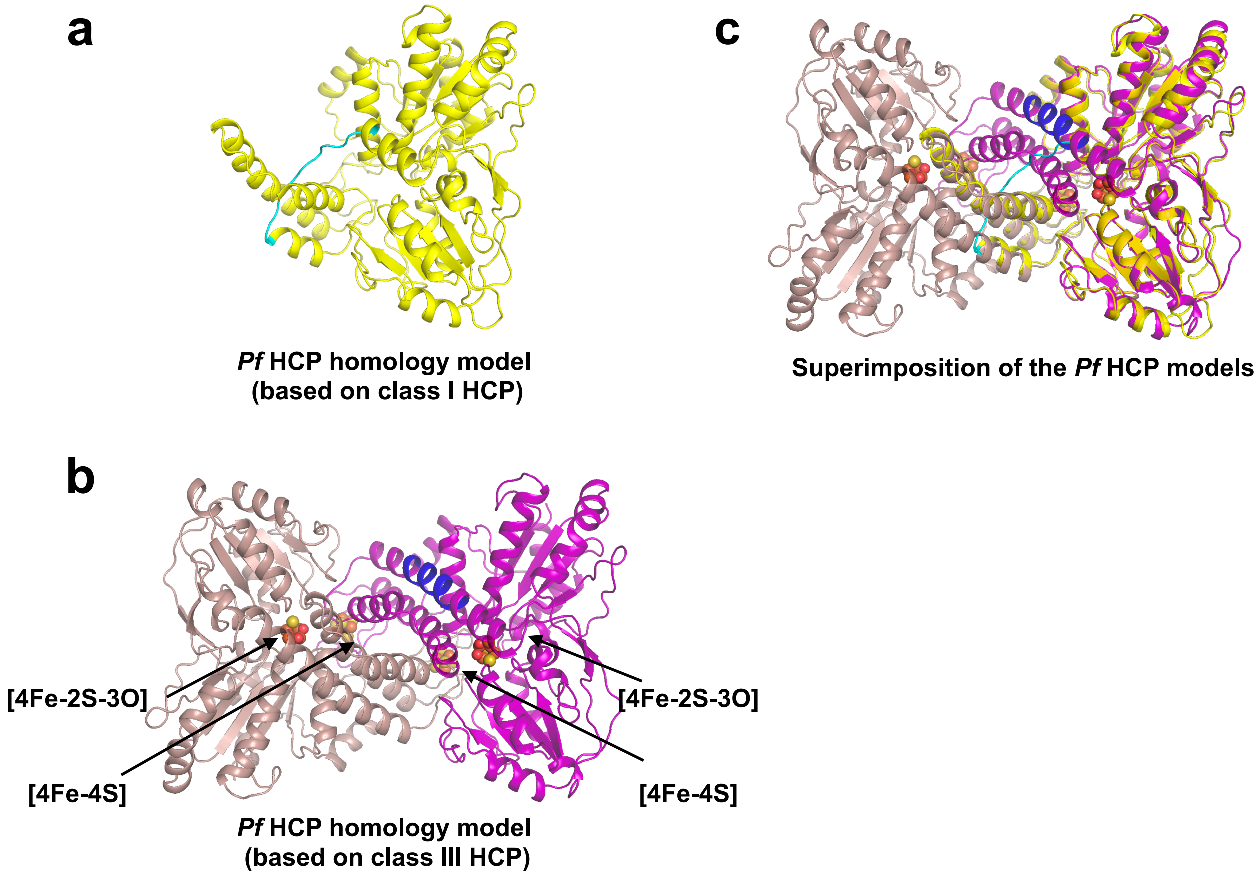

Supplementary Fig. 11. Homology models of class III *Pyrococcus furiosus* HCP (*Pf* HCP). a The model based on class I *D. vulgaris* HCP (*Dv* HCP) (PDB ID: 1W9M), a monomeric HCP. **b** The model based on class III *Mm* HCP, a homodimeric HCP. The homology models were created using SWISS-MODEL[19]. **c** Superimposition of the two homology models of *Pf* HCP. There could be no major differences in the homology modelling on the HCP domains. However, in the *Dv* HCP-based model, there was a long loop (Leu96–Met106, coloured in cyan) connecting two helices at the middle region. In contrast, in the *Mm* HCP-based model, the corresponding region was modelled as a helix (Leu96–Met106, coloured in blue), and not as a long loop. The long loop of the *Dv* HCP-based model may cause steric crashes if the two models were attempted to be placed as a homodimer, although *Pf* HCP was a homodimer. Thus, better homology modelling was achieved by using homodimeric *Mm* HCP as the template model. [4Fe-4S] and [4Fe-2S-3O] clusters are shown as spheres. Metalloclusters of the monomeric *Pf* HCP model were omitted for clarity of the focused loop in cyan. Metallocentres are shown as spheres. The Fe, S O atoms are coloured in brown, yellow and red, respectively.

.

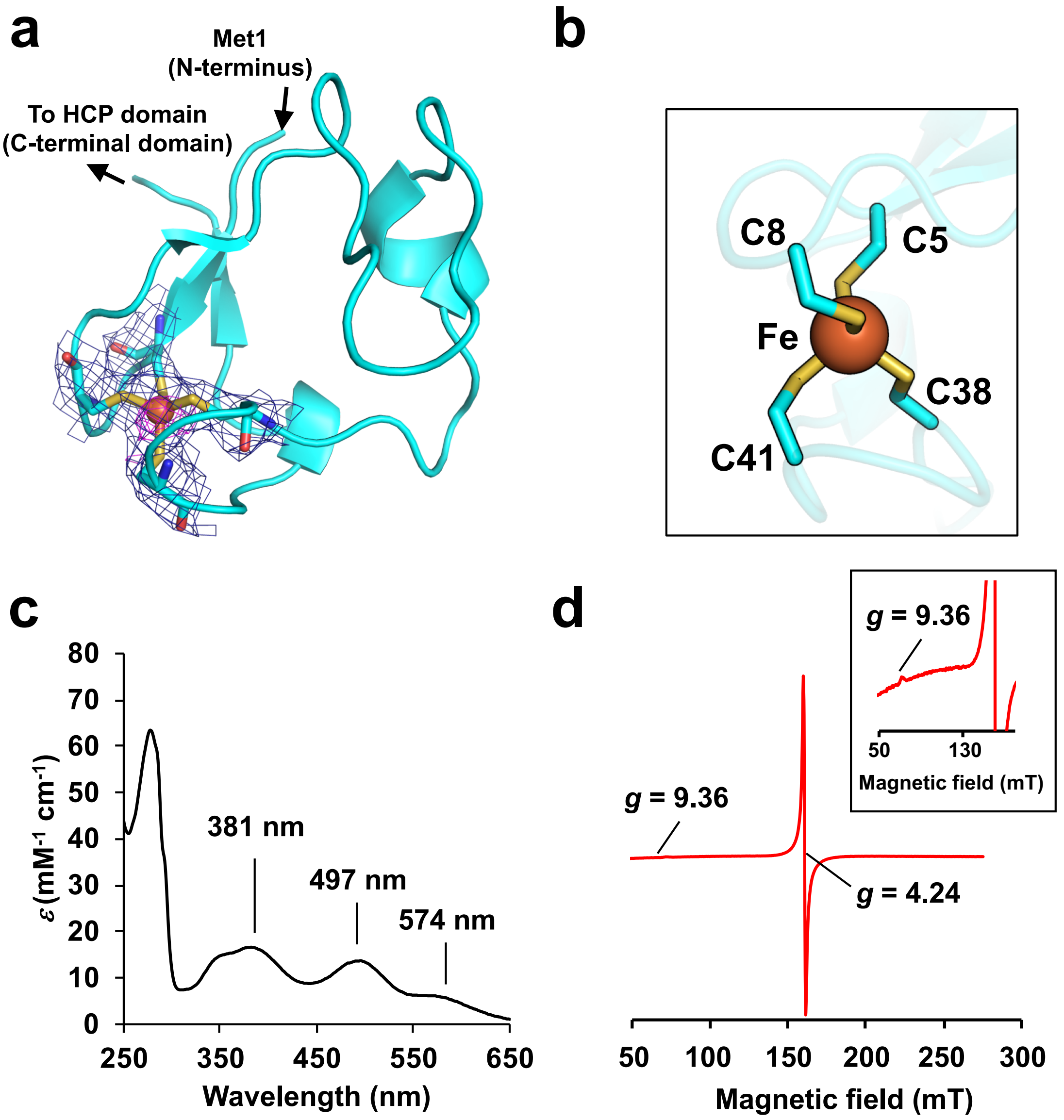

Supplementary Fig. 12. Rubredoxin domain of *Mm* HCP. a Structure of the rubredoxin domain of *Mm* HCP. The $2F_o$-F_c electron density map (blue mesh) was contoured at 1σ. The Fe-anomalous density map (magenta mesh) was contoured at 3σ. The Fe atom is represented as a brown sphere. Cys residues that act as Fe ligands are represented as stick models. **b** Fe centre of the rubredoxin domain of *Mm* HCP. Fe ion and ligands are represented as brown spheres and stick models, respectively. **c** UV–visible spectrum of the rubredoxin domain in the as-isolated state. Protein concentration was 14 μM. The UV–visible spectrum was recorded using a quartz cuvette with 1cm light path. **d** EPR spectrum of the rubredoxin domain in the as-isolated state. The inset provides an expanded view of the spectrum in a low magnetic field. Protein concentration was 0.28 mM.

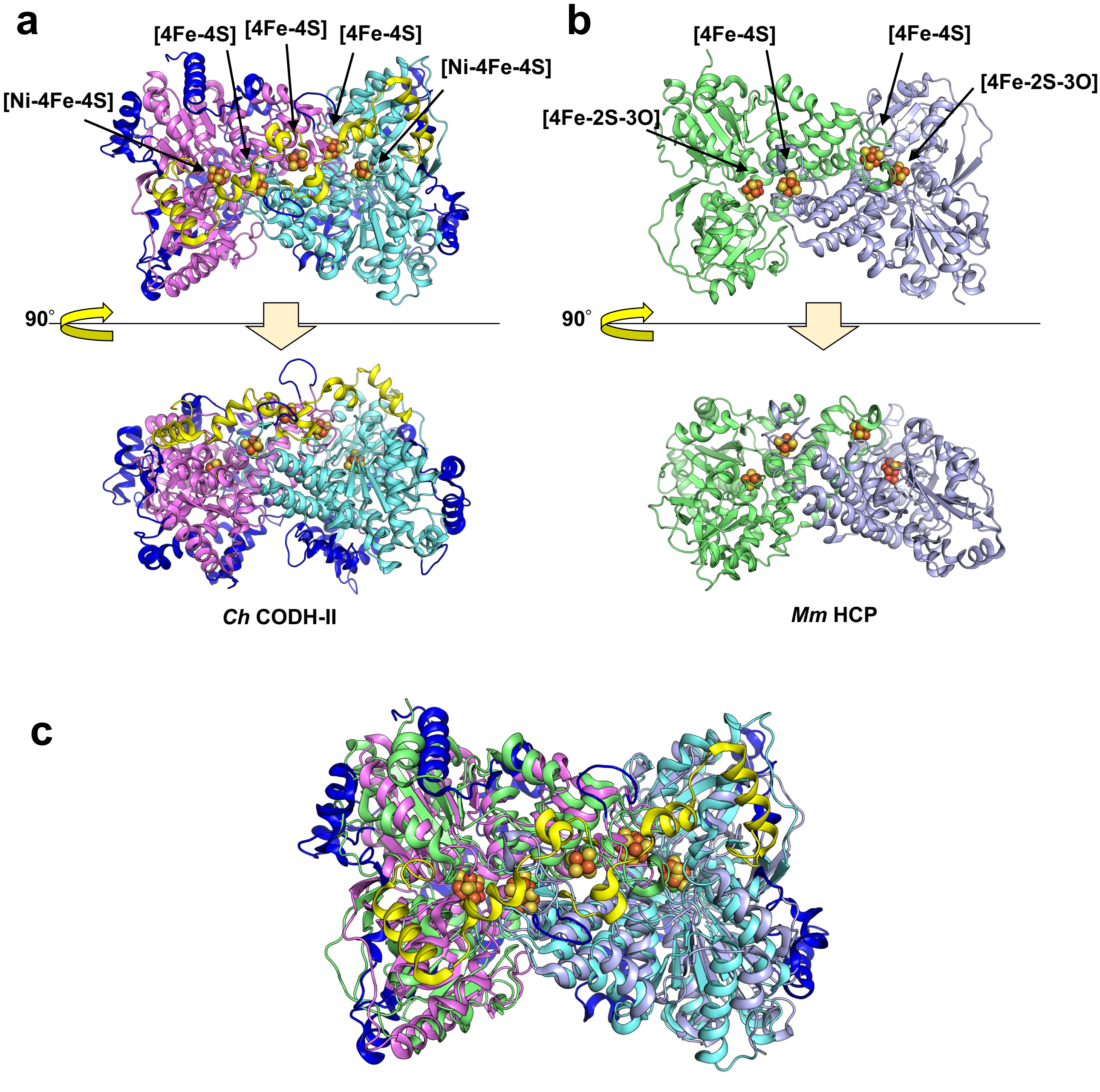

Supplementary Fig. 13. Comparison of the protein folding of *Ch* CODH-II and *Mm* HCP. a *Ch* CODH-II, **b** *Mm* HCP. **c** Superimposition of *Ch* CODH-II and *Mm* HCP. Blue-coloured regions consisting the surface area of *Ch* CODH-II indicate their non-superposed regions to *Mm* HCP. Yellow-coloured regions of *Ch* CODH-II are CODH-specific sequences with the Cys-motif for binding an interfacial Fe-S cluster at the N-terminus of CODHs. Metalloclusters are indicated as spheres.

a

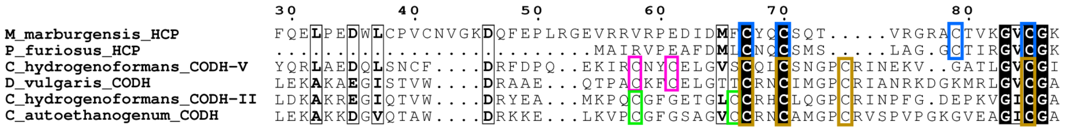

b

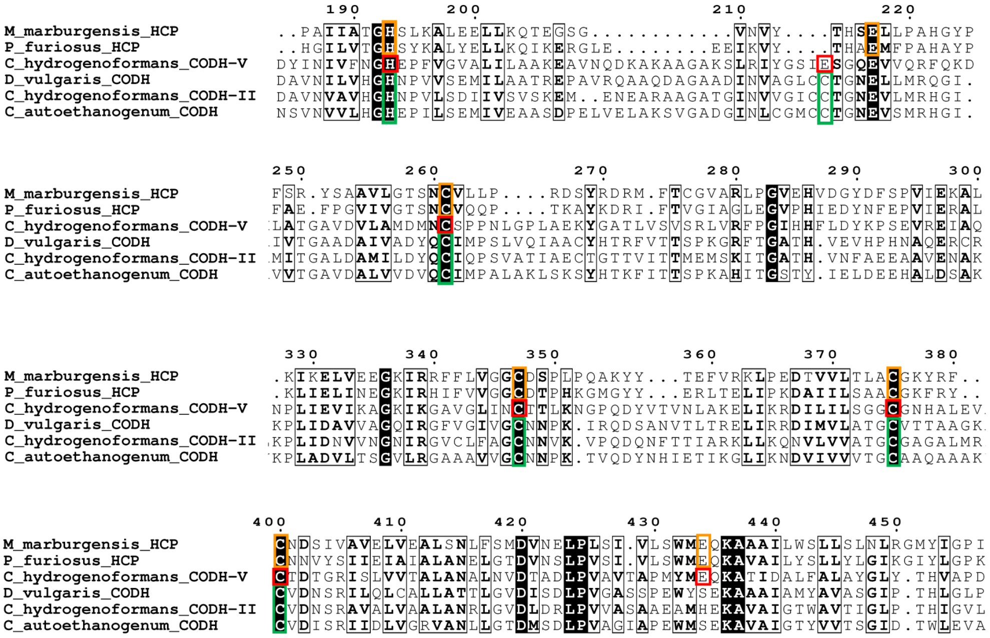

Supplementary Fig. 14. Amino acid sequence alignments of the metallocluster-binding regions of class III HCPs and CODHs. Alignments were made with Clustal Omega[5]. The figure of the alignments was created using ESpript3[6]. White letters in the black box background indicate strictly conserved residues. The bold letters in the black box indicate well-conserved or similar residues. **a** N-terminal Cys-rich regions. Blue boxes indicate the Cys residues binding to the [4Fe-4S] cluster in HCPs. Magenta and light green boxes indicate the Cys residues binding to the interfacial [2Fe-2S] cluster in some CODHs and the interfacial [4Fe-4S] cluster in other CODHs, respectively. Light brown boxes indicate the Cys residues binding to the [4Fe-4S] cluster conserved in all CODHs. **b** Hybrid cluster- or C-cluster-binding regions. Orange boxes indicate residues binding to the hybrid cluster. Red boxes indicate residues binding to the Fe-S-O cluster in *Ch* CODH-V. Green boxes indicate residues binding to the C-cluster in CODHs. The number and types of ligands are similar for HCPs and *Ch* CODH-V, but Glu295 of *Ch* CODH-V is not aligned to the corresponding Glu residue of the HCPs (e.g. Glu 217 of *Mm* HCP). Instead, Glu295 of *Ch* CODH-V is aligned to one of the conserved Cys residues of the CODHs (e.g. Cys with the C-cluster. Furthermore, Glu556 of *Ch* CODH-V and the other conserved Glu residues of HCPs (e.g. Glu 434 of *Mm* HCP) are aligned.

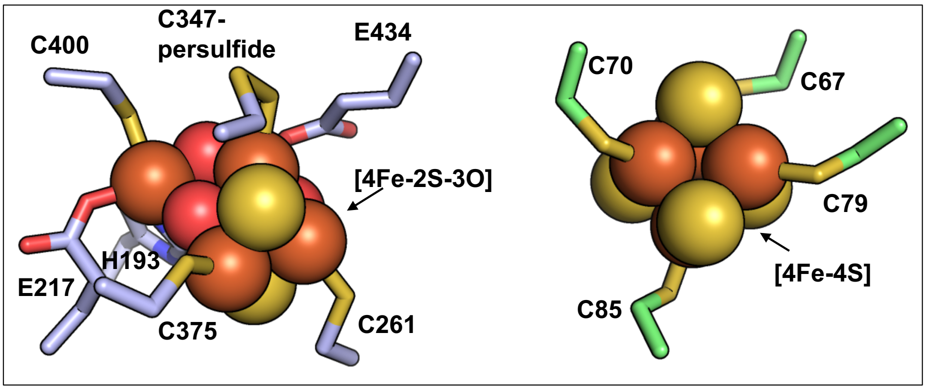

***Mm* HCP (class III)**

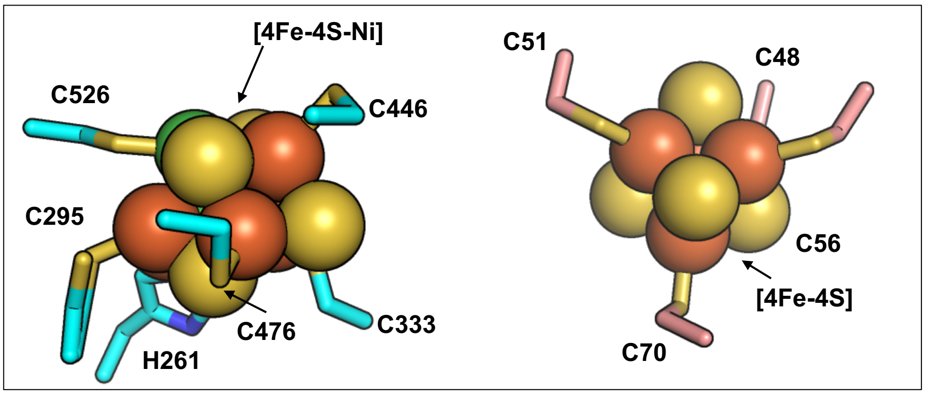

***Ch* CODH-II**

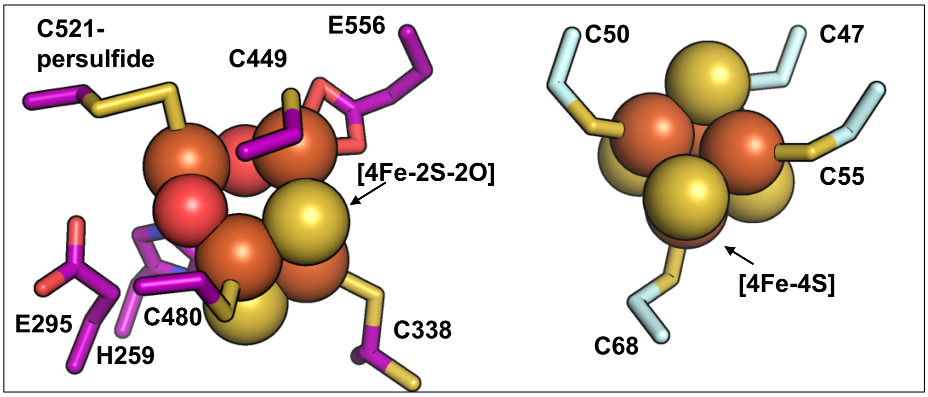

***Ch* CODH-V**

Supplementary Fig. 15. Comparison of the metallocluster-binding sites of *Mm* HCP, *Ch* CODH-II, and *Ch* CODH-V. Fe, S, and O atoms are shown as brown, yellow, and red spheres, respectively. Cys-persulfide ligands of *Mm* HCP and *Ch* CODH-V (PDB ID: 7B7Q) were positioned differently, whereas other ligand types were similar in both. The position of Glu295 is specific for *Ch* CODH-V, and this position is Cys in the other CODHs (e.g. *Ch* CODH-II, PDB ID: 3B51) in Supplementary Fig. 14. The residues of Glu295 of *Ch* CODH-V and Glu217 of *Mm* HCP are at equivalent positions, but these residues are not aligned in the amion acid sequence alignments. Of note, Glu295 and His259 of *Ch* CODH-V are not ligated to metals of its Fe-S-O-type cluster ([4Fe-2S-2O] + $2H_2O$), whereas Glu217 and His193 of *Mm* HCP are ligated to one Fe ion of the [4Fe-2S-3O] cluster.

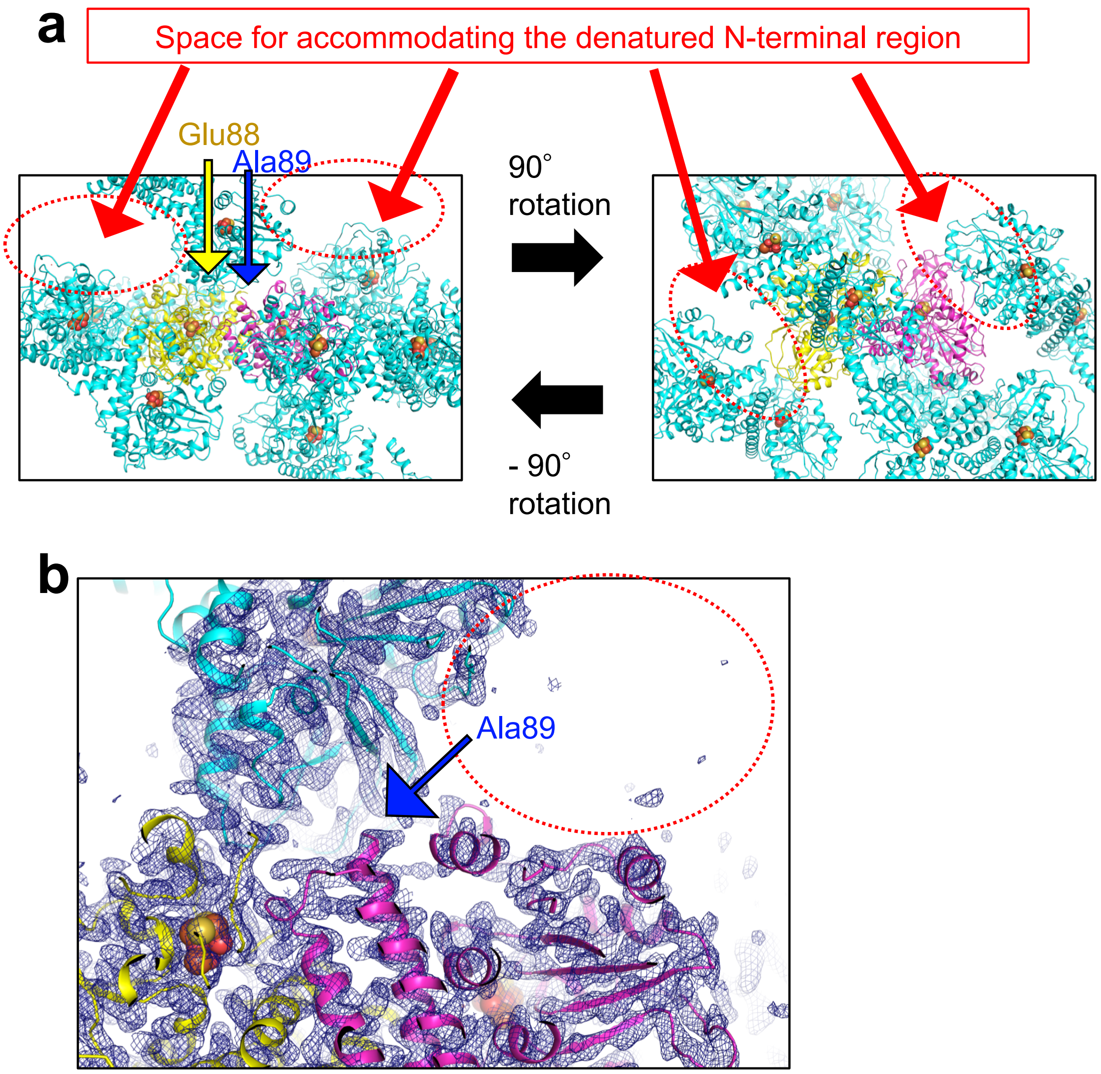

Supplementary Fig. 16. Molecular packing of *Mm* HCP C67Y in crystal. a. Packing of *Mm* HCP C67Y variant. Two molecules (yellow and magenta) as one homodimer are in one asymmetric unit. Cyan-coloured molecules are packed molecules surrounding yellow- and magenta-coloured ones. There is space (indicated by red-dashed ellipses) for accommodating the disordered N-terminal regions, although no electron density for this region is visible. **b.** Close up view of the N-terminal region of the *Mm* HCP C67Y variant with the $2F_o$-F_c electron density map contoured at 1σ. One monomer is visible from Ala89 in the yellow-coloured molecule, and the other from Glu88 in the magenta coloured-molecule. The N-terminal regions of two molecules are not modelled because of no electron density, indicating this region is disordered.

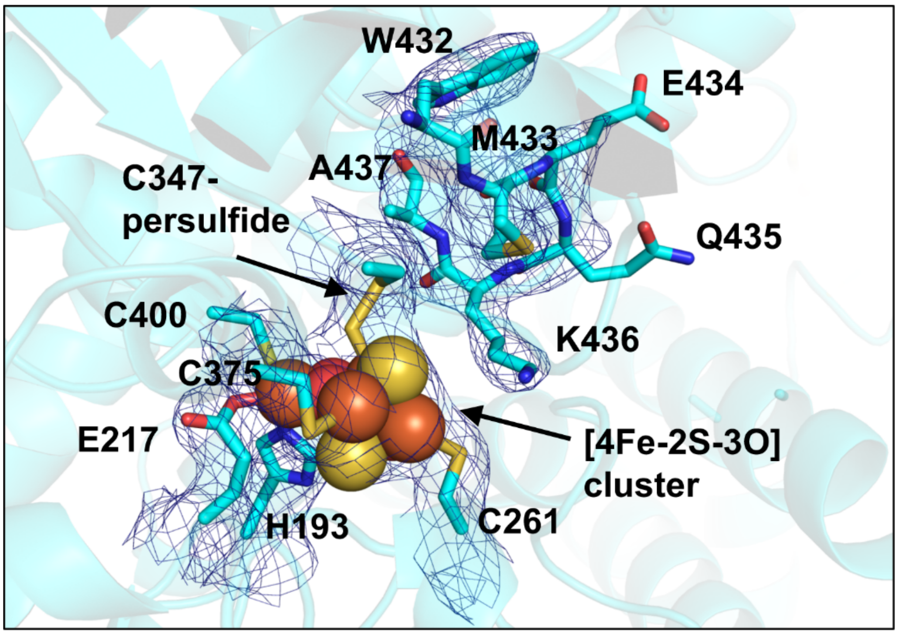

Supplementary Fig. 17. Structure of the hybrid cluster-binding site of the *Mm* HCP C67Y variant. The hybrid cluster was modelled as a [4Fe-2S-3O] cluster of *Mm* HCP WT, although the resolution was not high enough to assess the details of the cluster structure. The $2F_o$-F_c electron density map (blue mesh) was contoured at 1σ. Fe, S O atoms are shown as brown, yellow and red spheres respectively. Amino acid ligands of the hybrid cluster and some residues, which were shifted toward the solvent area owing to the C67Y mutation, are represented using stick models.

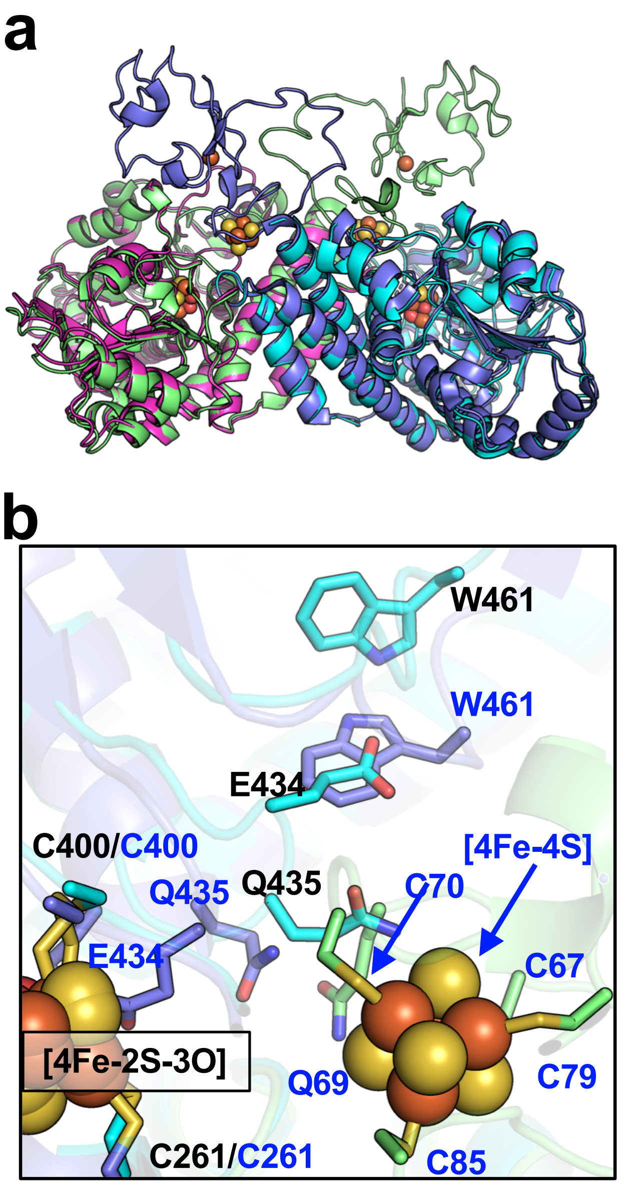

Supplementary Fig. 18. Superimposition of *Mm* HCP WT and the C67Y variant. a Overall structures of *Mm* HCP WT (light green and blue) and the C67Y variant (magenta and cyan). Metallocentres are shown as spheres (Fe: brown, S: yellow, O: red). **b** Hybrid cluster-binding sites of *Mm* HCP WT and the C67Y variant. Residues of the *Mm* HCP WT and the C67Y variant are blue- and black-labelled, respectively. Glu434 of the C67Y variant is not ligated to the hybrid cluster, but located at the equivalent position to the Trp461 of WT.

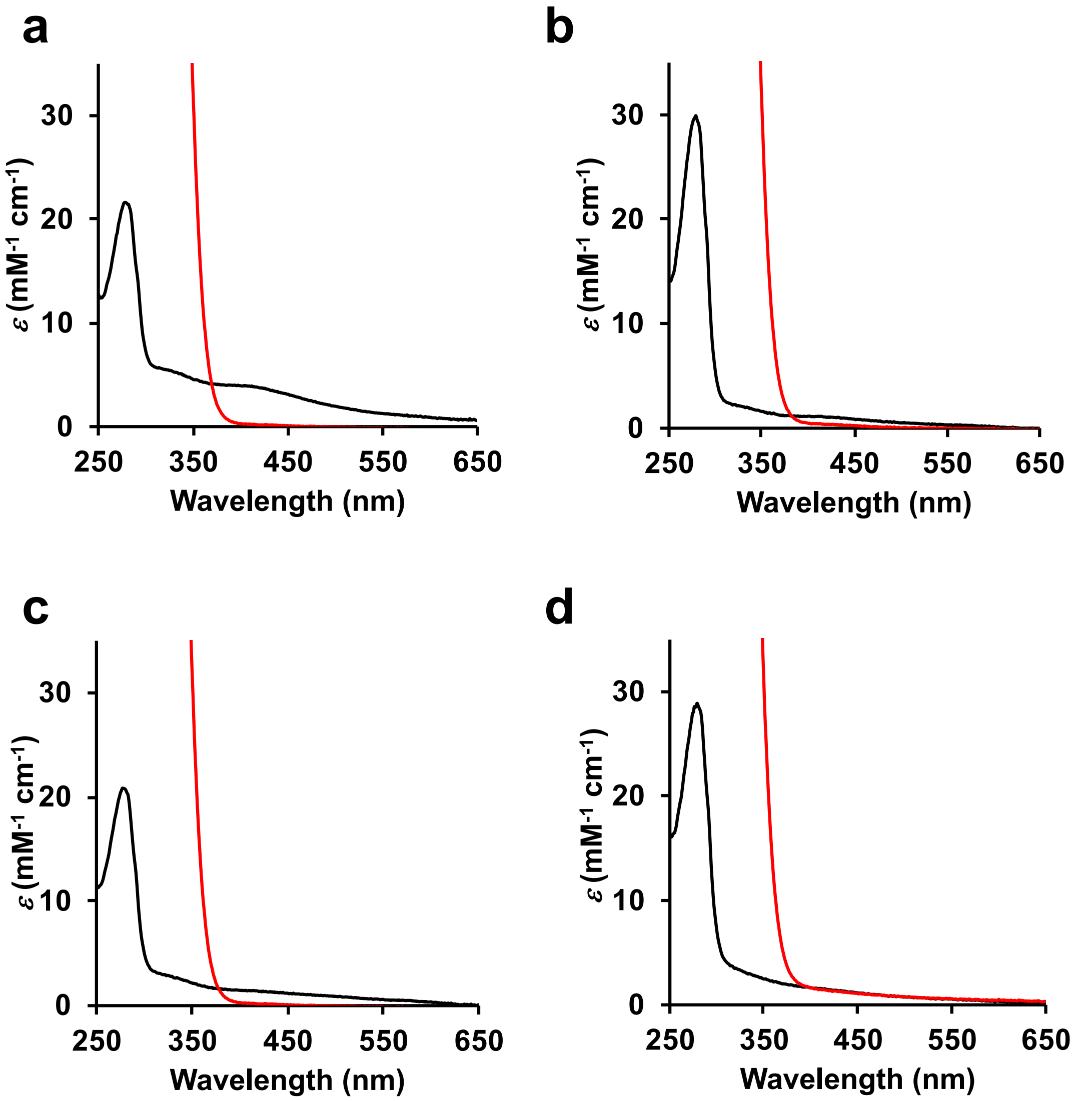

Supplementary Fig. 19. UV–visible spectra. a *Mm* HCP WT. **b** *Mm* HCP C67Y variant. **c** *Mm* HCP C67S variant. **d** *Mm* HCP C67A variant. Black and red lines indicate the as-isolated and dithionite-reduced forms, respectively. Protein concentration for each protein sample was 40 µM. Concentration of sodium dithionite used for reduction was 1 mM. The UV–visible spectra were recorded using a quartz cuvette with a 1cm light path.

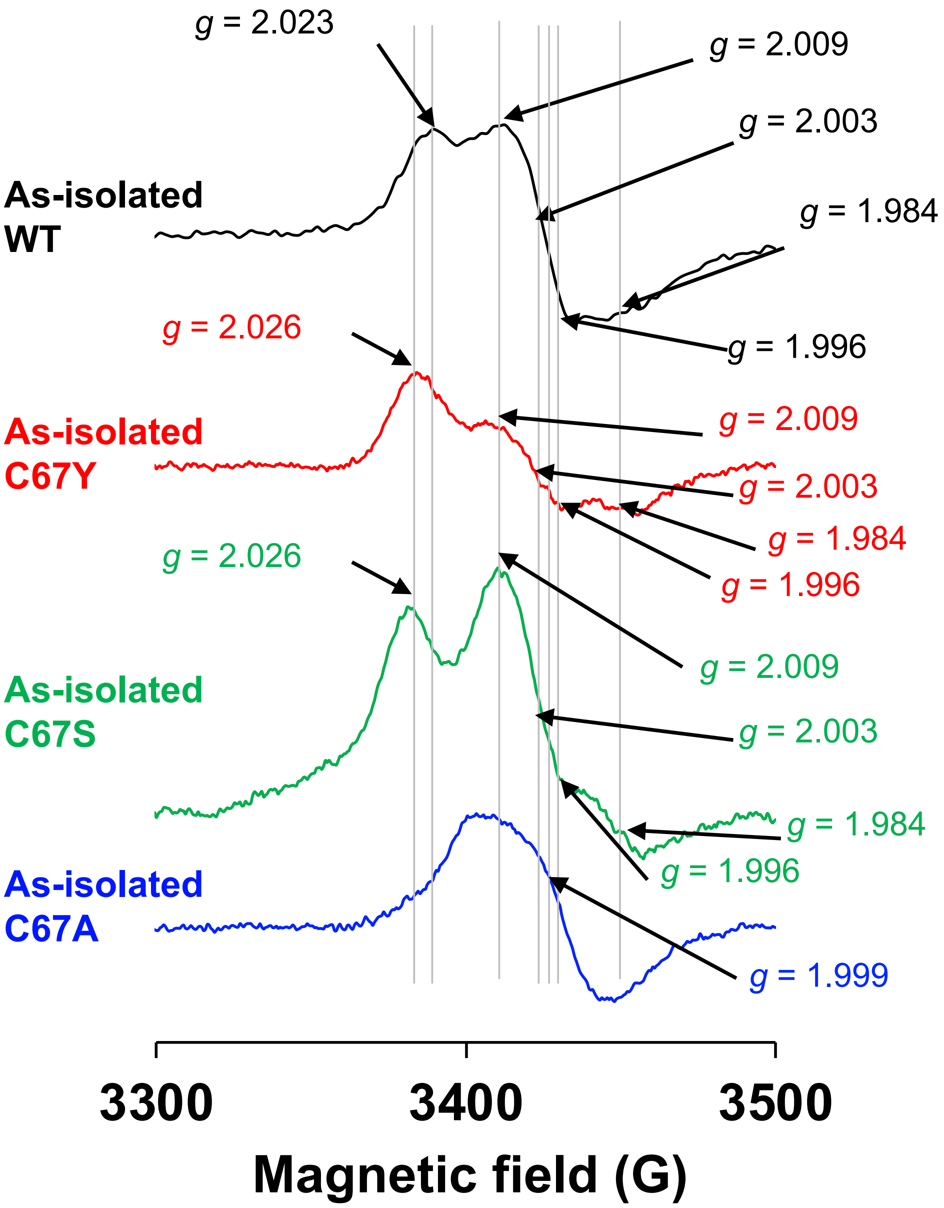

Supplementary Fig. 20. EPR spectra of the as-isolated *Mm* HCP WT and variants in the magnetic field 3300–3500 G with an indication of the *g*-values. EPR spectra of *Mm* HCP WT and the C67Y, C67S and C67A variants are indicated using black, red, green and blue lines, respectively. Inserted grey lines are used to compare the EPR signals and their *g*-values. Black arrows indicate the EPR signals and labels indicate the *g*-values.

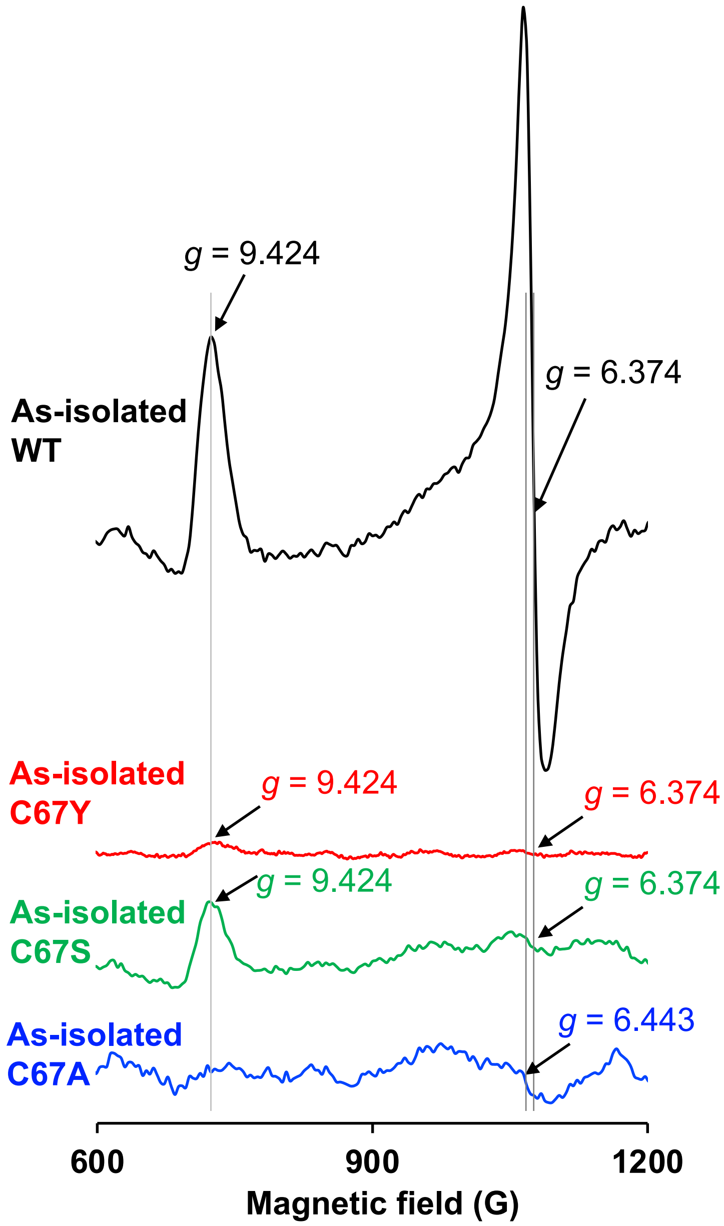

Supplementary Fig. 21. EPR spectra of the as-isolated *Mm* HCP WT and variants in a low magnetic field with indications of *g*-values. EPR spectra of *Mm* HCP WT and the C67Y, C67S and C67A variants are indicated using black, red, green and blue lines, respectively. Inserted grey lines are used to compare the EPR signals and their *g*-values. Black arrows indicate the EPR signals and labels indicate the *g*-values.

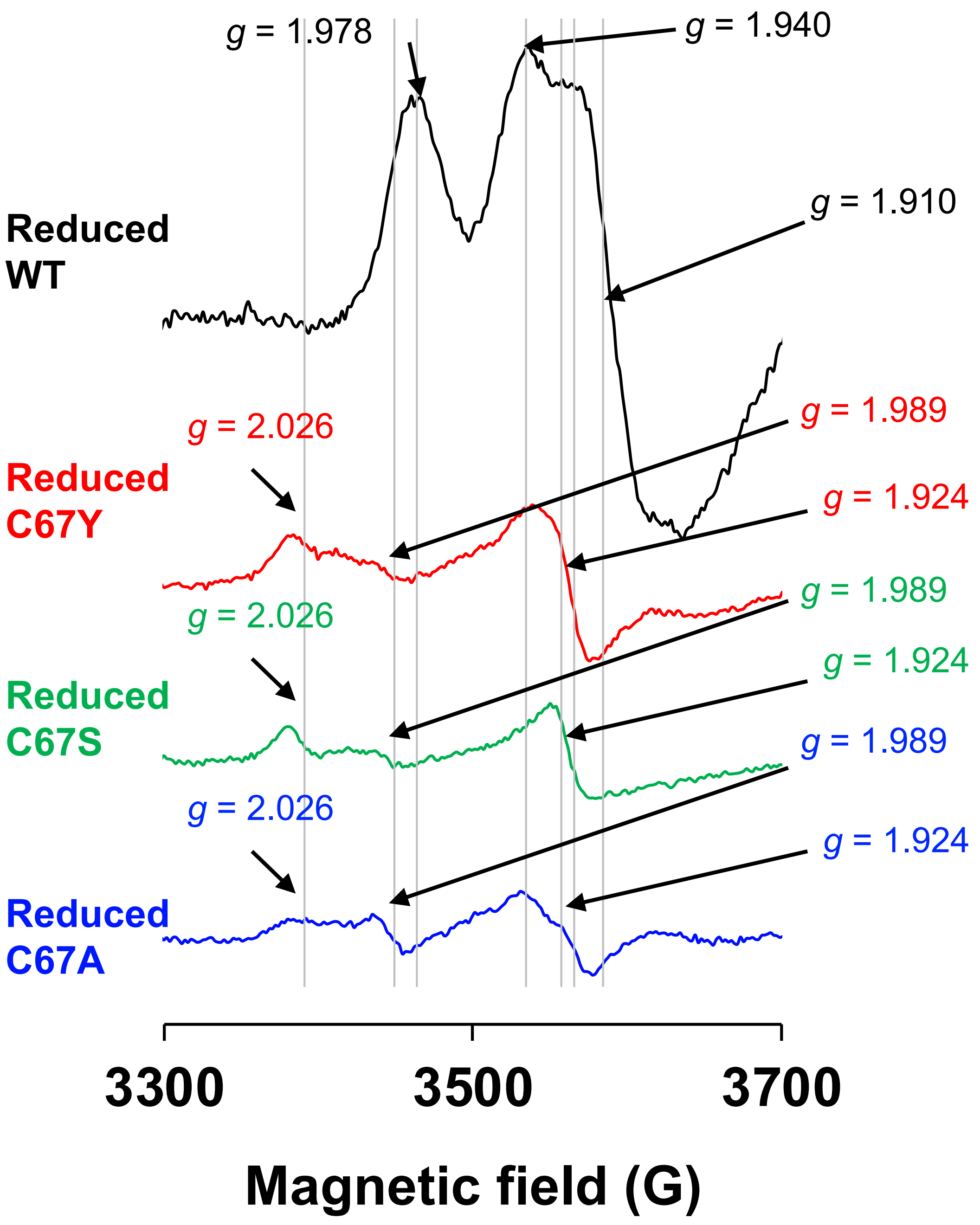

Supplementary Fig. 22. EPR spectra of the dithionite-reduced *Mm* HCP WT and variants in a low magnetic field with indications of the *g*-values. EPR spectra of *Mm* HCP WT and the C67Y, C67S and C67A variants are indicated using black, red, green and blue lines, respectively. Inserted grey lines are used to compare the EPR signals and their *g*-values. Black arrows indicate the EPR signals and labels indicate the *g*-values.

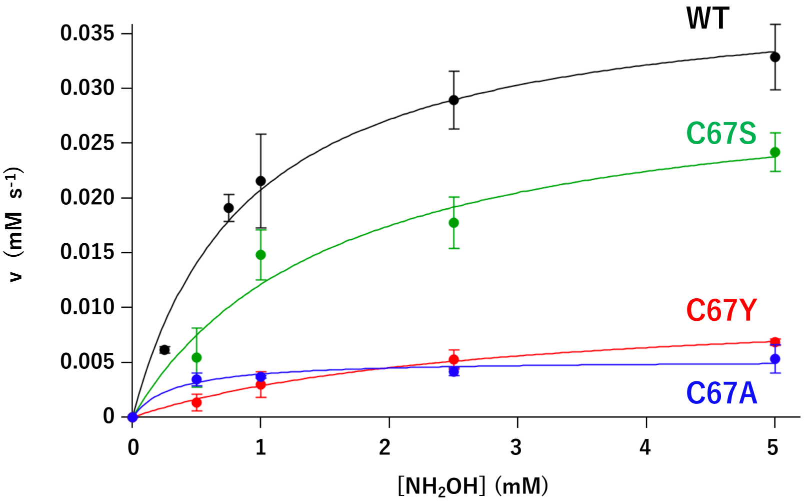

Supplementary Fig. 23. **Michaelis–Menten plot showing the hydroxylamine reductase activity of *Mm* HCP WT and variants.** Curve fitting was performed using Igor Pro 8.0 software (WaveMetrics, Inc., Lake Oswego, OR. USA). Reactions at each point were performed at least three times, and standard deviations are shown as error bars (SD, n = 3 independent activity measurements for each point).

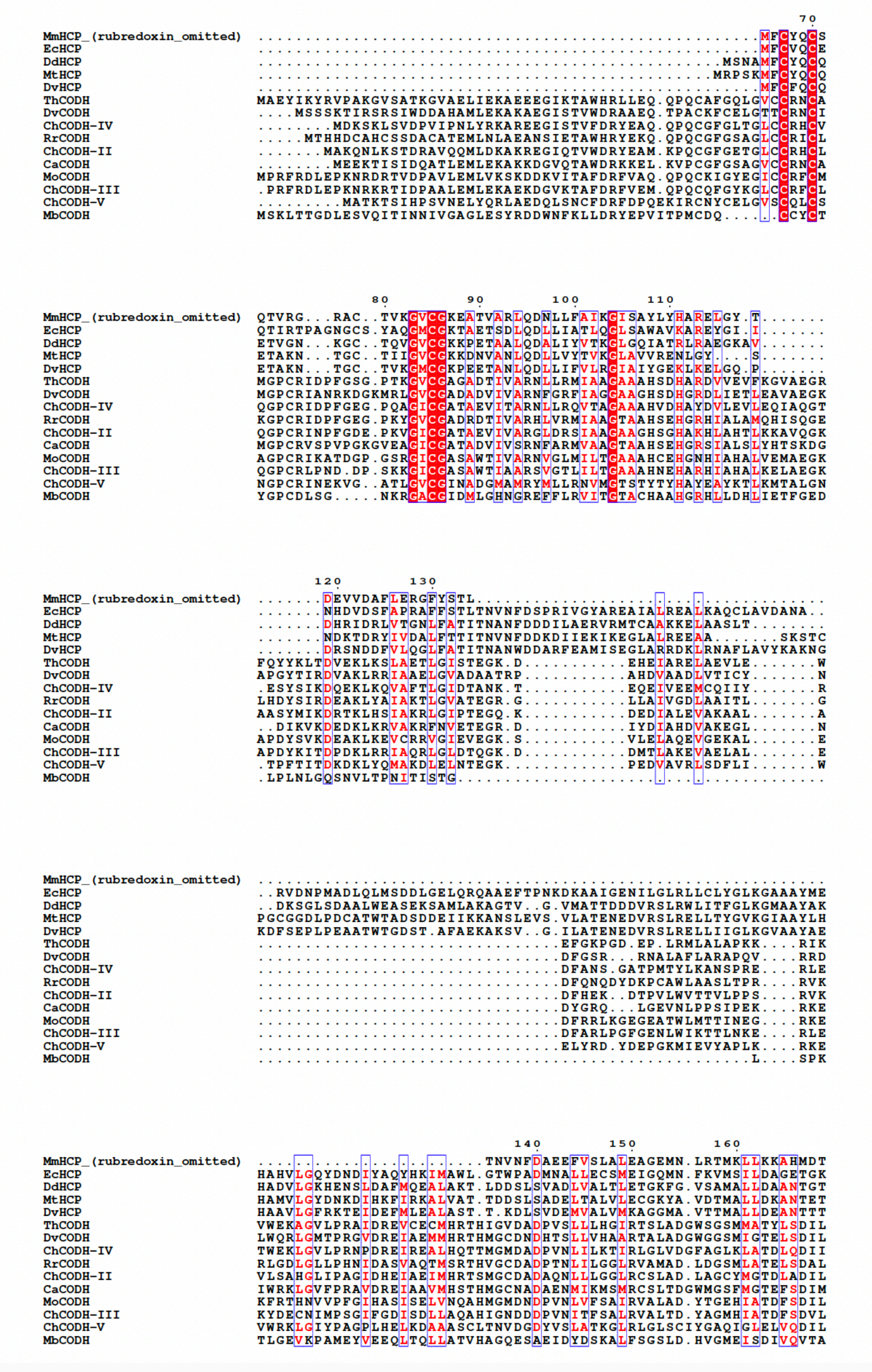
70
MmHCP_(rubredoxin_omitted) ....MFCYQCS
EcHCP ....MFCVQCE
DdHCP ....MSNAMFCYQCQ
MtHCP ....MRPSKMFCYQCQ
DvHCP ....MFCFQCQ
ThCODH MAEYIKYRVPAKGVSATKGVAELIEKAEEEGIKTAWHRLLEQ.QPQCAFGQLGVCCRNCA
DvCODH ....MSSSKTIRSRSIWDDAHAMLEKAKAEGISTVWDRAAEQ.TPACKFCELGTTCRNCI
ChCODH-IV ........MDKSKLSVDPVIPNLYRKAREEGISTVFDRYEAQ.QPQCGFGLTGLCCRHCV
RrCODH .....MTHHDCAHCSSDACATEMLNLAEANSIETAWHRYEKQ.QPQCGFGSAGLCCRICL
ChCODH-II .......MAKQNLKSTDRAVQQMLDKAKREGIQTVWDRYEAM.KPQCGFGETGLCCRHCL
CaCODH ........MEEKTISIDQATLEMLEKAKKDGVQTAWDRKKEL.KVPCGFGSAGVCCRNCA
MoCODH MPRFRDLEPKNRDRTVDPAVLEMLVKSKDDKVITAFDRFVAQ.QPQCKIGYEGICCRFCM
ChCODH-III .PRFRDLEPKNRKRTIDPAALEMLEKAEKDGVKTAFDRFVEM.QPQCQFGYKGLCCRFCL
ChCODH-V .........MATKTSIHPSVNELYQRLAEDQLSNCFDRFDPQEKIRCNYCELGVSCQLCS
MbCODH MSKLTTGDLESVQITINNIVGAGLESYRDDWNFKLLDRYEPVITPMCDQ......CCYCT
80 90 100 110
MmHCP_(rubredoxin_omitted) QTVRG...RAC..TVKGVCCKEATVARLQDNLLFAIKGISAYLYHARELGY.T.......
EcHCP QTIRTPAGNGCS.YAQGMCCKTAETSDLQDLLIATLQGLSAWAVKAREYGI.I.......
DdHCP ETVGN...KGC..TQVGVCCKKPETAALQDALIYVTKGLGQIATRLRAEGKAV.......
MtHCP ETAKN...TGC..TIIGVCCKKDNVAANLQDLLVYTVKGLAVVRENLGY....S.......
DvHCP ETAKN...TGC..TVKGMCCKPEETANLQDLLIFVLRGIAIYGEKLKELGQ.P.......
ThCODH MGPCRIDPFGSG.PTKGVCCAGADTIVARNLLRMIAAGAAAHSDHARDVVEVFKGVAEGR
DvCODH MGPCRIANRKDGKMRLGVCCADADVIVARNFGRFIAGGAAGHSDHGRDLIETLEAVAEGK
ChCODH-IV QGPCRIDPFGEG.PQAGICCATAEVITARNLLRQVTAGAAAHVDHAYDVLEVLEQIAQGT
RrCODH KGPCRIDPFGEG.PKYGVCCADRDTIVARHLVRMIAAGTAAHSEHGRHIALAMQHISQGE
ChCODH-II QGPCRINPFGDE.PKVGICCATAEVIVARGLDRSIAAGAAGHSGHAKHLAHTLKKAVQGK
CaCODH MGPCRVSPVPGKGVEAGICCATADVIVSRNFARMVAAGTAAHSEHGRSIALSLYHTSKDG
MoCODH AGPCRIKATDGP.GSRGICCASAWTIVARNVGLMILTGAAAHCEHGNHIAHALVEMAEGK
ChCODH-III QGPCRLPND.DP.SKKGICCASAWTIAARSVGTLILTGAAAHNEHARHIAHALKELAEGK
ChCODH-V NGPCRINEKVG..ATLGVCCINADGMAMRYMLLRNVMGTSTYTYHAYEAYKTLKMTALGN
MbCODH YGPCDLSG.....NKRGACCIDMLGHNGREFFLRVITGTACHAAHGRHLLDHLIETFGED
120 130
MmHCP_(rubredoxin_omitted) .......DEVVDAFLERGFYSTL.....................................
EcHCP .......NHDVDSFAPRAFFSTLTNVNFDSPRIVGYAREAIALREALKAQCLAVDANA..
DdHCP .......DHRIDRLVTGNLFATITNANFDDDILAERVRMTCAAKKELAASLT........
MtHCP .......NDKTDRYIVDALFTTITNVNFDDKDIIEKIKEGLALREEAA.......SKSTC
DvHCP .......DRSNDDFVLQGLFATITNANWDDARFEAMISEGLARRDKLRNAFLAVYKAKNG
ThCODH FQYYKLTDVEKLKSLAETLGISTEGK.D..........EHEIARELAEVLE.......W
DvCODH APGYTIRDVAKLRRIAAELGVADAATRP...........AHDVAADLVTICY.......N
ChCODH-IV .ESYSIKDQEKLKQVAFTLGIDTANK.T...........EQEIVEEMCQIIY.......R
RrCODH LHDYSIRDEAKLYAIAKTLGVATEGR.G...........LLAIVGDLAAITL.......G
ChCODH-II AASYMIKDRTKLHSIAKRLGIPTEGQ.K...........DEDIALEVAKAAL.......A
CaCODH ..DIKVKDEDKLKRVAKRFNVETEGR.D...........IYDIAHDVAKEGL.......N
MoCODH APDYSVKDEAKLKEVCRRVGIEVEGK.S...........VLELAQEVGEKAL.......E
ChCODH-III APDYKITDPDKLRRIAQRLGLDTQGK.D...........DMTLAKEVAELAL.......E
ChCODH-V .TPFTITDKDKLYQMAKDLELNTEGK.............PEDVAVRLSDFLI.......W
MbCODH .LPLNLGQSNVLTPNITISTG.......................................
MmHCP_(rubredoxin_omitted) ....
EcHCP ..RVDNPMADLQLMSDDLGELQRQAAEFTPNKDKAAIGENILGLRLLCLYGLKGAAAYME
DdHCP ..DKSGLSDAALWEASEKSAMLAKAGTV..G.VMATTDDDVRSLRWLITFGLKGMAAYAK
MtHCP PGCGGDLPDCATWTADSDDEIIKKANSLEVS.VLATENEDVRSLRELLTYGVKGIAAYLH
DvHCP KDFSEPLPEAATWTGDST.AFAEKAKSV..G.ILATENEDVRSLRELLIIGLKGVAAYAE
ThCODH ................................EFGKPGD.EP.LRMLALAPKK....RIK
DvCODH ................................DFGSR...RNALAFLARAPQV....RRD
ChCODH-IV ................................DFANS.GATPMTYLKANSPRE....RLE
RrCODH ................................DFQNQDYDKPCAWLAASLTPR....RVK
ChCODH-II ................................DFHEK.DTPVLWVTTVLPPS....RVK
CaCODH ................................DYGRQ...LGEVNLPPSIPEK....RKE
MoCODH ................................DFRRLKGEGEATWLMTTINEG....RKE
ChCODH-III ................................DFARLPGFGENLWIKTTLNKE....RLE
ChCODH-V ................................ELYRD.YDEPGKMIEVYAPLK....RKE
MbCODH ....L....SPK
140 150 160
MmHCP_(rubredoxin_omitted) ..............TNVNFDAEEFVSLALEAGEMN.LRTMKLLKKAHMDT
EcHCP HAHVLGQYDNDIYAQYHKIMAWL.GTWPADMNALLECSMEIGQMN.FKVMSILDAGETGK
DdHCP HADVLGKHENSLDAFMQEALAKT.LDDSLSVADLVALTLETGKFG.VSAMALLDAANTGT
MtHCP HAMVLGYDNKDIHKFIRKALVAT.TDDSLSADELTALVLECGKYA.VDTMALLDKANTET
DvHCP HAAVLGFRKTEIDEFMLEALAST.T.KDLSVDEMVALVMKAGGMA.VTTMALLDEANTTT
ThCODH VWEKAGVLPRAIDREVCECMHRTHIGVDADPVSLLLHGIRTSLADGWSGSMMATYLSDIL
DvCODH LWQRLGMTPRGVDREIAEMMHRTHMGCDNDHTSLLVHAARTALADGWGGSMIGTELSDIL
ChCODH-IV TWEKLGVLPRNPDREIREALHQTTMGMDADPVNLILKTIRLGLVDGFAGLKLATDLQDII
RrCODH RLGDLGLLPHNIDASVAQTMSRTHVGCDADPTNLILGGLRVAMAD.LDGSMLATELSDAL
ChCODH-II VLSAHGLIPAGIDHEIAEIMHRTSMGCDADAQNLLLGGLRCSLAD.LAGCYMGTDLADIL
CaCODH IWRKLGVFPRAVDREIAAVMHSTHMGCNADAENMIKMSMRCSLTDGWMGSFMGTEFSDIM
MoCODH KFRTHNVVPFGIHASISELVNQAHMGMDNDPVNLVFSAIRVALAD.YTGEHIATDFSDIL
ChCODH-III KYDECNIMPSGIFGDISDLLAQAHIGNDDDPVNITFSALRVALTD.YAGMHIATDFSDVL
ChCODH-V VWRKLGIYPAGPLHELKDAAASCLTNVDGDYVSLATKGLRLGLSCIYGAQIGLELVQDIL
MbCODH TLGEVKPAMEYVEEQLTQLLATVHAGQESAEIDYDSKALFSGSLD.HVGMEISDIVQVTA

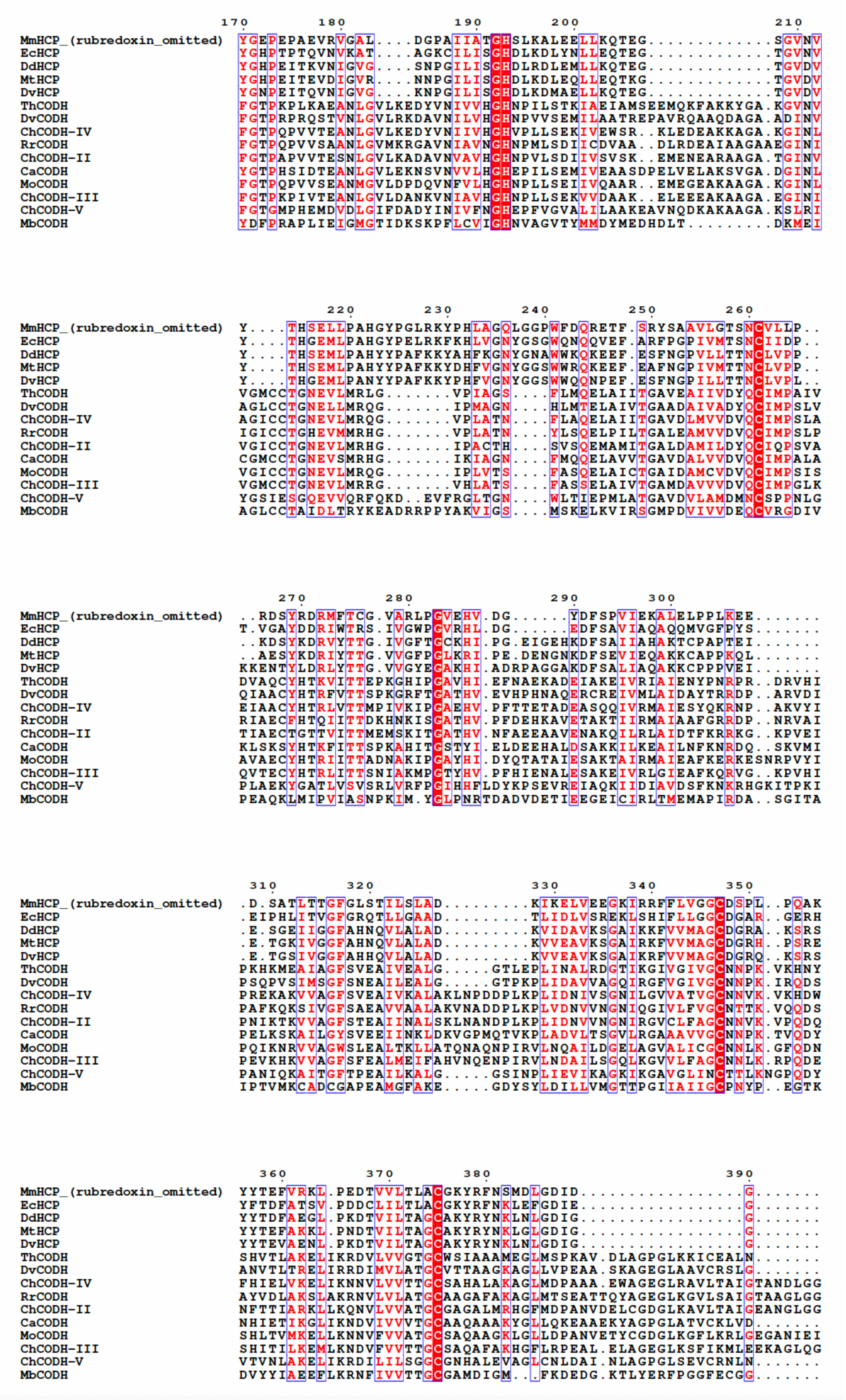

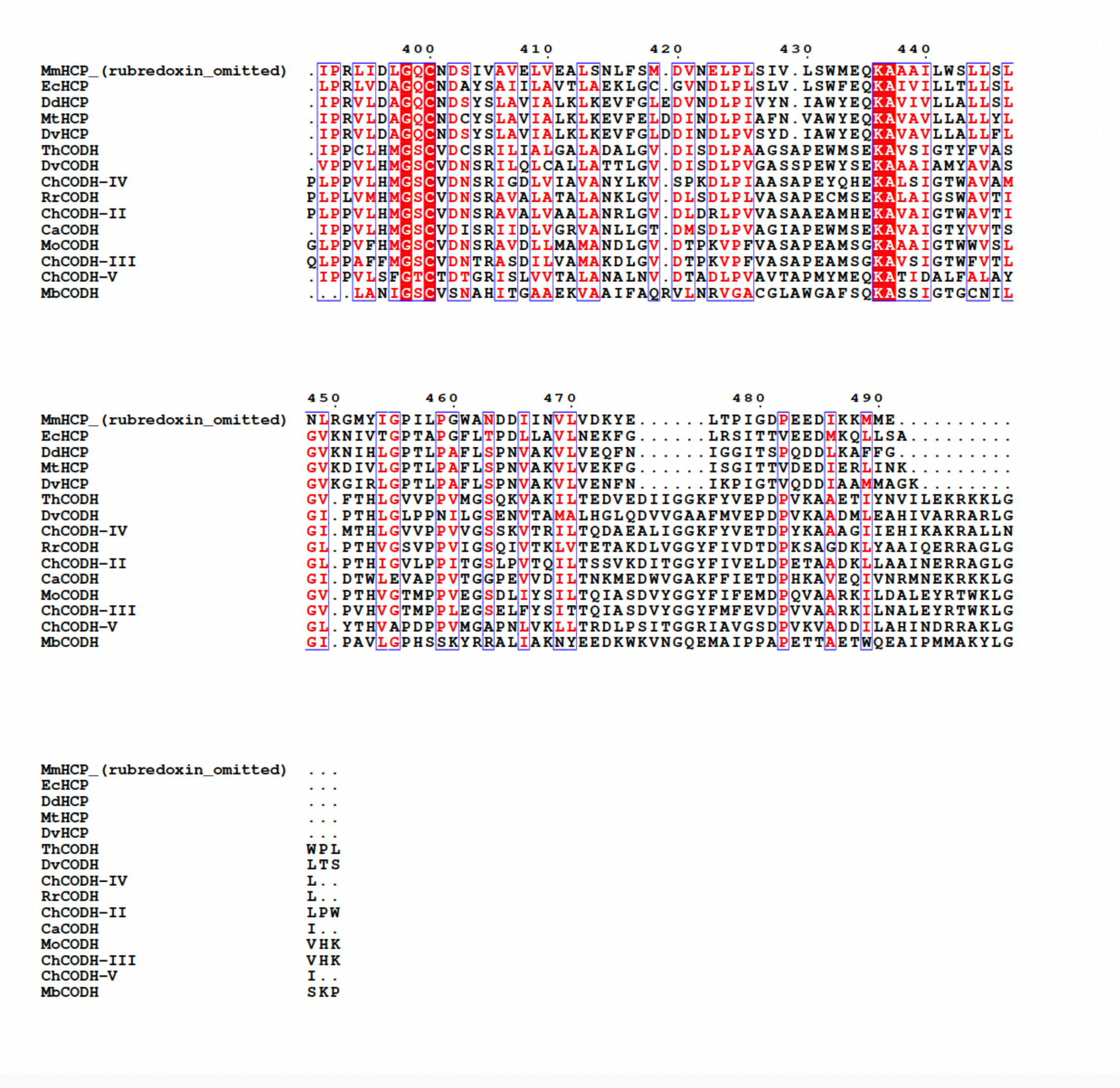

Supplementary Fig. 24. Amino acid sequence alignments guided by structural information. The alignments used for creation of the structure-guided phylogenetic tree were created using PROMALS3D[20]. The figure of the alignments was created using ESpript3[6]. White letters in the red box background indicate strictly conserved residues. The red bold letters in the blue box indicate well-conserved or similar residues. It is noted that the N-terminal rubredoxin domain with linker in *Mm H*CP WT is deleted for the alignments because this domain was not structurally aligned to the overall structures of CODHs. The middle domain specifically found in class I and II HCPs are not deleted for the alignments to consider the importance of this domain for the evolutionary relationship of all classes of HCPs and CODHs. The numbers above the alignments indicate the number of the residues of *Mm* HCP. MmHCP_(rubredoxin_omitted), *M. marburgensis* HCP without the N-terminal rubredoxin domain-containing region; EcHCP, *E. coli* HCP; DdHCP, *D. desulfuricans* HCP; MtHCP, *M. thermolithotrophicus* HCP; DvHCP, *D. vulgaris* HCP; ThCODH, *Thermococcus* sp. AM4 CODH; DvCODH, *D. vulgaris* CODH; ChCODH-IV, *C.*

hydrogenoformus CODH-IV; RrCODH, *R. rubrum* CODH; ChCODH-II, C. *hydrogenoformus* CODH-II; CaCODH, C. *autoethanogenum* CODH; MoCODH, *M. thermoacetica* CODH; ChCODH-III, C. *hydrogenoformus* CODH-III; ChCODH-V, *C. hydrogenoformus* CODH-V; MbCODH, *M. barkeri* CODH.

Supplementary Table 1. List of primers used in this study

Primer	Sequence*
NdeI-MmHCP-F	5′-AAAACATATGAAGTACCGCTGCAAGGTATGTGAC -3′
SalI-MmHCP-R	5′-ATTGTCGACCTCCATCATTTTCTTTATATCCTCTTCAGGGTCA-3′
stop-1-52-MmRubredoxin-rev	5′-AAATTAACCCCTGAGGGGCTCAAACTGGTCCTTC -3′
53- MmRubredoxin-for	5′-AAGAAGGAGATATAATGGAGGTGAGGCGCGTGAGGCCAGAGG-3′
C67S-mmHCP-for	5′-ATGTTCAGCTACCAGTGCTCCCAGACCGTCCGTGGAAGGGCC-3′
C67A-mmHCP-for	5′-ATGTTCGCCTACCAGTGCTCCCAGACCGTCCGTGGAAGGGCC-3′
C67S-or-A-mmHCP-rev	5′-GTCTATATCCTCTGGCCTCACGCGCCTCACCTCAC-3′

*Restriction sites used for cloning are underlined. Mutagenic codons are double underlined.

Supplementary Table 2. Compositions of the crystallization drops

Protein	Concentration of the protein stock solution	Crystallization mother liquor	Protein stock solution/crystallization mother liquor in the drop
Mm HCP WT	18 mg/mL	30% (w/v) PEG4000, 0.1 M Tris–HCl, pH 8.5, and 200 mM sodium acetate	1 µL/1 µL
Mm HCP C67Y variant	27 mg/mL	0.05 M HEPES–NaOH, pH 7.0, 0.1 M ammonium acetate, 0.02 M $MgCl_2$ and 5% (w/v) PEG8000	1 µL/1 µL

Supplementary Table 3. X-ray data collection and refinement statistics

	Mm HCP WT	*Mm* HCP C67Y
**Data collection**		
Space group	$P4_3$	$P2_1$
Cell dimensions		
a, *b*, *c* (Å)	68.0, 68.0, 469.3	64.9, 70.6, 106.3
α, β, γ (°)	90.0, 90.0, 90.0	90.0, 106.9, 90.0
Resolution (Å)	48.05–2.82 (2.92–2.82)*	47.29–3.00 (3.11–3.00)*
R_{sym}	0.185 (0.951)*	0.123 (0.979)*
R_{meas}	0.193 (0.994)	0.131 (1.040)*
$I / \sigma I$	9.9 (2.2)*	11.5 (2.0)*
Completeness (%)	100.0 (100.0)*	99.6 (98.9)*
$CC_{1/2}$	0.988 (0.742)*	0.997 (0.736)*
Redundancy	12.4 (11.9)*	8.1 (8.7)*
No. total reflections	629452 (59876)*	151031 (16082)*
No. unique reflections	50758 (5050)*	18596 (1843)*
**Refinement**		
Resolution (Å)	48.05–2.82 (2.92–2.82)*	47.29–3.00 (3.11–3.00)*
No. reflections	50756 (5050)*	18581 (1839)*
No. reflections used for R_{free}	2554 (262)*	929 (92)*
R_{work} / R_{free}	0.205/0.260 (0.328/0.340)*	0.190/0.260 (0.295/0.409)*
No. atoms		
Protein	15241	6296
Ligand/ion	72	18
Water	40	2
B-factors (Å^2)		
Protein	69.84	89.10
Ligand/ion	83.13	104.98
Water	47.32	78.5
R.m.s. deviations		
Bond lengths (Å)	0.004	0.004
Bond angles (°)	0.83	0.85

*Values in parentheses are for highest-resolution shell.

Supplementary References

1. Fujishiro, T., Ooi, M. & Takaoka, K. Crystal structure of *Escherichia coli* class II hybrid cluster protein, HCP, reveals a [4Fe-4S] cluster at the N-terminal protrusion. *FEBS J.* **288**, 6752–6768 (2021).

2. Lemaire, O. N., Belhamri, M., & Wagner, T. Structural and biochemical elucidation of class I hybrid cluster protein natively extracted from a marine methanogenic archaeon. *Front. Microbiol.* **14**, 1179204 (2023).

3. Aragão, D., Mitchell, E. P., Frazão, C. F., Carrondo, M. A. & Lindley, P. F. Structural and functional relationships in the hybrid cluster protein family: structure of the anaerobically purified hybrid cluster protein from *Desulfovibrio vulgaris* at 1.35 Å resolution. *Acta Crystallogr. D Biol. Crystallogr.* **64**, 665–674 (2008).

4. Macedo, S., Aragão, D., Mitchell, E. P. & Lindley, P. Structure of the hybrid cluster protein (HCP) from *Desulfovibrio desulfuricans* ATCC 27774 containing molecules in the oxidized and reduced states. *Acta Crystallogr. D Biol. Crystallogr.* **59**, 2065–2071 (2003).

5. Sievers, F. & Higgins, D. G. Clustal Omega for making accurate alignments of many protein sequences. *Protein Sci*. **27**, 135–145 (2018).

6. Robert, X. & Gouet, P. Deciphering key features in protein structures with the new ENDscript server. *Nucleic Acids Res.* **42**, W320–W324 (2014).

7. Wittenborn, E. C., et al. Redox-dependent rearrangements of the NiFeS cluster of carbon monoxide dehydrogenase. *eLife* **7**, e39451 (2018).

8. Jeoung, J. H. & Dobbek, H. Carbon dioxide activation at the Ni,Fe-cluster of anaerobic carbon monoxide dehydrogenase. *Science* **318**, 1461–1464 (2007).

9. Ruickoldt, J., Basak, Y., Domnik, L., Jeoung, J.-H. & Dobbek, H. On the kinetics of CO_2 reduction by Ni, Fe-CO dehydrogenases. *ACS Catal.* **12**, 13131–13142 (2022).

10. Domnik, L., et al. CODH-IV: A high-efficiency CO-scavenging CO dehydrogenase with resistance to O_2. *Angew. Chem. Int. Ed.* **56**, 15466–15469 (2017).

11. Doukov, T. I., Iverson, T. M., Seravalli, J., Ragsdale, S. W. & Drennan, C. L. A Ni-Fe-Cu center in a bifunctional carbon monoxide dehydrogenase/acetyl-CoA synthase. *Science* **298**, 567–572 (2002).

12. Lemaire, O. N. & Wagner, T. Gas channel rerouting in a primordial enzyme: structural insights of the carbon-monoxide dehydrogenase/acetyl-CoA synthase complex from the acetogen *Clostridium autoethanogenum*. *Biochim. Biophys. Acta Bioenerg.* **1862**, 148330 (2021).

13. Drennan, C. L., Heo, J., Sintchak, M. D., Schreiter, E. & Ludden, P. W. Life on carbon monoxide: X-ray structure of *Rhodospirillum rubrum* Ni-Fe-S carbon monoxide dehydrogenase. *Proc. Natl Acad. Sci. U. S. A.* 98, 11973–11978 (2001).

14. Benvenuti, M., et al. The two CO-dehydrogenases of *Thermococcus* sp. AM4. *Biochim. Biophys. Acta Bioenerg.* **1861**, 148188 (2020).

15. Jeoung, J. H., et al. A morphing [4Fe-3S-nO]-cluster within a carbon monoxide dehydrogenase scaffold. *Angew. Chem. Int. Ed.* **61**, e202117000 (2022).

16. Gong, W., et al. Structure of the $\alpha_2\varepsilon_2$ Ni-dependent CO dehydrogenase component of the *Methanosarcina barkeri* acetyl-CoA decarbonylase/synthase complex. *Proc. Natl Acad. Sci. U. S. A.* **105**, 9558–9563 (2008).

17. Can, M., Armstrong, F. A. & Ragsdale, S. W. Structure, function, and mechanism of the nickel metalloenzymes, CO dehydrogenase, and acetyl-CoA synthase. *Chem. Rev.* **114**, 4149–4174 (2014).

18. Laskowski, R. A., Jabłońska, J., Pravda, L., Vařeková, R. S. & Thornton, J. M. PDBsum: structural summaries of PDB entries. *Protein Sci.* **27**, 129–134 (2018).

19. Waterhouse, A*., et al.* SWISS-MODEL: homology modelling of protein structures and complexes. *Nucl. Acids Res.* **46**, W296–W303 (2018).

20. Pei, J., Kim, B.-F., Grishin, N. V. PROMALS3D: a tool for multiple protein sequence and structure alignments. *Nucl*. *Acids Res*. **36**, 2295–2300 (2008).
