# Supplementary material for: Class III hybrid cluster protein homodimeric architecture shows evolutionary relationship with Ni, Fe-carbon monoxide dehydrogenases
Source: Nat Commun. 2023 Sep 14;14:5609. doi: 10.1038/s41467-023-41289-4 (PMC10502027; doi:10.1038/s41467-023-41289-4)
Supplement: Supplementary file 2 — Reporting Summary [file 41467_2023_41289_MOESM2_ESM.pdf]

nature portfolio

Corresponding author(s): Takashi Fujishiro

Last updated by author(s): Aug 12, 2023

Reporting Summary

Nature Portfolio wishes to improve the reproducibility of the work that we publish. This form provides structure for consistency and transparency in reporting. For further information on Nature Portfolio policies, see our Editorial Policies and the Editorial Policy Checklist.

Statistics

For all statistical analyses, confirm that the following items are present in the figure legend, table legend, main text, or Methods section.

n/a	Confirmed	
☐	☒	The exact sample size (*n*) for each experimental group/condition, given as a discrete number and unit of measurement
☒	☐	A statement on whether measurements were taken from distinct samples or whether the same sample was measured repeatedly
☒	☐	The statistical test(s) used AND whether they are one- or two-sided<br>*Only common tests should be described solely by name; describe more complex techniques in the Methods section.*
☒	☐	A description of all covariates tested
☒	☐	A description of any assumptions or corrections, such as tests of normality and adjustment for multiple comparisons
☐	☒	A full description of the statistical parameters including central tendency (e.g. means) or other basic estimates (e.g. regression coefficient) AND variation (e.g. standard deviation) or associated estimates of uncertainty (e.g. confidence intervals)
☒	☐	For null hypothesis testing, the test statistic (e.g. *F*, *t*, *r*) with confidence intervals, effect sizes, degrees of freedom and *P* value noted<br>*Give P values as exact values whenever suitable.*
☒	☐	For Bayesian analysis, information on the choice of priors and Markov chain Monte Carlo settings
☒	☐	For hierarchical and complex designs, identification of the appropriate level for tests and full reporting of outcomes
☒	☐	Estimates of effect sizes (e.g. Cohen's *d*, Pearson's *r*), indicating how they were calculated

Our web collection on statistics for biologists contains articles on many of the points above.

Software and code

Policy information about availability of computer code

Data collection	Dali server
Data analysis	Igor Pro. 8.0, XDS Package, open-source PyMOL ver. 1.7, MEGA11, PROMALS3D, Clustal Omega, ESPript3 ,MolProbity, Coot ver.0.8.9.2 , Refmac5 ver.8.5, Phenix_refine ver.1.20.1_4487

For manuscripts utilizing custom algorithms or software that are central to the research but not yet described in published literature, software must be made available to editors and reviewers. We strongly encourage code deposition in a community repository (e.g. GitHub). See the Nature Portfolio guidelines for submitting code & software for further information.

Data

Policy information about availability of data

All manuscripts must include a data availability statement. This statement should provide the following information, where applicable:

- Accession codes, unique identifiers, or web links for publicly available datasets
- A description of any restrictions on data availability
- For clinical datasets or third party data, please ensure that the statement adheres to our policy

Data regarding the coordination and structural factors of Mm HCP WT and the C67Y variant are available in the Protein Data Bank (PDB) under the accession codes 7E0L (Mm HCP WT) and 7WSX (Mm HCP C67Y variant), respectively. The URL links of the accession codes used in this study (new ones as well as previously published ones) are as follows: 7E0L [https://www.rcsb.org/structure/7E0L] (Mm HCP WT), 7WSX [https://www.rcsb.org/structure/7WSX] (Mm HCP C67Y variant),

1W9M [https://www.rcsb.org/structure/1W9M] (Dv HCP), 1BRF [https://www.rcsb.org/structure/1BRF] (Pf rubredoxin) and 3B51 [https://www.rcsb.org/structure/3B51] (Ch CODH-II).

Research involving human participants, their data, or biological material

Policy information about studies with human participants or human data. See also policy information about sex, gender (identity/presentation), and sexual orientation and race, ethnicity and racism.

Reporting on sex and gender	n/a
Reporting on race, ethnicity, or other socially relevant groupings	n/a
Population characteristics	n/a
Recruitment	n/a
Ethics oversight	n/a

Note that full information on the approval of the study protocol must also be provided in the manuscript.

Field-specific reporting

Please select the one below that is the best fit for your research. If you are not sure, read the appropriate sections before making your selection.

☒ Life sciences ☐ Behavioural & social sciences ☐ Ecological, evolutionary & environmental sciences

For a reference copy of the document with all sections, see nature.com/documents/nr-reporting-summary-flat.pdf

Life sciences study design

All studies must disclose on these points even when the disclosure is negative.

Sample size	n = 3 was used, because individual experiments by three times gave the data enough to perform the Micaelis-Menten kinetic analysis via curve-fitting with Igor software.
Data exclusions	No data exclusion
Replication	Reproducibility of all the experiments except for C67Y spontaneous mutation were possible. The spontaneous mutation may have occurred because of direct use of M. marburgensis cells for PCR amilification of hcp gene (non-pure genomic DNA). This discussion was already added in the sub-section of "Structure of the Mm HCP C67Y variant" in "Results and Discussion".
Randomization	Rfree calculation is based on choosing 5% of reflection data randomly as usual in X-ray crystallography. For other experiments such as UV and EPR measurements and activity assays, no randomization processes were needed.
Blinding	No experiments to need blinding process because all experiments based on biochemistry and structural biology were done in vitro.

Reporting for specific materials, systems and methods

We require information from authors about some types of materials, experimental systems and methods used in many studies. Here, indicate whether each material, system or method listed is relevant to your study. If you are not sure if a list item applies to your research, read the appropriate section before selecting a response.

Materials & experimental systems

n/a	Involved in the study	
☒	☐	Antibodies
☒	☐	Eukaryotic cell lines
☒	☐	Palaeontology and archaeology
☒	☐	Animals and other organisms
☒	☐	Clinical data
☒	☐	Dual use research of concern
☒	☐	Plants

Methods

n/a	Involved in the study	
☒	☐	ChIP-seq
☒	☐	Flow cytometry
☒	☐	MRI-based neuroimaging
